# Recent Advances in Microencapsulated Phase Change Materials for Energy Efficiency in Buildings: A Review

**DOI:** 10.3390/polym18040451

**Published:** 2026-02-10

**Authors:** Andrea I. Bardales-Cortés, Joan Formosa, Jessica Giro-Paloma

**Affiliations:** DIOPMA Research Group, Departament de Ciència de Materials i Química Física, Universitat de Barcelona, Martí i Franquès 1-11, 08028 Barcelona, Spain; aibardales@ub.edu (A.I.B.-C.); joanformosa@ub.edu (J.F.)

**Keywords:** energy efficiency, building simulation, life cycle assessment, latent heat storage, environmental impacts, sustainability

## Abstract

Phase change materials (PCMs) have attracted significant attention for their capacity to store and release large amounts of latent heat in response to ambient temperature variations, offering an effective strategy for thermal regulation in buildings. Meanwhile, recent research is focused on microencapsulated phase change materials (MPCMs), which provide enhanced thermal efficiency, improved stability, and easier integration into construction materials. This study stands apart from offering a structured comparative analysis of PCM and MPCM systems. Using detailed synthesis tables, the review systematically evaluates materials, encapsulation approaches, and performance indicators. The review presents an integrative framework that correlates materials’ thermophysical properties with specialized simulation software and region-specific climatic conditions. MPCMs are assessed in terms of composition, phase change characteristics, and encapsulation techniques, with complex information condensed into practical selection criteria. Furthermore, MPCM products covering phase change temperature ranges from 18 °C to 32 °C are systematically aligned with specific climate zones and life cycle assessment outcomes, offering a clear framework for optimization. The polymers play a vital role in MPCM technology, and their applications for buildings have been studied thoroughly. This work also aims to guide research and development toward scalable, energy-efficient, and sustainable building technologies for both academic and industrial stakeholders.

## 1. Introduction

In the 21st century, global warming and climate change have become a menace for the progress and advancement of humankind [1]. Energy demands are rising continuously because of economic development, globalization, population growth, and advancement in living standards. Therefore, energy demand has become the primary cause of threat [2,3]. The European Union (EU) can make an enormous contribution by reducing energy consumption, which in turn improves energy efficiency, by as much as half in comparison to 2005, and will play a central role in accomplishing Net-Zero Greenhouse Gas (GHG) emissions by 2050 [4,5]. In addition, according to the International Energy Agency (IEA) the building sector contributes over one third of global energy consumption and emissions, based on construction, heating, ventilation, and air conditioning (HVAC), home lights, and appliances. The building sector is responsible for 30% of global final energy consumption and is responsible for 26% of global energy-related emissions. However, 60% of the energy consumption in buildings is produced by the HVAC system [6].

In these conditions, researchers and policy makers are encouraging new policies in the direction of more sustainable and energy-efficient buildings. They explore potential solutions to enhance energy conservation and energy storage as an effort to reduce global warming issues.

Phase change materials (PCMs) have emerged as effective passive thermal regulators in buildings, helping to maintain stable indoor conditions and reduce energy consumption for heating and cooling. However, their integration into building envelopes presents technical challenges, particularly concerning leakage and stability during phase transitions. Recent advances in shape-stabilized nano/microencapsulated, and macroencapsulated PCMs have improved their durability and ease of incorporation. In this review, the aim is to focus on the PCMs/MPCMs used. Hence, research has enabled PCMs/MPCMs to be successfully applied in walls, roofs, floors, etc., offering significant potential for energy savings and enhanced thermal comfort in modern buildings. By bridging material innovation with sustainable design, the review highlights how PCMs/MPCMs are redefining the future of energy storage and thermal management in modern buildings.

Although polymer incorporation in cementitious materials is widely recognized to negatively affect mechanical performance. In particular, the compressive strength, stiffness, and the integration of polymer-based microencapsulated phase change materials (MPCMs) have introduced a different design paradigm for construction and building applications. MPCM provides added value beyond structural performance by enabling passive thermal energy storage, reducing HVAC demands, and contributing to substantial decreases in operational energy use and associated CO_2_ emissions. These systems are typically composed of a latent heat storage core, often paraffinic waxes, encapsulated within polymeric shells that ensure shape stability, leakage prevention, and chemical protection during mixing and service life. As a result, MPCM-modified cementitious materials represent a complex polymer–ceramic composite whose performance depends strongly on the compatibility between the polymeric shell and the inorganic matrix. The review is majorly focused on LCA simulations (Sphera^®^ LCA for Experts/GaBi 10.7, SimaPro 10.3, OpenLCA 2.6.0 and One Click LCA (https://oneclicklca.com/, accessed 5 February 2026)) and building or Multiphysics simulation software, like TRNSYS 18, Design Builder 7.3, COMSOL Multiphysics^®^ 6.4, EnergyPlus 9.4, etc., to explore the environmental impact, energy savings, energy consumption, thermal performance, novel materials, and polymers. A comprehensive understanding of the chemical, physical, environmental, thermal, and mechanical interactions at the interface is therefore essential to ensure long-term durability, functional efficiency, and structural reliability. Consequently, the study of polymeric shells in MPCM systems is critical not only for optimizing energy-related performance but also for enabling their safe and effective deployment in sustainable construction materials.

### 1.1. Energy Storage (ES)

Electricity has a crucial role to many parts of the modern society, but its production stills depend on fossil fuels as a result of their reliability and efficiency [7,8]. Fossil fuels have a huge impact that directly affects the environment. Fossil fuel price is continuously rising due to increasing consumption, assumed to be doubled in 2050 and tripled by 2100 [9]. Renewable energy sources (RES) are being incorporated to replace the consumption of fossil fuels. RESs can be applied in different fields such as industrial, commercial, residential, and agricultural [10]. The limitation of applying RES is that they are intermittent, have a low load factor, and are non-dispatchable [11]. Therefore, the ES sector must face the immediate development of industrialization and urbanization [12]. Nevertheless, energy consumption is increasing faster than population. Energy storage systems (ESSs) have become a widely developed technology, able to balance the gap between energy demand and supply [13,14]. ESSs can reduce electricity use, can be stored during off-peak times, and used during on-peak times. In addition, there is a reduction in volume of the emitted carbon. Although there are different types of ESSs, categorized based on the stored energy form, this review is based on thermal energy storage (TES) systems.

### 1.2. Thermal Energy Storage (TES)

There is a growing interest in applying ESSs in the building sector, because most of the CO_2_ emissions come mainly from the production of building materials and electricity to offer modern comfort [15,16]. The ES technology enables thermal energy to be stored by heating or cooling the temperature of a storage medium, allowing the saved energy to be used at any time. The TES system is crucial for balancing energy supply and demand. This system helps to store thermal energy from different sources such as solar, geothermal, and industrial waste heat, and it is also used for various applications such as power generation, water heating, building thermal comfort, battery thermal management, etc. [17]. TES guarantees a dynamic response to the local boundary conditions, granting high energy densities and a unique caching effect on indoor thermal fluctuation. The key characteristics of TES systems include capacity, charge and discharge rate, efficiency, storage duration, time of response, and cost.

With the growth of urban population and modern architectural designs, buildings have lost much of their natural thermal stability, increasing reliance on energy-intensive heating and cooling systems. The use of TES systems is a beneficial tool for the improvement of energy efficiency [18], increasing energy saving, shift heating and cooling loads, and the reduction in emissions [19]. There are three TES modes: sensible heat storage (SHS), latent heat storage (LHS), and thermochemical heat storage (THS) [20].

#### 1.2.1. Thermochemical Energy Storage (TCES)

Thermochemical energy storage (TCES) is characterized by high energy density, high exergetic efficiency, and the ability to operate at high temperatures. Energy is stored as chemical potential within the molecular bonds of the substances during the charging and discharging processes, and it released through reversible chemical reaction. TCES can be classified into two primary mechanisms: chemical reaction and sorption systems. Chemical energy conversion offers higher storage efficiency compared to sensible and latent heat storage, but identifying suitable reversible reactions for practical application remains challenging. Solid–gas thermochemical storage is often limited by poor heat and mass transfer and low cycle efficiency. Common reactions include hydration, carbonation, ammonia decomposition, metal oxidation, and sulfur cycles.

#### 1.2.2. Sensible Heat Storage (SHS)

SHS remains the most used method for storing thermal energy. It is the simplest and easiest form of heat storage technology due to its simple operation and reasonable cost. SHS involves storing energy by increasing the temperature of a material efficiently [21]. The quantity of energy stored relies on the material’s specific heat capacity, temperature change, and quantity. The SHS advantage relies on the fact that the storage and release of the accumulated heat (charging and discharging cycles) can be repeated, while including a large volume to meet the needs. The typical materials used are clay, brick, sandstone, wood, concrete, etc. Researchers are currently focusing on concrete enhanced with PCMs to decrease the energy requirements of buildings. Another area of contemporary material research involves the use of solid particles as a medium for sensible heat storage in Concentrated Solar Power plants.

#### 1.2.3. Latent Heat Storage (LHS)

The LHS uses thermal energy to induce phase change within a material that absorbs or releases heat during a phase transition at a nearly constant temperature. It is the most efficient method to store thermal energy [22,23]. LHS relies on materials called PCMs. The typical material used are water–salt solutions, water, paraffins, clathrates, salt hydrates, sugar alcohols, etc. Water represents the most prevalent PCM, historically utilized in the form of ice for thermal energy storage. Key criteria for selecting PCMs include melting enthalpy, temperature, and cost. PCMs require encapsulation for effective use, with microencapsulation being one of the most extensively researched techniques.

### 1.3. Phase Change Materials (PCM)

**Figure 1:**
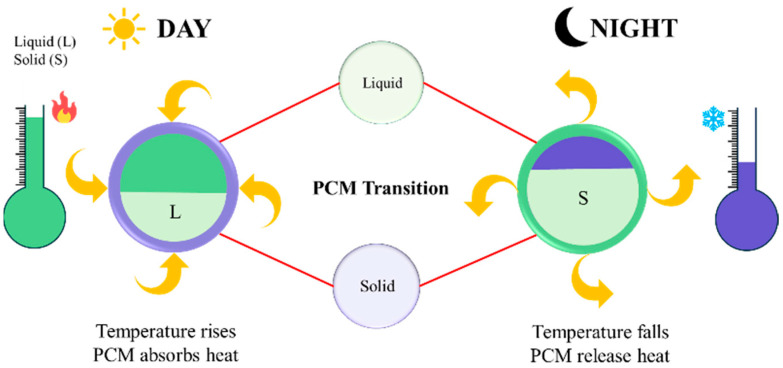
Phase transition of PCM.

Phase change materials (PCM) have gained attention as materials for storing thermal energy in LHS [24,25,26]. PCM can store and release latent heat (LH) during phase transitions, and it offers a higher energy storage density compared to SHS, as energy is stored without a significant temperature change [27]. PCMs regulate the temperature during the process of warming-up (load) and cooling-down (discharge). PCM absorbs heat from the surroundings during the day, and at night, as the temperature decreases, it solidifies, releasing the stored heat back into the environment. Figure 1 illustrates the phase transition phenomena of the PCM during the heating and cooling process.

PCMs should be properly selected, considering the final application. PCMs can be used in building walls, building components other than walls (such as floors and ceilings), and in heat and cold storage units located in building interiors instead of the envelope (such as heat/ice storage tanks) for heating and cooling. The PCM main conditions are listed in Table 1 [28,29] as function of the kinetic, thermophysical, chemical, economic, and environmental aspects. The selection process of an appropriate PCM for a particular application is complex due to the large number of PCMs available in the market or still being developed.

### 1.4. Types of PCM

PCMs can be categorized based on their phase transition temperatures: low-temperature PCMs, with a melting point < 220 °C, intermediate-temperature PCMs, with 220 °C ≤ melting point ≤ 420 °C, and high-temperature PCMs, with temperatures higher than 420 °C.

#### 1.4.1. Organic PCM (OPCM)

OPCMs mostly consist of carbon–hydrogen chains. OPCMs have numerous functional groups that provide useful LHS. OPCMs can be classified as paraffin and non-paraffin, i.e., fatty acid, esters, alcohol, and polyethylene glycol (PEG), although their usage is based on paraffin, fatty acid, and sugar alcohols. OPCMs are robust, secure, have a LH of fusion and a wide compatible range of temperature (15–45 °C), suitable for construction materials. OPCMs have thermal and chemical stability, non-reactivity, recyclability, are abundant, and commercially available at acceptable costs. Nevertheless, OPCMs can suffer leakage during melting transitions and have low thermal conductivity. After 1000 cycles of thermal cycling, it was found that OPCM has stability, although its melting characteristics and LH of fusion eventually changed [30]. Paraffin, fatty acids, polymeric materials, sugar alcohols, and polyalcohol are low-temperature PCMs. As most OPCMs have melting points < 80 °C, they are considered of low temperature.

#### 1.4.2. Inorganic PCM (IPCM)

IPCMs are usually applied in high-temperature solar applications [31]. They have a T_m_ range between −100 °C and 1000 °C. The IPCMs are salt hydrates, metals, and alloys. Salt hydrates and molten salts are mainly used for high-temperature range application. The advantages of IPCM rely on their high LHS, non-flammability, high thermal conductivity and operating temperature range, and low cost. The main IPCM disadvantage is corrosion, which reduces the encased material’s lifespan. They endure a phase segregation and supercooling, which limits their capacity to store thermal energy [32]. The inorganic–inorganic eutectic PCMs are categorized under the intermediate temperature. Nitrates, metal carbonates, sulphate, fluorides, and chlorides, among others, are included in the category of high-temperature PCMs and can be widely applied for the high-temperature TES requiring temperatures above 500 °C.

#### 1.4.3. Eutectic PCM (EPCM)

EPCMs are classified as uniform blends and mixtures of two or more organic–organic, inorganic–inorganic PCMs, and organic–inorganic PCMs. The EPCMs melt at a lower temperature than the melting point of the sole PCM. EPCMs show a single melting and freezing peak during the heat flow [33]. EPCMs have better TES performance than simple PCM systems. Some barriers that can be overcome by preparing EPCMs are supercooling and phase segregation. EPCMs have a high entropy change, melting leads to larger disorder, and a major modification occurs in the melting point. Some EPCM characteristics are phase stability, high LH of fusion, low thermal conductivity, and high cost [34].

Over the past 30 years, salt hydrates, paraffin waxes, fatty acids, and eutectic organic/inorganic compounds have been the most widespread PCMs [35]. Table 2 shows the advantages and disadvantages of the different types of PCM.

## 2. Nano, Macro, and Microencapsulation of PCM

PCM encapsulation has been extensively used in a range of industries in recent decades, including food storage, pharmaceuticals, construction, textiles, and solar energy [23]. The encapsulation technique consists of preventing PCM leakage, avoiding its contact with the environment, and enhancing the thermal properties. The PCM is in the inner part of a capsule, located in the core, and the capsule material is the shell [36]. PCM encapsulation increases heat transfer surface area, providing excellent thermal stability, and keeping the volume largely constant during cooling and heating cycles. There are different methodologies to encapsulated PCMs, which are mainly physical (spray drying, solvent evaporation) or physical–chemical (in situ, interfacial, emulsion, suspension) [37]. The shell can be made of organic materials (polymers such as melamine-formaldehyde, resins, etc.) or inorganic materials (mainly silica) [38,39,40].

Several factors have been examined in the PCM encapsulation process, including the shell material’s compatibility with the core PCM, the appropriate shell thickness to ensure the required diffusion tightness, the mechanical stress durability of the designed encapsulated PCM—which take place at the shell due to volume change—the capsules’ competent resistance to shear stresses that occur under various working conditions, and the reduction of the negative effects of encapsulation on subcooling, among others.

Based on the final diameter of the capsule [41], the encapsulation methodology of PCMs can be classified into PCM nanoencapsulation or NEPCM (<1 μm), PCM microencapsulation or MPCM (1 μm to 1 mm), and PCM macroencapsulation or ePCM (1 mm to >1 cm).

### 2.1. Nanoencapsulation of PCM (NEPCM)

A system in which PCM is encapsulated in nanometre-sized capsules (nm) is known as NEPCM [42]. When NEPCM is suspended in a fluid medium, the suspension can absorb and release heat at a constant fusion temperature. When TES is considered, the combination of NEPCM with nanofluids enables more controlled and efficient heat transfer, making it a key component in building heating and cooling systems. This integration demonstrates their usefulness in sustainable construction by increasing the speeds of mass and heat movement while reducing entropy [43]. Some studies have demonstrated that decreasing the capsule size greatly improves the performance of the PCM [44,45,46].

NEPCM materials are currently being used in engineering applications, from medicine delivery to TES in buildings [47,48]. Overall, NEPCM materials provide solid-to-liquid phase transition, a large number of energetic changes, stabilization of temperature, and variation in thermal conduction during phase transition as unique qualities listed below [49]. Nevertheless, the diversity and complexity of NEPCM use is still exceedingly challenging to categorize.

### 2.2. Macroencapsulation of PCM (ePCM)

Since PCM has a limited thermal conductivity, ePCM may restrict heat transport [50]. The selection of PCMs and shell materials for ePCM plays an important role in the development and efficient utilization of LHS systems in building envelopes [51,52].

The shells used for ePCM are classified into metal (stainless steel, copper, and aluminum) and non-metal materials, such as polyvinyl chloride (PVC), glass, silicon dioxide (SiO2), plastic, polyolefin, and high-density polyethylene (HDPE) [53]. The key criteria for the selection of shell materials in construction applications are the non-flammability and nontoxicity, suitability for encapsulated PCMs, thermal stability and mechanical strength, high heat conductivity, and accessibility and financial viability [54].

In recent years, extensive research has focused on incorporating ePCMs into building envelopes, including walls, floors, roofs, ceilings, and windows [55,56], where the indoor thermal comfort is enhanced, and the building’s energy use drastically lowered.

### 2.3. Microencapsulation of PCM (MPCM)

MPCMs have been successfully incorporated into textile, medicine, foams, slurry, electricity/solar and into building sector [57,58], such as cement, concrete, bricks, walls, and roofing systems, to enhance energy efficiency and optimize indoor comfort [59]. Numerous opportunities exist for incorporating MPCMs into building materials, including cement, mortar, concrete, paints, and other coatings and functionalized textiles [60]. The microencapsulation process has emerged as a promising technique for integrating PCM, offering enhanced thermal stability, prevention of material leakage, and improved overall efficiency in building applications [61,62]. However, despite their benefits, challenges like thermal degradation, high costs, and environmental impact still need to be addressed through further research and innovation.

Microencapsulation technology is suitable for avoiding PCM leakage and incorporating it into building materials, although the loss of mechanical qualities requires numerous new studies [35]. Furthermore, most of the polymers used as shells for MPCM are demonstrated to be durable after multiple cycles of heating and cooling, thus being beneficial for maintaining thermal comfort [41].

Several well-known polymerization techniques, such as interfacial polymerization, suspension polymerization, and emulsion polymerization, can be used to formulate MPCMs. The basic idea of all three methods is the development of a chemical process that quickly produces a thin, flexible polymeric coating on the surface of a liquid PCM bead [63].

### 2.4. Pros and Cons of Macro-, Micro-, and Nano-Based PCM

In TES systems, the stability and integration of PCMs are enhanced by the encapsulation process, which employs containers but has limited versatility and efficiency. Although MPCM improves stability and heat transfer, it is costly. The pros and cons of each encapsulation method for PCMs are listed in Table 3.

### 2.5. Polymers Used in MPCM Systems for Building Applications

Polymers play a fundamental role in the development and performance of PCM and MPCM systems for TES in buildings [64,65]. Their chemical versatility, low density, mechanical flexibility, and compatibility with construction materials make polymers ideal candidates for both PCM stabilizations and encapsulation [66]. In PCM-based building applications, polymers are primarily used as supporting matrices, shape-stabilizing frameworks, or encapsulation shells, enabling the safe and efficient integration of latent heat storage materials into building components [67].

Thermoplastic polymers like polyethylene (PE), polypropylene (PP), polystyrene (PS), and polymethylmethacrylate (PMMA) are particularly common because of their good thermal stability, mechanical strength, and chemical resistance [68,69]. They can physically trap the PCM within their polymer network through capillary action or molecular interactions, forming shape-stabilized PCM (SSPCM) composites that preserve structural form while still providing high latent heat storage. Epoxy resins and polyurethane (PUR)-based materials have also been studied for immobilizing PCM in rigid and semi-rigid building components, offering enhanced structural strength and long-term reliability under repeated thermal cycling [70]. PMMA and PS polymeric shells are generally regarded as more environmentally benign and can be prepared via a variety of polymerization methods, making attractive alternatives for MPCM applications [66,71]. These materials are chosen for their ability to create uniform, mechanically robust shells that effectively prevent PCM leakage and shield that core from chemical degradation. Polymer-based microcapsules also offer high surface area to volume ratios, improving heat transfer efficiency and enabling uniform dispersion within cementitious, gypsum-based, or polymeric building composites [72]. The polymeric shells are particularly widespread because of their processing flexibility and ability to form-stable MPCM. Melamine-formaldehyde (MF), urea-formaldehyde (UF), and poly(lactic acid) (PLA) provide favourable combinations of mechanical strength, thermal stability, and cost-effectiveness [73]. MF shells are often selected for their excellent thermal resistance and solvent stability. The use of formaldehyde-based resins has raised environmental and health concerns associated with potential formaldehyde emissions. In MPCM systems, polymers are often used as shape-stabilizing matrices to prevent leakage during the solid–liquid (S-L) phase change [71].

The utilization of polymers for MCPM applications has also grasped much consideration due to their thermal, mechanical, and environmental performance [74]. Some polymers are thermally stable within typical building operating temperatures, resist thermal fatigue over many phase change cycles, and are compatible with adjacent construction materials [75]. In addition, factors such as polymer permeability, shell thickness in MPCM systems, and interfacial adhesion play critical roles in determining the thermal behaviour and long-term durability of PCM-based composites [76]. The ongoing research advancements in the chemistry of polymers can tune the polymer structures and compositions which improve the PCM loading capacity, enhance thermal conductivity, and extend life cycle [77,78].

Recent work has increasingly turned to recycled and bio-based polymers for MPCM systems, driven by sustainability targets and circular economy concepts. Materials such as recycled polyethylene (rPE), recycled polypropylene (rPP), recycled polyethylene terephthalate (rPET), and recycled polystyrene (rPS) [79], whose shells are made from polymeric waste demonstrated chemical, physical, and mechanical properties that are equally excellent to those of commercial alternatives. They showed promising results as both supporting matrices and encapsulation shells, providing thermal stability on par with virgin polymers while lowering environmental impact. The use of waste-derived polymers in MPCM not only enhances the overall sustainability of TES technologies but also contributes to the development of low-carbon, energy-efficient building materials.

## 3. Life Cycle Assessment (LCA)

There is a concern about the release of micro and nanoplastics into the environment during the microcapsules’ manufacture, because the particles can be inhaled or ingested by humans, accumulating in the body and causing adverse effects such as inflammation, oxidative stress, and alterations. Polymeric materials used in encapsulation processes may contain additives or residual monomers that, when released, pose health risks [80]. Adequate safety precautions must be considered when producing and handling MPCMs and NEPCMs. This covers the selection of biocompatible and toxic-free materials, the use of personal protective equipment, and rigorous environmental controls. Furthermore, additional studies would be required to completely comprehend the long-term consequences of exposure to this substance, to create regulations that would guarantee the security of both consumers and employees.

However, there has never been a greater demand for an integrated environmental assessment tool, since environmental managers and decision-makers are being forced to consider products and services from cradle to grave due to this enlarged perspective and a growing awareness of sustainability. Life cycle assessment (LCA) was born out of this requirement. LCA is a standardized tool which offers a solid scientific foundation for environmental sustainability in business and government. LCA compares the environmental behaviour of different items in a system. LCA gives a realistic picture of possible environmental trade-offs and offers a thorough analysis of the environmental effects of changing or choosing a product or process [81]. The software that are often used for this assessment are SimaPro, Sphera/GaBi, and OpenLCA, among others. The key impact categories in LCA are global warming potential, ozone depletion potential, acidification potential, ecotoxicity potential, human toxicity potential, and energy demand, among others.

In the following Table 4, a summary of the advantages and disadvantages of different LCA software used is shown.

## 4. Simulation Software

Several simulation tools have been employed to analyze and model building energy performance with integrated NEPCM, MPCM, or ePCM systems. In recent years, researchers use DesignBuilder 7.3, EnergyPlus 9.4, TRNSYS 18, ANSYS Fluent 25.1, COMSOL Multiphysics^®^ 6.4, and Sphera® LCA for Experts/GaBi as simulation tools.

### 4.1. DesignBuilder 7.3

DesignBuilder 7.3 is a comprehensive building performance simulation platform that integrates 3D modelling with advanced analytical capabilities to facilitate sustainable design and research. Developed on EnergyPlus simulation engine, it enables the rapid generation of building while providing detailed assessments of energy consumption, carbon emissions, and thermal performance. The software incorporates the Radiance engine for daylighting analysis, allowing rigorous evaluation of illuminance distribution, glare potential, and control strategies. Its HVAC module offers a structured environment for modelling and simulating heating, cooling, and ventilation systems, including detailed control mechanisms and equipment performance. Furthermore, the inclusion of computational fluid dynamics (CFD) allows for the investigation of airflow patterns and thermal comfort within indoor environments. Additional tools for cost and life cycle analysis support economic and environmental assessment, while dedicated compliance modules streamline certification processes such as LEED and ASHRAE 90.1. Featuring a robust 3D interface and integrated analysis capabilities, DesignBuilder 7.3 serves as an intuitive platform that simplifies the complexities of EnergyPlus modelling. Despite its accessibility, the software’s disadvantages include significant commercial licencing fees, performance degradation when managing intricate geometries, and fundamental dependency on the EnergyPlus engine for its primary simulations.

### 4.2. EnergyPlus 9.4

EnergyPlus is a thermal load and energy analysis software developed to model and simulate the heating, cooling, ventilation, lighting, and other energy flows within the buildings. Some key simulation features are variable time steps, user-configurable modular systems coupled with a mass balance and heat-based zone simulation, thermal comfort assessment, multizone airflow modelling, and natural ventilation analysis. Both temperature-dependent thermal conductivity and phase change enthalpy are taken into consideration by the implicit conduction finite-difference algorithm used to create the PCM module in EnergyPlus. In addition, the EnergyPlus has become an important tool to be utilized on understanding how a building is executing energy wise. It enables the evaluation of various energy conservation measures, including insulation, HVAC system efficiency, renewable energy integration, and the use of advanced materials such as MPCM. Nevertheless, EnergyPlus is a free and open-source building energy simulation tool known for its high accuracy and strong HVAC modelling capabilities, making it popular in research and industry. However, it has a demanding learning curve, the absence of a built-in graphical user interface, and potentially extensive computational requirements for complex systems.

### 4.3. TRNSYS 18

TRNSYS 18 programme is a modular which allows the output of one type to act as the input of the other within the model [82]. These types of simulation have been broadly executed on modelling buildings, as well as complex systems. TRNSYS 18 also includes simulation studio (graphical user interface) and the ability to interface with external programmes like MATLAB, FLUENT, etc. [83].

MATLAB is a high-level programming language and interactive environment widely used for numerical computation, data analysis, algorithm development, and visualization [84]. Also, it provides powerful toolboxes for specialized applications such as signal processing, control systems, optimization, machine learning, and image processing. Then, ANSYS Fluent is a leading CFD software used to model fluid flow, heat transfer, turbulence, and chemical reactions within complex geometries [85]. Fluent is widely applied in engineering fields such as aerospace, automative, energy, and chemical processing, in which it supports the design and optimization of systems involving aerodynamics, combustion, ventilation or multiphase flows.

TRNSYS has been used to study PCMs for passive applications like floors, ceilings, and wallboards and evaluate their effect on energy savings and temperature regulation [86]. In addition, it has been used for active PCM systems, evaluating the HVAC systems. For the PCM wall model, TRNSYS can be used for PCM-enhanced building envelopes to enhance the performance of energy [87]. This model can also be employed for LHS systems to explore the energy storage efficiency in solar thermal systems or waste heat recovery systems. The performance of TRNSYS can be further advanced by coupling it with MATLAB, which enables the modern optimization of PCMs in terms of charging and discharging cycles. TRNSYS can also simulate the behaviour of MPCM in cementitious materials in applications like roads, pavements, and buildings [88]. This helps in the phase transition dynamics and thermal buffering effects to boost energy efficiency in buildings. The integration of TRNSYS with FLUENT enables the exploration of such materials under real conditions. Distinguished by its user-friendly interface and modular architecture, TRNSYS excels in custom component modelling for thermal systems; however, it faces drawbacks such as high licencing fees and less sophisticated building physics than EnergyPlus. It requires user experience to navigate its complex simulation environment.

### 4.4. ANSYS Fluent 25.1

ANSYS is a software for simulation in multiple applications. ANSYS has recently been broadly used for scrutinizing the thermal and mechanical performance of MPCM in building applications. It offers a widespread platform for modelling and simulating the phase transition dynamics, heat transfer, and structural integrity of MPCM-integrated in the building material [89]. The thermal response can be modelled to evaluate the heat absorption and release by MPCM, exploring the low thermal fluctuations and thermal insulation in a building. Furthermore, ANSYS can also simulate the MPCM mechanical stability under material degradation, repeated thermal cycles, and durability [90]. The ANSYS Multiphysics with fluent solver can be employed for the investigation of brick models, where the heat flux can be examined through the brick as well as the inner wall temperature [91]. It is a robust engineering tool for structural, fluid, and thermal Multiphysics simulations, featuring industry-leading solvers and meshing capabilities. Although superior for complex geometric modelling, the platform requires heavy hardware resources and specialized training. These advantages are further tempered by its high acquisition cost.

### 4.5. COMSOL Multiphysics^®^ 6.4

COMSOL Multiphysics^®^ is a simulation tool for MPCM modelling in building applications. This tool provides an innovative Multiphysics environment where thermal, structural, and fluid dynamics interactions can be simultaneously elaborated. The COMSOL model can be used to explore the TES performance and MPCM LH release. The COMSOL model can also simulate the solidification and melting process, from which the efficiency of the PCM can be extracted in different climatic conditions [92]. COMSOL Multiphysics^®^ examines the stress and strain behaviour of MPCMs in the building materials under thermal contraction or expansion, and models the cracks and deformation chances owing to the repeated phase transitions [93]. It provides a flexible simulation environment that facilitates the integration of several physical domains within a single model, supported by a user-friendly interface and use in academic research and early-stage development. The limitations include high licencing cost, scalability issues for large 3D models, and weaker industrial-grade CFD and structural performance than ANSYS.

### 4.6. Sphera ® LCA for Experts/GaBi 10.7

Sphera/GaBi software evaluates the LCA and is extensively utilized for the evaluation of the environmental impact of materials and processes throughout their lifespan. Sphera/GaBi software, in the MPCM field, applied in building applications helps in quantifying the energy savings and sustainability, and the environmental benefits of integrating PCM-based TES systems in building materials [94,95]. Meanwhile, the Sphera/GaBi is used for different applications of MPCMs, specially in buildings, to indicate the environment friendly nature of the encapsulated materials and to extract the energy consumption, CO_2_ emission, and other environmental impacts. For the optimization of MPCM integration in green building certifications like (BREEAM, LEED, etc.), the scenario model can be used [96]. With the Sphera/GaBi software, the energy savings potential for MPCMs in both active and passive TES systems can be evaluated [97] and can also reduce the energy demand for HVAC systems by simulating the effects of MPCM-enhanced building materials on heating and cooling loads [98].

### 4.7. SimaPro 10.3

SimaPro, a leading professional solution for LCA developed by PRé Sustainability, measures the environmental and socio-economic impacts of products. Its versatile platform supports eco-design initiatives, environmental labelling, and comprehensive carbon and water footprinting. It utilizes high-quality life cycle inventory (LCI) databases such as EcoInvent, Agri-footprint, and various industry-specific libraries to provide comprehensive background data on materials, energy, and transport. The software implements the ISO 14040 standard, supporting the full LCA process: from initial scoping and inventory analysis to advanced impact assessment features such as characterization and normalization. SimaPro licence types are for Classroom, faculty, PhD, Analyst, and developer. Also, the SimaPro Developer licence has other features like the ability to insert external links, import scenarios from Excel (only available in SimaPro), edit expression, move parameter, and convert to constants. SimaPro categorizes methods into six groups, such as: Europen, Global, North American, Single issue, Water footprint, and Superseded.

### 4.8. OpenLCA 2.6.0

OpenLCA 2.6.0 is a free open-source and high-perfomance software widely used for LCA and sustainability modelling. In addition to environmental LCA, it supports life cycle costing, Social LCA, and footprint analyses (carbon and water). OpenLCA complies with ISO 14040/14044 standards and enables the integration of leading databases and over 40 impact assessment methods, including ReCiPe 2016, CML-IA, and IPCC 2021. It features advanced analytical tools for Monte Carlo uncertainty simulation, Sankey diagrams, and regionalized impact assessment, and provides researchers and industry professionals with a transparent, reproducible, and cross-platform environment for complex environmental modelling without the barrier of licencing costs.

### 4.9. One Click LCA (https://oneclicklca.com/, accessed 5 February 2026)

One Click LCA is the world’s leading patform for decarbonizing the built environment. By automating life cycle assessments and embodied carbon tracking, it empowers architects, engineers, and manufacturers to hit net-zero target with ease. With a massive database of 500,000 verified datasets and seamless integration with 20 BIM tools, it transforms intricate carbon data into high-integrity, compliant reports at industry-leading speeds.

## 5. Applications of MPCM in Buildings

Different MPCMs have been examined in passive systems, including concrete [99], mortar [100], bricks [101], roof [102], windows [103], paints [104], gypsum [105], walls [106], as well as in active systems such as cold and heat storage devices [107,108]. In addition to paraffin-based MPCMs, growing attention has been directed toward natural and bio-based MPCMs, especially those based on fatty acids, vegetable oils, and natural waxes, due to their renewable nature and lower environmental burden. For researchers in this field, it is important to add MPCM to increase the thermal inertia of the above-mentioned matrices, improving their capacity to absorb, store, and release heat [109,110].

### 5.1. Cement and MPCM

The cementitious material has demonstrated that with the incorporation of MPCMs there is a notable advantage in enhancing thermal performance and minimizing thermal stresses during the hydration process [111]. MPCMs have good phase change properties and can be used as potential materials for indoor temperature regulation and energy saving in building envelopes [112]. The exploration of MPCM incorporation into cement-based systems is a step in the right direction for enhancing the functionality and environmental impact of traditional construction materials. Owing to these advantages, the recent advancement in this field has been expanded from simple thermal regulation toward a more sophisticated multifunctional cement composite, which leads to a broad spectrum of approaches ranging from passive temperature control to systems with dynamic optical or electromagnetic behaviour.

Building temperature management, heat storage, solar energy use, and other functions are all facilitated by the addition of MPCM to cement-based material. These characteristics make MPCM technologies the current target of research and an opportunity for future development in the field of building energy conservation [112]. Consequently, a few studies have investigated how MPCM integration can be tailored to address specific challenges such as thermal inertia, electromagnetic pollution, urban overheating, and design optimization.

**Figure 2:**
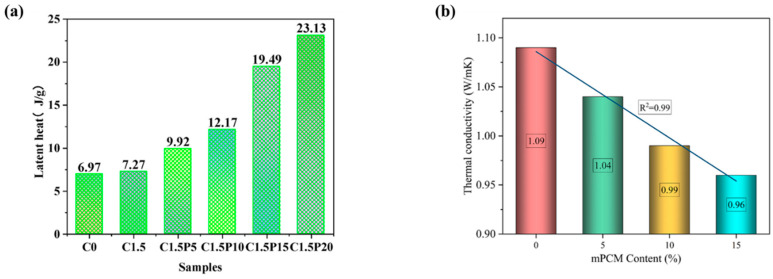
(**a**) Latent heat of pure and composite cements [114]; (**b**) thermal conductivity performance after 28 days with increasing concentration of MPCM [119]. Reproduced with permissions.

The use of MPCM in cement-based materials has gained attention for enhancing the thermal performance of building materials. The external environment, MPCM characteristics, and the interaction between MPCM and the cement matrix all have a significant impact on the service performance of cement-based thermal storage materials. Through heat storage and release at the phase transition point, MPCMs have been shown to control and stabilize interior temperature [112]. The improvement in thermal performance has motivated many researchers for integrating MPCMs into cementitious matrices. These efforts span a spectrum from fundamental passive solutions, primarily focused on regulating surface temperature, to multi-functional systems that incorporate combined optical and structural properties. It is important to emphasize that Urban Heat Island (UHI) effect is a primary driver of elevated surface and surrounding air temperatures, leading to extreme heat events. Consequently, the UHI negatively correlates with increased energy demand for cooling buildings, diminished indoor thermal comfort, and potential adverse public health outcomes. Therefore, a novel cementitious plaster made of Ordinary Portland cement type I, lime, and sand as aggregate was developed to mitigate the UHI effect and cast into tiles measuring 10 cm × 10 cm with a thickness of 1.2 cm and a base weight 275 g. The plaster was integrated with MPCM (Nextek^®^ (Microtek Laboratories, Dayton, OH, USA), $T_{m}$: 18 °C, 24 °C, 28 °C) and thermochromic (TC) paint (leuco dye, TT: 31 °C, blue/red) by Soudian et al. [113]. These components were added at a 2.5% mass fraction each, resulting in 6.9 g of MPCM and 1.4 g of TC pigments in the combined samples. Two main types of samples studied: first, MPCM and TC paint integrated within the cement plaster base; and second, the MPCM was only integrated within the plaster, and the TC paint covered the surface of the tile. The second type samples (TC on surface) exhibited 23% higher solar reflectance at 45 °C (hot cycle) compared to the base plaster, while retaining beneficial solar absorption in the cold cycle (8 °C). The lower thermal conductivity due to MPCM inclusion, combined with the dynamic optical properties, indicates the plaster’s potential for effective, year-round facade performance. Therefore, this development has been promising because the samples have the potential to reduce solar reflectance at high temperatures and could absorb solar radiation at lower temperatures. However, both Soudian et al. [113] and Xie et al. [114] highlight a recurring challenge: MPCM-containing systems often suffer from low thermal conductivity or dispersion issues, which reduce thermal response efficiency. This limitation has motivated studies focusing on improving heat transfer and distribution within cement matrices. For instance, Xie et al. [114] developed a dual-functional cement composite to solve electromagnetic radiation pollution and enhance thermal comfort. They incorporated Carbon nanotubes (CNT) and MPCMs into P-O 42.5 Ordinary Portland cement. The optimal composite (C1–5P20) used 18 g CNT and 240 g MPCM per 1200 g cement and 1800 g sand, achieving a 20 mm thickness. The MPCM significantly improved impedance matching and broadened the effective electromagnetic wave absorption bandwidth (RL < −7 dB). The addition of 20% MPCM reduced the heating rate by ~47%, demonstrating enhanced heat storage capacity and thermal inertia. As the MPCM content increases, it also increases the heat energy storage density of the cement material which indicates that a phase change reaction occurs within the composite, leading to enhance its TES performance as displayed in Figure 2a. Despite observable strength loss due to increased porosity, the composite’s mechanical strength remains acceptable for non-bearing applications like plastering mortars. These materials are positioned as an inventive, sustainable, and high-value solution for building technologies because it provides definite benefits in terms of energy efficiency and environmental impact reduction.

The study by Erkizia et al. [115] investigates innovative, potentially active, cement-based TES systems using MPCMs and reduced graphene oxide (rGO). The core problem is the poor thermal conductivity and lack of active control in traditional MPCM-cement systems. They compare two paraffinic MPCMs (MRT24, Nx24) and one bio-based natural MPCM (MPT25) at 20% volume with 0.3 wt% rGO (based on binder weight). Nx24 showed the best heat storage capacity (19 J·g^−1^) and lowest hysteresis. However, rGO addition failed to significantly improve thermal/electrical conductivity due to poor dispersion. The conclusion stresses the importance of full material characterization and formulation optimization for successful TES applications.

Another recurring challenge in MPCM-cement composites is the tendency of microcapsules to agglomerate, which reduces both their thermal efficiency and the mechanical integrity of the material. To address these challenges, Gu et al. [116] explored whether MPCMs can be used in foam-stabilized cement to improve energy storage and thermal insulation. The tendency of MPCM to agglomerate affects its dispersion and lowers its efficiency, being a significant obstacle when integrating MPCM into cement-based product. To tackle this issue, the investigation introduces a nanoparticle-stabilized foam system that enhances the interaction of viscosity modifiers, surfactants, and foam stability. According to the results, adding 15 vol% of MPCM reduced the thermal conductivity of foam cement from 0.11 W·m^−1^·K^−1^ to 0.07 W·m^−1^·K^−1^. Hence, this MPCM/foam composite cement material significantly improves the cement’s heat storage capacity and reinforces its insulation properties. While Gu et al. [116] improved dispersion and thermal insulation, other studies focused on maximizing LH capacity by engineering MPCM shells and increasing allowable loading fractions. Zhao et al. [117] performed suspension polymerization to create MPCMs consisting of an n-octadecane core and a styrene–divinylbenzene copolymer shell. The results show that the sample MPCM with a 2:1 shell-to-core ratio offers the best thermal stability and highest latent heat storage, with a melting enthalpy and encapsulation efficiency measured at 111.5 J·g^−1^ ± 0.7 J·g^−1^ and 51.4 ± 0.7%. In addition, four distinct building boards were made with different weight percentages of MPCM (0, 10, 20, or 30%) in the cement matrix. The board with 30 wt% MPCM has a 67.82% higher heat energy storage capacity than the traditional construction material in the typical temperature range of 10 to 50 °C. The board with 30 wt% MPCM shows significant thermal regulation and insulation properties and is a viable approach to promote building energy saving.

Furthermore, Bre et al. [118] studied the use of a multi-objective computational method that dynamically couples the NSGA-II optimizer with the EnergyPlus simulation software to optimize cementitious panels enhanced with MPCM to reduce annual heating and cooling loads. The problem is optimizing passive MPCM design, which is complex due to the contradictory performance of heating and cooling loads. The core material used is a generic cementitious composite based on an experimental mixture with a water/cement (w/c) ratio of 0.4. The panel material used 20% volume fraction of Micronal^®^ DS 5038 X MPCM (paraffin wax, T_peak_ = 24.5 °C), supplied by Microtek Laboratories, Dayton, OH, USA. However, optimizing all variables showed that the best balance (max total saving 12.09%) was achieved with maximum thermal conductivity (10 W·m^−1^·K^−1^), T_peak_ = 26.24 °C, and 0.35 m thickness. This work highlights that the increasing panel thickness is the only effective with high thermal conductivity to guarantee thermal coupling with indoor air. Therefore, the optimization at the building-system scale is important, as the MPCM performance depends strongly on melting point, panel thickness, and thermal conductivity in real operating conditions.

To improve TES and lighting efficiency in buildings, Gencel et al. [119] explored the creation of a light-transmitting glass fibre-reinforced cementitious composite incorporating 15 wt% of Nextek 18D MPCM (paraffin, T_m_ ~ 18 °C, LH = 200 J·g^−1^), supplied by Microtek Laboratories, Dayton, OH, USA, as MPCM. The composite incorporates MPCM, calcined kaolin (15% of the binder), AR-GF (0.5% by volume), and polymethylmethacrylate optical grids (Litracon pXL^®^). By increasing the MPCM concentration, there is a decrease observed in the thermal conductivity of all the samples; i.e., it was 1.09 W·m^−1^·K^−1^ and reduced to 0.96 W·m^−1^·K^−1^, as shown in Figure 2b. The optimal M15-LTCC slab achieved up to 12.4% artificial light transmittance. Thermoregulation test showed that the M15-LTCC provided a cooler room temperature for ~6.5 h when the temperature was above 21–23 °C and reduced thermal conductivity from 1.09 W·m^−1^·K^−1^ to 0.96 W·m^−1^·K^−1^. The structural adequacy is assessed through compliance with ASTM C109 and ASTM C348 standards, confirming suitability for architectural elements such as façade panels. Although MPCM addition reduces mechanical strength, the achieved values (25–40 MPa) remain well above the limits for non-load-bearing applications, maintaining structural integrity and serviceability under long-term use. Despite a 28% reduction in compressive strength due to low density material and voids from damaged MPCM, the material provides significant dual energy-saving benefits. The material can lower energy usage and promote greener building designs by integrating natural daylighting with MPCM’s TES. These results demonstrate the promise of cementitious composites with MPCM integration for sustainable and energy-efficient building application. Table 5 summarize some studies regarding MPCM, showcasing the main applications in building applications.

**Table 5:** Cement application in buildings using different MPCMs.

Authors	MPCM	Conditions	Thermal Performance Study	Observations	Ref.
Soudian et al. (2020)	Three OMPCMs: Nextek 18, Nextek 24, Nextek 28 (Microtek Labs).	Along with thermochromic paint, MPCMs are integrated into cement plaster.	Cement plaster combined with thermochromic pigments and PCM.	Applying TC paint increased the solar reflectance of cement plaster by 23%. Combining TC paint with MPCM enhanced solar absorption at lower temperatures compared to conventional plaster, indicating year-round performance benefits due to dynamically varying reflectance and absorptance.	[113]
Xie et al. (2023)	MPCM (Shanghai Xinwu Textile Technology Co., Ltd., China).	Ordinary Portland cement with MPCM.	Dual functional cement composites with carbon nanotubes and MPCM.	Demonstrated that cement composites incorporating CNT and MPCM exhibit enhanced electromagnetic wave absorption and improved TES. CNT optimize impedance matching to broaden the absorption bandwidth, while MPCM reduce the heating rate by about 47% to 20%.	[114]
Erkizia et al. (2024)	Paraffinic MPCMs: RT24 and Nextek 24D (Nx24), bio-based PCM: PureTemp25 (PT25).	RT24 and PT25: The encapsulation shell is made of melamine-formaldehyde (MF) and is produced via polymerization followed by spray-drying. Nextek 24D (Nx24): The shell is also based on melamine-formaldehyde but is crosslinked with a cyclic urea and a multifunctional aldehyde.	The thermal characteristics of cement-based materials that contained MPCM, both with and without rGO added to improve electrical and thermal conductivity.	Nx24: demonstrated the best thermal performance in terms of LHS and low hysteresis between heating and cooling peaks. MPT25: Good thermal performance but with larger hysteresis, which can affect its efficiency in TES applications. Adding rGO does not significantly enhance the thermal or electrical conductivity of the cement’s pastes, especially because of the poor dispersion of the rGO particles.	[115]
Gu et al. (2023)	Paraffin-based PCM encapsulated in silica shells.	Interfacial polymerization method.	The addition of 15 vol% MPCM reduced the thermal conductivity of foam cement from 0.11 W·m^−1^·K^−1^ to 0.07 W·m^−1^·K^−1^ at a dry density of 600 kg·m^−3^	Due to nanoparticle-stabilized foam improving the stability of the foam in the cement paste, restraining rapid defoaming and collapse. However, the conventional foam demonstrated duster rupture and instability. Also, the combination of foam and MPCM improved the thermal insulation properties of the concrete.	[116]
Zhao et al. (2023)	n-Octadecane.	MPCM with Styrene–divinyl benzene shell.	Evaluation of the thermal behaviour of cement matrix boards incorporating PCM.	The optimal MPCM (2:1 shell-to-core ratio) demonstrated high thermal stability with melting enthalpy of 111.5 J·g^−1^ and increased degradation temperature (160.5 °C). Heat storage capacity increased by 67.82% and temperature variations decreased by 59% when added to cement boards (30 wt% MPCM), improving insulation and thermal regulation. These findings demonstrate MPCM’s potential as energy-efficient construction materials.	[117]
Bre et al. (2022)	Commercial paraffin wax, Micronal**^®^** DS 5038 X, by BASF	MPCM with a powder-like form	Lower thermal conductivity can limit the effectiveness of MPCM, as it reduces the thermal connection between the MPCM and the indoor air.	It was observed that there was a conflict between the optimal design for heating and cooling. Therefore, it is better to have a thick panel with lower T_m_ for heating, meanwhile, for cooling, a thin panel with higher T_m_.	[118]
Gencel et al. (2022)	MPCM (Nextek 18D Microtek Laboratories, Inc.)	Encapsulating the branched-chain hydrocarbon mixtures as PCM is a melamine-formaldehyde shell.	MPCM-containing light-transmitting cementitious composite (LTCC)	The incorporation of the MPCM into glass fibre-reinforced cementitious composites improves thermal regulation and light transmittance and maintains stable indoor temperatures for 6.5 h. Meanwhile compressive strength decreases by 28% flexural strength remains stable, ensuring structural viability.	[119]
Salihi et al. (2025)	Paraffin wax (Grade RT-26, Rubitherm Technologies, Berlin, Germany).	CaCO_3_ shell. The microencapsulation process involved a self-assembly method.	The CaCO_3_ shell enhanced the thermal stability of the MPCM, delaying the decomposition temperature of the PCM by 12 °C. The MPCMs were stable within the temperature range relevant to building applications (<100 °C).	The MPCM mortar exhibited a thermal time lag (delay between peak outer and inner surface temperatures) of approximately 17 min. The MPCM mortar reduced the maximum indoor temperature by an average of 1.6 °C compared to the reference mortar.	[120]
Kumar et al. (2023)	Capric Acid (CA)	Form-stable PCM (FSPCM) Composite	Two mortar panels (20 × 20 × 2 cm^3^) were prepared, one with FSPCM (12 wt%) and one without (reference mortar). Tested under simulated summer conditions (15–35 °C).	FSPCM offers the highest thermal inertia (1.32 °C) and heat storage (690 kJ·m^−3^) but has the lowest compressive strength (3.66 MPa). One sample provided a balanced performance with moderate compressive strength (8.23 MPa), thermal inertia (1.2 °C), and heat storage (645 kJ·m^−3^), making it the best choice for building envelope applications.	[121]

### 5.2. Mortar and MPCM

The incorporation of PCM into cementitious mortar enhances its thermal storage capacity by exploiting LH during phase transitions, thereby moderating indoor temperature fluctuations and reducing building energy demand. However, direct PCM addition often causes leakage, weakens the cementitious matrix, and disturbs hydration. MPCM overcomes these limitations by enclosing the PCM within a polymeric shell, ensuring chemical stability, preventing leakage, and enabling uniform dispersion in the mortar. Mortars containing MPCM provide effective passive thermal regulation while maintaining acceptable mechanical performance, making them promising materials for energy-efficient building envelopes. Salihi et al. [120] developed CaCO_3_-shelled MPCM with paraffin wax (RT-26) cores demonstrating a 340% increase in thermal conductivity and a reduction in indoor peak temperatures by 1.6 °C under semi-acid climatic conditions. The mortar was produced using grade Ordinary Portland cement (CimarPro 45**^®^**, Ciments du Maroc Société Anonyme, Casablanca, Morocco) and river sand as fine aggregate, with particles sizes between 1.00 and 0.125 mm. These mortars maintained compressive strengths above 5.2 MPa, indicating that thermal performance can be significantly enhanced without compromising basic structural requirements. The structural adequacy was compared with the ASTM C270-10 with a minimum compressive strength of 5.2 MPa for type N mortars. Due to the addition of MPCM their strength reduces but it remains above the threshold value which is 7.78 MPa. Also, the CaCO_3_ shell helps their long-term functional reliability, which makes them more suitable for non-load-bearing applications such as interior plastering. Similarly, Gbekou et al. [122] explored bio-based liquid (natural) MPCM dispersion (ME29D) in Ordinary Portland Cement containing 99% clinker (EXTREMAT**^®^** CEM I 52, 5N-SR3 SEG, Vicat SA, L’Isle-d’Abeau, France) and sulfo-aluminous cement mortars (Alpenat, CSA). While MPCM incorporation led to a 64% reduction in compressive strength, minimum structural integrity (>17.2 MPa) was preserved. The structural performance is judged by whether compressive strength remains above the functional limits required for building envelope systems, even after a strength loss of 64%. Mortars were prepared following EN 196-1 and tested according to ASTM C109. Even though strength decreased from 47.9 MPa to 16.9 MPa, it remains above EN 998-1 limits, confirming suitability for non-structural applications. Nevertheless, adding MPCM greatly enhances thermal performance by improving TES and lowering thermal conductivity by 29% at 10.5 vol. More importantly, the inclusion of MPCM improved thermal performance, increasing TES and reducing thermal conductivity by up to 29%, demonstrating a favourable balance between energy efficiency and mechanical performance. Based on these findings, Gbekou et al. [123] have explored the thermal behaviour of cement mortar (denoted M15D) incorporating bio-based MPCM (CrodaTherm™ ME29D), aiming to solve the problem of high energy consumption and CO_2_ emissions in building sector by enhancing thermal efficiency. The cement mortar comprises sand, Ordinary Porland cement (EXTREMAT**^®^** CEM I 52.5 N, a fast-setting CSA, and a superplasticizer. The researchers approached this by conducting multiscale experimental tests on wall samples using a bio-climatic chamber and performing numerical simulation (with COMSOL and EnergyPlus) validated against experimental data, then scaling up the analysis to a building model. The most relevant results showed that incorporating the MPCM can lead to energy saving of up to 33% for heating and 31% for cooling in specific climate conditions, while also significantly influencing interior temperature and increasing thermal inertia. It is concluded that integrating bio-based MPCM into cement mortar (M15D) effectively enhances thermal performance and energy efficiency of building envelopes, especially in Mediterranean and degraded oceanic climate. These studies reveal some key trends which are the choice of MPCM shell and core affects thermal performance and structural stability; MPCM content and particle size determine heat storage efficiency and mechanical impact and combining multiscale testing with simulations is essential for practical building applications across climates. MPCM-modified mortars provide a flexible and expandable method of passive thermal control in buildings, successfully raising TES, lowering energy consumption, and preserving structural integrity. Table 6 explains the concept of mortars that have been utilized for building applications using MPCM.

### 5.3. Concrete and MPCM

Concrete is essential in construction, yet its limited thermal storage capacity restricts its role in improving building energy performance. PCM enhances concrete by absorbing and releasing heat during phase transitions, helping stabilize indoor temperatures and reduce energy demand. As mentioned previously, it is needed to add MPCM to maintain mechanical integrity and ensure compatibility with cement hydration instead of PCM to avoid leakage and weaken the matrix. MPCM-modified concrete offers improved thermal storage, more adaptable thermal conductivity, and contributes to lower heating and cooling loads.

The field of MPCM integration within cementitious composites is advancing through a dual strategy, as evidenced by recent research: Primarily, by engineering form-stable PCM (FSPCM) systems which rely on porous structures to contain the MPCM and prevent leakage. Later, by embedding MPCM directly into concrete, which enhances their durability without effecting their long-term thermal efficiency. These combined methodological improvements expand the scope of design for high-performance thermal building envelopes.

**Figure 3:**
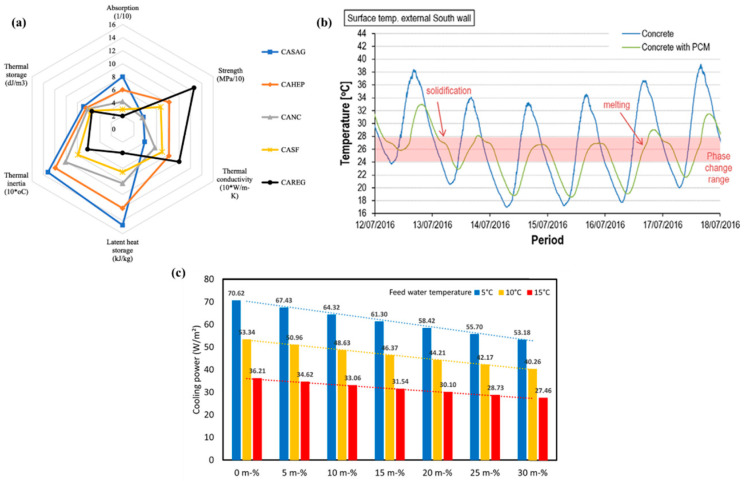
(**a**) Six parameters of the FSPCM [121]; (**b**) external surface temperature of both the cubicles [124]. The red labels and icons identify the phase change processes (melting and solidification) and active heat source; (**c**) wall module cooling power against different concentration of PCM [125]. Reproduced with permissions.

Kumar et al. [121] contribute by selecting suitable porous materials for developing FSPCM integrated into concrete panels to solve the problem of high building energy consumption and improve thermal comfort. Five porous materials were evaluated: silica aerogel granules, hydrophobic expanded perlite (HEP), recycled expanded glass, nano-clay, and silica fume. The PCM used was Capric acid (CA), a natural fatty-acid. The FSPCM concrete panels meet AS 3600 structural requirements, surpassing the 20 MPa minimum with compressive strengths of 26.3–31.2 MPa despite strength reduction from PCM inclusion. Long-term performance is supported by leakage stability test at 60 °C, confirming sustained chemical and mechanical integrity. Capric acid/hydrophobic expanded perlite panel provided the best overall performance, demonstrating a suitable compromise with moderate strength (8.2 MPa) and high thermal inertia (1.2 °C) thermal storage. Figure 3a shows the comparative analysis of the six key parameters of the FSPCM, those being absorption capacity, thermal storage, thermal inertia, LHS, thermal conductivity, and mechanical strength. The conclusion recommends HEP as an ideal porous material for integrating polar CA as a PCM into building envelopes.

While Kumar et al. [121] focuses on the material selection of form-stable systems, Lajimi et al. [126] extend the analysis to predict the behaviour of MPCM-enhanced concrete at the building scale. A numerical investigation of a ceiling system that includes microencapsulated phase change concrete (PCC) was performed. The MPCM used was paraffin RT25 (T_m_ = 26.6 °C) into the concrete. The purpose of the investigation is to know how to predict the effect of incorporating a layer of MPCM on thermal behaviour, humidity control, energy consumption, and occupant comfort under real climate conditions in Tunisia. DIGITAL Visual FORTRAN 95 software was used to model heat and mass transfer phenomena to analyze performance for both summer and winter conditions. The most relevant results showed that the PCC layer reduced cooling load energy consumption by 35% in July and lowered the total energy cost by 20%. Hence, the study confirms that PCC is an effective material for improving building energy efficiency, especially in hot climates like Tunisia. The research bridges the gap between material-level design (as in Kumar et al. [121]) and whole-building dynamic performance, showing how MPCM benefits persist beyond controlled laboratory conditions.

Complementing both short-term material optimization and numerical prediction, Cabeza et al. [124] explore a major gap in the literature: long-term durability. They evaluated the thermal performance of two house-like cubicles built in 2005. One cubicle had ordinary concrete; however, the other had 5 wt% of MPCM (Micronal DS 5001, T_m_ = 26.6 °C) incorporated to the concrete matrix. Both cubicles were made of preformed concrete panels. The external surface temperature of both cubicles was observed quite differently, as shown in Figure 3b. It was concluded that the cubicle with MPCM has the same thermal response obtained in 2005; no degradation was observed. Moreover, the MPCM in concrete is a long-lasting and durable technology for enhancing building energy efficiency. Evidence supports the practical viability and stability of MPCM as a building-integrated thermal storage solution providing the missing real-world validation that complements both the material innovations (Kumar et al. [121]) and the modelling predictions (Lajimi et al. [126]). The review emphasizes that PCM/MPCM-enhanced concrete effectiveness depends on the material engineering, building-scale simulation, and long-term stability in a synergistic manner, which is a promising approach to reduce building energy consumption. Table 7 shows some studies regarding the use of concrete integrated in buildings with MPCM.

### 5.4. Walls and MPCM/PCM

The integration of PCM and MPCM into building walls has been extensively investigated through both numerical modelling and experimental validation, revealing consistent benefits in thermal performance across diverse climates. A significant portion of the literature relies on computational tools, particularly EnergyPlus, COMSOL Multiphysics, and custom finite-volume solvers to predict transient thermal behaviour and guide optimal PCM/MPCM design building envelopes.

The EnergyPlus software has been broadly employed to investigate the MPCM in multiple research areas [127]. Salihi et al. [128] compared the outcomes of Alam et al. [129] with those simulated with EnergyPlus and showed good agreement with the theoretical results [128,129]. Early efforts to validate MPCM behaviour in dynamic conditions demonstrated the importance of ensuring accurate simulation inputs. For example, Zhuang et al. [130] successfully validated the MPCM algorithm using two different envelopes (A and B), comprising one layer and two layers of PCM, respectively. The study portrayed the high relative error in temperature for envelope A was 12.41% and 8.33% for envelope B. The findings highlighted the importance of utilizing accurate real weather data and thermal properties to minimize discrepancies between simulation and experimental outcomes. The experimental data of Kuznik et al. [131] has been confirmed by the theoretical analysis of Chan et al. [132] and Campbell et al. [133] using EnergyPlus programme. The theoretical results for the indoor air temperature have perfectly agreed with the experimental outcomes. This validation confirmed the accuracy of the PCM algorithm and reliability integrated into EnergyPlus, offering assurance in its application for analyzing the thermal performance of building envelopes incorporating MPCM concrete bricks.

In Mediterranean climates, Dardouri et al. [134] examined PCM-integrated external walls composed of bricks, cement, and plaster. Owing to these conditions, the building shows up to a 41.6% reduction in the energy demand with a lower T_M_ up to 21 °C, which favours heating energy savings. A thorough review emphasizes the need of climate-specific MPCM selection, confirming that precise MPCM properties and climatic inputs are crucial for dependable performance forecasts, as shown by EnergyPlus validation studies.

Based on these validated frameworks, several studies focused on implementing MPCM in wall assemblies. Xue et al. [125] evaluated the thermal performance of a modular radiant cooling wall system incorporating MPCM. This study was executed by using both experimental testing and numerical simulation. COMSOL Multiphysics was used to create a 2D heat transfer model to numerically explore the temperature fluctuation of the wall module. By using both experimental testing and numerical simulations, it was found that feed water temperature was the dominant factor influencing cooling power and radiant surface temperature. A 30% MPCM content extends discharge time by 2.37 times at 5 °C, enhancing thermal storage, but higher concentrations reduce cooling power due to lower thermal conductivity. It also displays the cooling power of the wall module at different water temperatures, ranging from 53 to 70 W·m^−2^ (5 °C), 40–53 W·m^−2^ (10 °C), and 27–36 W·m^−2^ (15 °C), as portrayed in Figure 3c. These insights help optimize MPCM-based radiant cooling systems for building retrofits and industrial use and also demonstrate the trade-off between thermal storage and conductivity that defines MPCM optimization in wall assemblies. Similarly, Babaharra et al. [135] conducted a comprehensive numerical assessment of multilayer walls with MPCM in hot Moroccan climates using a 2D finite-volume, enthalpy-porosity approach. Also, different configurations for walls were also studied. Integrating MPCM into an eight-hole hollow brick reduces heat flux by 32% and delays heat transfer by 3 h. Polyethylene spheres are used to encapsulate PCM, preventing leaking into the wall during its liquid phase. The coexistence of liquid and solid phases enhances amplitude attenuation and phase shift. It was confirmed that n-octadecane paraffin (T_m_ = 28 °C) provides the best internal thermal comfort during summer in the city of Khouribga.

Across the literature, a clear pattern emerges; microencapsulation effectively mitigates leakage and low thermal conductivity, enabling MPCM-enhanced walls to consistently lower peak indoor temperatures, reduce heat flux, and delay heat transfer. With rising demand for low-carbon and passive thermal regulation, MPCM-integrated wall systems offer a scalable, robust solution for boosting building energy performance across diverse climates. The applications of walls in buildings are elucidated in Table 8 by using different MPCM and PCM.

### 5.5. Roof and MPCM/PCM

Roof systems are central to passive TES, as they receive the highest solar exposure and thus drive much of a building’s heat gains and losses. Consequently, PCM- and MPCM-based strategies have proven effective for stabilizing indoor temperatures and improving energy performance, especially in extreme climates. By absorbing heat during the day and releasing it at night, PCM integrated in roofing systems such as concrete tiles, metal panels, or insulation layers can substantially reduce both cooling and heating energy demand.

Extensive research on PCM-based roofing systems has sought to clarify how temperature-dependent material properties affect building performance across different climate zones. Dardouri et al. [134] also explored the performance of PCM in a Mediterranean region to study their feasibility for energy consumptions. These PCM were integrated in the external or internal south roofs of the buildings under fours climate situations in Tunisia and then an EnergyPlus simulation software was used to scrutinize the thermal analysis of the building. The infinite R^TM^ PCM family was studied with different T_M_, and they are capable of being used as commercial energy storage applications. Optimized PCM configurations, particularly a 40 mm double-layer system can reduce energy demand by up to 41.6% and cut CO_2_ emissions by 38.74%. These results confirm that well-designed PCM systems substantially improve energy efficiency in Mediterranean buildings. Building on this climate-specific optimization approach, Chen et al. [136] explored the optimization of PCM parameters and the combined use of PCM and Cool Paint (CP) to solve the problem of high operational energy consumption in the building sector. EnergyPlus is used for numerical simulation across eight cities representing different climates, analyzing how PCM thickness (10 mm to 10 mm) and phase transition temperature (20 °C, 30 °C, 40 °C) affected energy demand and peak load. The PCM material properties were based on a SSPCM with a LH of 180 kJ·kg^−1^. At the end, it was found that for cities with low heating energy ratio, CP + PCM was superior to PCM alone. The conclusion is that PCM and CP must be strategically optimized and selected based on a building’s overall heating/cooling energy consumption characteristics to maximize energy savings. While, Dardouri et al. [134] highlighted the need to tailor T_M_ and layer configuration for Mediterranean climates, showing that these adjustments deliver reliable benefits in both heating and cooling-dominated conditions. Chen et al. [136] concentrated on optimizing interactions between PCM and coating layers.

**Figure 4:**
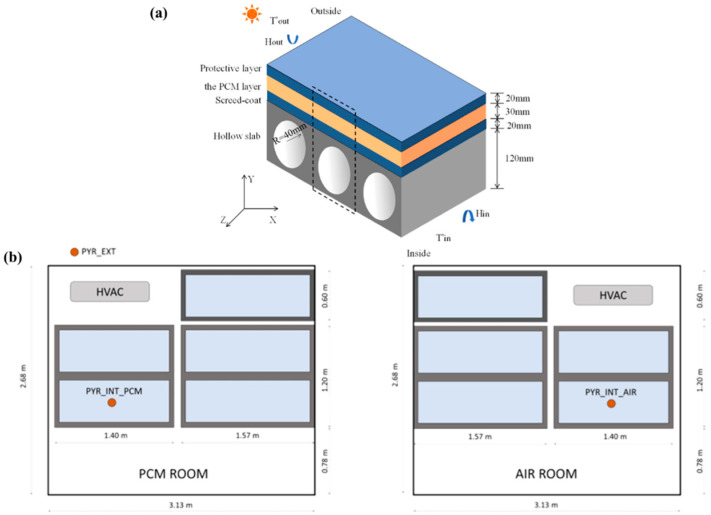
(**a**) PCM ventilation roof [137]; (**b**) schematic of windows in each room [138]. Reproduced with permissions.

The roof is considered as one of the weakest parts of buildings, owing to the intense solar radiation during summer weather. Therefore, it is essential to enhance the thermal properties of the roof and to reduce the consumption of energy for air conditioning. Yu et al. [137] reported the thermal performance of a new SSPCM for a new ventilation roof in Wuhan (China) by employing a dynamic heat transfer method; the structure of the phase change ventilation roof is shown in Figure 4a. A dynamic heat transfer model combining resistance–capacity and Number of Transfer Units methods was developed and applied in TRNSYS for seasonal simulations. The SSPCM made of 85% paraffin and 15% HDPE has a LH of 160.7 kJ·kg^−1^ and a melting range of 34–36 °C. Using a 30 mm SSPCM layer together with night ventilation at 3 m/s reduced the total summer cooling load by 37.5% compared to standard roof. This combination demonstrates strong energy-saving potential and serves as an effective low-carbon strategy for building in hot climates.

In addition, Luo et al. [139] computed the heat transfer efficiency of roofs that are filled with paraffin PCM by using the multiple relaxation time lattice Boltzmann technique. The study was conducted in five different cities. It was observed that the porous bricks roofs result in excellent operational properties in all the cities while using paraffin wax. One of the cities, Urumqi, where diurnal temperature variation is high, the system lowered roof heat flux by 69.8% and reduced the frequency of thermal discomfort to 7.4% at 28 °C far more effectively than in Shanghai, which has a smaller temperature swing.

The results show that pairing PCM with night ventilation can greatly enhance thermal comfort and energy saving, particularly when the PCM’s operating temperature is matched to the local diurnal cycle. Their findings connect directly with the conclusion of Yu et al. [137]; both studies emphasize that the match between PCM operating temperature and the local diurnal thermal cycle is essential for maximizing efficiency.

Beyond traditional on-site construction, Jia et al. [140] reported about PCM in multiple areas to prefabricate buildings and explore their influence on building energy consumption and indoor air temperature. While deliberating about the building energy consumption, which was obtained by using EnergyPlus, the indoor air temperature range was set at 18~24 °C based on ASHRAE comfort standard. The specific optimal PCM identified was cetane (with a phase transition temperature of 18 °C). The result indicates that PCM significantly improves thermal performance, with the highest energy saving in hot and mild climates (e.g., 77.11% in Kunming compared to 17.7% in Anda). This demonstrates the scalability and transferability of PCM roof solutions, while reinforcing the recurring insight from previous authors that PCM design must be geographically tailored. Moreover, evidence shows that, regardless of the climate region, PCMs perform better when they are placed on the interior of walls and roofs.

Studies across various climates consistently show that PCM-integrated roofs reduce peak indoor temperatures, slow heat transfer, and improve thermal comfort. This research has evolved from material-level optimization (Chen et al. [136], Dardouri et al. [134]) to hybrid roof designs (Yu et al. [137]), climate-specific thermal modelling (Luo et al. [139]), and prefabricated, industrialized systems (Jia et al. [140]). With the growing demand for sustainable construction, PCM-based roofing offers and effective passive strategy to lower dependence on mechanical cooling while minimizing environmental impact. The utilization of roof into buildings using various MPCM/PCM and encapsulated materials is compiled in Table 9.

### 5.6. Windows and PCM

The integration of MPCM in windows like double glazing, laminated glass, and frames helps regulate the temperature inside, minimizing heat transfer, and reducing energy consumption. The efficiency can be improved when it is combined with low-emissivity or photochromic glass, making these an essential solution for sustainable design and nearly zero-energy buildings.

Research on MPCM-integrated windows has moved from early investigations on liquid paraffin-filled glazing units to more advanced solid–solid (S-S) and functional PCM systems, each tackling specific limitations of the previous generation. The thermal performance of the PCM-filled with double-glazing window in building was studied by Uribe et al. [138], as can be seen in Figure 4b. They explored the energy performance and solar radiation transmission of PCM glazing to solve the problem of high energy consumption (especially for cooling) in office buildings with glazed facades in semi-arid climates. A year-long real-scale experiment compared a reference office room with double-clear glazing window filled with air (identified as AIR) and another equipped with a double clear glazed window filled with PCM in Santiago, Chile. Paraffin RT25, with PCM near 25 °C, reduced transmitted solar radiation during melting, yielding energy savings of 22% for cooling and 45% for heating in key seasonal weeks. The PCM glazing effectively enhances thermal inertia and lowers energy demand in highly glazed buildings with large temperature swings. Real-scale experiments are essential because they provide the closest possible representation of how a material, technology, or system behaves in real conditions.

Based on direct light transmission, Heim et al. [141] analyzed the melting kinetics of Paraffin RT 21HC PCM filled inside one chambre of a triple glazing window. The melting behaviour of the PCM within the glass unit proved to be complex, involving interreflections and refraction of light across semi-layers exhibiting different physical states: solid, liquid, and mushy. The results indicate that modelling light transmittance as a linear function of temperature is inaccurate, highlighting the need for a detailed understanding of the optical effects in the mushy state to improve model development. Also, the fundamental insights provide knowledge on optical behaviour in the mushy state, which is a key requirement for developing realistic and accurate simulations. To address the challenges of leakage and low-temperature applicability, recent research has shifted away from liquid paraffins toward S-S PCM systems. Ma et al. [142] proposed an innovative glazing window was designed with S-S PCM and silica aerogel aiming to minimize energy demand in buildings, especially in the severe cold regions of China where PCM struggles. To optimize materials and aerogel thickness, thermal simulations in EnergyPlus and daylighting analysis in Radiance were made possible using an equivalent window model. The PCM used was a modified poly-based S-S PCM and the optimal silica aerogel thickness was found to be 10 mm. An equivalent glazing window with S-S PCM and silica aerogel integration (SPGW) model was developed for annual EnergyPlus simulation, overcoming PCM limitations, and combined with daylighting analysis in Radiance. SPGW achieved up to 18.2% energy savings over single glazing at 10 mm, showing S-S PCM with silica aerogel boosts efficiency in cold climates while maintaining daylighting standards. Similarly, Gao et al. [143] worked on understanding the thermal performance of S-S translucent PCM (SS-PCM) that was applied to a transparent glazing window for buildings. A validated equivalent thermal model simulated translucent PEG-based S-S PCM windows (3 mm, 250 kJ·kg^−1^) in EnergyPlus across three climates. Results showed up to 17.2% HVAC energy savings in warm climates, with solar absorptance as the most sensitive parameter, highlighting S-S PCM windows as an energy-efficient technology with peak load shifting benefits.

Functional PCM windows can dynamically change their optical characteristics. To create a smart window that can change its transparency in response to temperature, Wijesana et al. [144] utilized SSPCM composite based on chitin nanofiber (CNF) and PEG. The PCM used was PEG, stabilized by CNF isolated from crab shells. It is shown that the optimized device became transparent (88%, transmittance) above the melting point, exhibiting mechanical stability and successfully addressing leakage issue. CNF offer a promising approach to L-S SSPCM, enabling new “smart” window application. The thermal performance of PCM-embedded shutters for building windows in two Mexican cities, Merida (warm weather) and Toluca (cold weather), was assessed by Chen-Pan et al. [145] using numerical simulation studies. The two PCMs tested were Paraffin wax MG29 and n-octadecane. MG29 presented reductions in the inner surface heat flux by 71.6% and maintained the comfort temperature of 16.23 h. Moreover, Roy et al. [146] developed a low-cost solution-processed smart window composite coating using paraffin PCM, PMMA, and photosensitive metal oxide (In_2_O_3_/ZnO). COMSOL Multiphysics simulation was used to corroborate experimental thermal observation. The optimal composite film IZPC-5 (5 wt% In_2_O_3_/ZnO) maintained an average indoor temperature of ~28 °C and ~55 °C outdoors and showed switchable wettability. In general, the studies show a clear evolution: liquid PCM offers thermal buffering, S-S PCM adds stability in cold climates, and functional composites introduce optical control. Such advancements pave the way for PCM-integrated glazing, where microencapsulation boosts durability, prevents leakage, and enhances optical behaviour driving the next novel generation of PCM window systems. In addition, the application of windows has been summarized for buildings using PCM and encapsulation of different materials, as displayed in Table 10.

### 5.7. Bricks and MPCM/PCM

**Figure 5:**
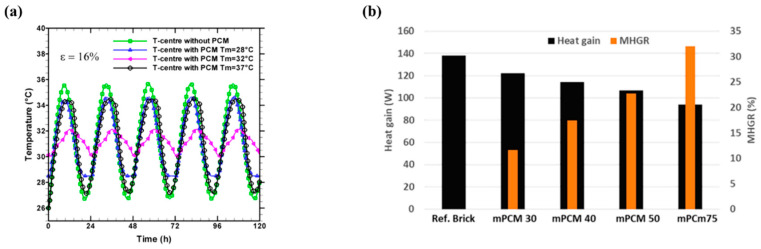
(**a**) Variation in T_m_ with different PCM [147]; (**b**) heat and maximum heat gain reduction [151]. Reproduced with permissions.

The buildings frequently undergo large thermal variations, increasing dependency on heating and cooling systems. The incorporation of MPCM into bricks provides LHS; this prevents heat transfer and stabilizes indoor settings by absorbing and releasing thermal energy during phase transitions. A major obstacle when using encapsulation technology on bricks is making sure that it is as durable as possible to stop leaks and degradation over time. By applying mechanical strength and heat storage capacity, bio-based, salt hydrate, or paraffin-based MPCM can be incorporated into porous, composite bricks, or lightweight materials. Therefore, Mahdaoui et al. [147] has reported a simulation-based study on the integration of n-nonadecane MPCM in building hollow bricks (12 hole) that are widely used in Morocco construction with the aim of improving the thermal performance of external walls using the software ANSYS Fluent 25.1. The MPCM was integrated as cylindrical capsules within the brick’s solid matrix. The variation in temperature with different PCM melting as displayed in Figure 5a. Adding 16% MPCM stabilized the inner surface at ~27.5 °C and effectively dampened heat waves. The current research illustrates that increasing the amount of MPCM into the brick has a positive effect related to the thermal performance of the brick wall, also it is important for this new material to evaluate mechanical, economic, and environmental aspect. On the other hand, Dabiri et al. [148] conducted a thermal analysis of a brick incorporating MPCM and ten air cavities, designed for application in building envelope systems. The main objective of their study was to minimize heat transfer between outdoor and indoor environments by analyzing both latent and sensible heat storage provided by the MPCM. The PCM used was RT35 contained in a steel capsule at the brick’s centreline. Moreover, CFD simulations were employed to investigate the behaviour of the MPCM under time-dependent external conditions. MPCM bricks regulated indoor temperature, using mainly LH in summer and sensible heat in winter, reducing summer temperature fluctuation by 48.5%. Therefore, the investigation demonstrates that LH is not the only method through which thermal energy can be stored; hence, SHTES is also appropriate for thermal storage. In addition, Al-Yasiri et al. [149] perform an experimental study that consist of the thermal performance of MPCM incorporated in fabricated bricks (three with MPCM and one without PCM) under Iraq’s hot weather conditions. The PCM used to prepare the PCM capsules was a paraffin wax with good thermophysical properties. Under the highest outside temperatures, it was discovered that MPCM with high T_M_ can greatly enhance the thermal performance of bricks. As a result, the brick containing 5 PCM capsules (442 cm^3^) has the best peak temperature reduction (PTR) of 156.5% and a conductive heat transfer reduction of ~61%, mainly because it had a larger capsule heat transfer area of (320 cm^2^) compared with the reference. Also, the best time delay obtained for the brick which contains 5 PCM capsules (442 cm^3^) was a 133% increment in the peak time-shift. Experimental confirmation complements the previous CFD-based studies by proving that capsule surface area and T_m_ selection critically determine real-world thermal inertia, especially in extreme climates. Moreover, Aakash et al. [150] evaluated the effectiveness of PCM (no microcapsules) in brick masonry walls for managing cooling loads in residential buildings. A paraffin-based RT PCM with 240 kJ·kg^−1^ LH and variable melting points was used. While the results show that PCMs do not reduce total daily heat gain or cooling loads, they effectively dampen hourly fluctuations, making them suitable for peak load management. For the proper operation of the PCM, it is recommended to put it in the inner side of the wall with enough insulation shielding it from outdoor conditions, as well as having a melting point that is near the indoor set-point temperature. Also, it demonstrates that performance depends not only in PCM properties but also on insulating strategies and thermal set-point alignment. A novel cement brick model filled with MPCM was directly filled into the hollow cavities of the brick, which was reported by Mukram et al. [151]. Four different MPCMs were tested by shifting the MPCM-filled holes at 30 mm, 40 mm, 50 mm, and 75 mm from the outer wall. ANSYS Fluent software was used for CFD to analyze the thermal performance of MPCM-filled cement bricks and for the verification of experimental testing in a psychrometric chamber, replicating real day and night climate conditions. A commercially available MPCM (MEP29) was employed and it demonstrated that MPCM-filled cement bricks enhance passive cooling by reducing heat flux and indoor temperature, and MPCM75 brick shows the highest energy saving, with a 32% reduction in heat gain and 1.2 °C below the indoor temperature, as displayed in Figure 5b [151]. Also, Fraine et al. [152] investigated whether expanded polystyrene (EPS) can be replaced with a new phase change humidity control material (PCHCM) for hygrothermal insulation in building construction. Yet, PCHCM is prepared by incorporating MPCM and diatomite, which is then filled in a sintered hollow brick that is typically used in Algerian building construction. The study compared the effect of EPS, diatomite, and PCHCM on the comfort level of the building indoor environment. With the first case study, it was demonstrated that the optimal location of PCHCM is given when it is filled with 66% in the internal side of the hollow brick. However, it was found that MPCM/diatomite has the potential to save energy, reaching 50% compared to EPS; additionally, it can be used as a hygrothermal insulation material in building construction.

Furthermore, research has demonstrated that MPCM-enhanced bricks can reduce inside peak temperatures, promote thermal inertia, and contribute to energy saving, which makes them very useful in areas with extreme climate conditions. The implementation of MPCM in bricks offers an alternative to conventional building materials as sustainable construction and low-energy building design obtain global importance. The employment of bricks in buildings is tabulated in Table 11 for MPCMs/PCMs and encapsulated materials.

### 5.8. Paints and MPCM/PCM

One of the most popular and effective approaches is surface coating techniques because they are adaptable methods for altering the wetting properties of building materials. However, the application of protective coating can considerably impact the durability, performance, and longevity of the materials in humid and adverse conditions. So, the application of paints and impregnating agents creates a protective barrier on the material’s surface, safeguarding it from water infiltration and other external elements. These coatings, used in painting with diverse functionalities, provide hydrophobicity, photocatalytic, and antibacterial activity, etc.

**Figure 6:**
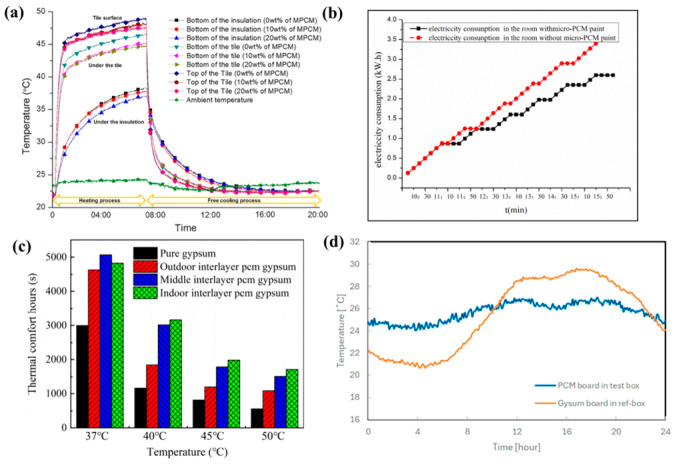
(**a**) The dynamic heat transfer for heat storage [158]; (**b**) the consumption of electricity between rooms [159]; (**c**) thermal comfort of phase change gypsum board [160]; (**d**) surface temperature on the PCM board in test box and gypsum board [161]. Reproduced with permissions.

Researchers can efficiently control and reduce temperature variation in interior spaces by adding PCM to paint compositions. This innovative method not only increases paints usefulness but also fits well with the growing demand for sustainable construction methods. As part of the development of intelligent and environmentally friendly building solutions, this study investigates the incorporation of PCM into paints, evaluating the possibilities of adaptive coatings that improve thermal efficiency while preserving aesthetic appeal [153,154,155]. The research reflects a change from traditional protective coating toward multifunctional thermal-regulating surfaces, in which paint becomes an active contributor to energy efficiency. Furthermore, Ghayedhosseini et al. [156] investigated the individual and combined effects of incorporating Cool Paint (CP) and PCM into the building envelope of high-rise office buildings on overall energy demand. EnergyPlus simulation evaluated three PCM, including a white CP combined with ATP23 PCM. When placed in the inner layer of external walls, this pairing lowered annual energy demand by 10.11 kWh·m^−2^·year. The decrease in the gas and electricity demand was observed to be about 6.49% and 1.29%, respectively. The findings indicate that coupling CP for seasonal gains with PCM for continuous performance effectively enhances energy efficiency in high-rise buildings. Qin et al. [157] investigation focused on creating bifunctional paint that pairs daytime radiative cooling with LHS to overcome heat-gain limitations. As a result of their high enthalpy of 71.35 J·g^−1^ and melting range of 28–34 °C, octadecane-in-silica microcapsules were inserted in an acrylic matrix. When compared to conventional radiative coating, the paint produced a 2 °C temperature drop and a definite buffering effect. All things considered, incorporating LHS shows promise for increasing the efficiency of passive cooling solutions. However, this study still had limitations because the temperature was still going up after the phase transition because of the shortage of PCM and UV light absorption and broad IR, which can be improved by innovative preparations of composite with high uptake properties of PCM, etc. Meanwhile, Jeong et al. [158] addressed the growing need for UHI mitigation by the development and thermal performance of a heat storage paint reinforced with MPCM. The trend of dynamic heat transfer for heat storage as shown in Figure 6a. MPCM-paint blends were developed and examined through Scanning electron microscopy (SEM), Differential scanning calorimetry (DSC), and complementary tests, then applied to roof tiles to gauge their thermal behaviour. The formulation used a paraffin-cored MPCM dispersed in an acrylic emulsion paint. The coating lowered peak tile temperature by 1.1 °C and extended heat-delay by 40 min, demonstrating the energy efficiency potential of MPCM thermal-storage paints. Their innovation links material-scale thermal storage with real-world application, bridging the gap between laboratory formulations and practical implementation.

Naikwadi et al. [162] developed a TES decorative paint by incorporating MPCM. The n-Nonadecane PCM is encapsulated using an oil-in-water emulsion method within a PMMA-co-BA-co-MAA copolymer shell. The MPCM 1 emulsion was prepared with a 1:1 core-to-shell ratio and subsequently blended with a PMMA-co-BA-co-MAA acrylic emulsion binder. These blends were then incorporated into the mill base at concentrations of 50 wt% (A1-MPCM1) and 60 wt% (A2-MPCM1). The DSC analysis of MPCM1, A1-MPCM1 (50 wt% MPCM1 in paint), and A2-MPCM1 (60 wt% MPCM1 in paints) showed melting enthalpies of 138.88 J·g^−1^, 35.37 J·g^−1^, and 41.23 J·g^−1^, and crystallization enthalpies of 137.21 J·g^−1^, 33.89 J·g^−1^, and 41.72 J·g^−1^, respectively. So, MPCM-based paints effectively store and release thermal energy, reducing temperature variations in buildings, the energy efficiency enhanced in reducing cooling energy demand and making it a sustainable solution for passive thermal regulation. Likewise, TES efficiency increased with 50 wt% and 60 wt% loading in outdoor building paint. For construction use, Han et al. [159] explored the effect of a new MPCM coating on indoor temperature response and energy consumption to solve the problem of mitigating temperature fluctuation and reducing energy use in buildings. MPCM produced vi ins situ interfacial polymerization with an OPCM core and PMMA shell were mixed 1:1 with standard paint and applied to interior walls for side-by-side room tests. They also demonstrated a relatively high LH of 139.68 J·g^−1^ and the trend of electricity consumptions are portrayed in Figure 6b. Furthermore, the coating reduced temperature amplitude by 2.8 °C and cut summer energy by 15.3%, confirming its effectiveness as a passive thermal-stabilization and energy-saving strategy. So, higher encapsulation efficiency means more PCM per microcapsule, leading to greater LH and improved heat storage and temperature control. The prepared microcapsules achieved 70–85% efficiency, significantly enhancing thermal performance. Their non-adhesive behaviour further improves encapsulation and the effectiveness of the core material, offers a simple yet innovative approach to PCM design.

The experiments under discussion demonstrate how PCM and MPCM can help regulate temperature by being combined with paint, brick, gypsum, and cement. These materials show promise in reducing temperature swings by efficiently utilizing PCM’s thermal characteristics, particularly during periods of high heating or cooling. This aspect supports the main objective of maximizing building energy use. To create building surfaces that are both thermally responsive and energy efficient, it is recommended that future studies and applications in the sector consider and investigate the incorporation of PCM into paints. The applications of paints using different MPCMs/PCMs for buildings are summarized in Table 12.

### 5.9. Gypsum Boards and MPCM

As a flexible and popular material in the building sector, gypsum offers potential as a matrix for incorporating PCM to reduce temperature swings and improve efficiency. The creation of advanced materials that can dynamically regulate temperature in reaction to external factors is supported by this method, which also enhances the thermal performance of gypsum-based materials. In response to the changing needs of modern construction methods, the incorporation of PCM into gypsum represents a step forward toward intelligent and sustainable building solutions.

Several studies have explored the use of gypsum boards integrated with MPCM for enhancing building energy efficiency. Xu et al. [163] investigated the regulation of the temperature fluctuations and energy consumption by using a commercial gypsum board with MPCM called Conmfortboard23 in summer. They also developed a numerical simulation approach to predict the thermal performance and energy saving potential of MPCM-incorporated gypsum boards (Comfortboard23 from Knauf), in which they used a CFD or energy simulation software such as EnergyPlus 9.4 or TRNSYS 18. The result was that the Comfort-board23 exhibited a 2% reduction in thermal transmittance, enhancing thermal insulation. Using MPCM increased the heat storage capacity by about 45% because of its LHS capabilities. Due to the outcomes, Comfortboard23 might result in significant energy savings, possibly reducing heating and cooling energy usage by 10% to 20% while improving thermal comfort and enabling the potential downsizing of HVAC systems. For the same application, Wadee et al. [164] studied the development of a novel gypsum plaster composed of high energy storage PCM loaded granules for energy efficiency in buildings. They used software called DesignBuilder 7.3 to predict the thermal performance of the gypsum plaster, which has PCM-loaded granules incorporated, focusing on calculating the potential energy savings in a domestic building. The introduction of PCM granules to cement block resulted in the largest energy reduction of 293.8 kWh. However, PCM gypsum plasters provided the highest level of thermal comfort. Complementarily, Kumar et al. [165] also investigated gypsum board integrated with PCM for improving TES; SSPCM-0, SSPCM-5, SSPCM-10, and regular gypsum powder (G-0, G-SSPCM, G-SSPCM-5, and GSSPCM-10) were used to create four distinct gypsum boards. These gypsum boards were tested for their impact on the building’s indoor thermal behaviour by incorporating them into small cubicles on the roof and south wall. Compared to G-0, GSSPCM displayed 3.0 °C for the roof and 4.54 °C for the south wall. G-SSPCM-5 recorded a maximum time delay of 200 min for the roof on day one, whereas G-SSPCM-10 recorded a maximum time delay of 180 min for the south wall.

Further experimental and analytical research has deepened understanding of MPCM-gypsum composites. Nowak et al. [166] measured a maximum volumetric heat capacity of 7.15 MJ·m^−3^·K^−1^ for MPCM gypsum boards, surpassing earlier work by Shukla et al. [167]. Gao et al. [160] reinforced that MPCM placement is pivotal; boards with interior MPCM layers exhibit the most effective performance in maintaining thermal comfort.

By incorporating MPCM into the gypsum board, the building’s interior temperature profile has improved. Nowak et al. [166] evaluated the thermal performance of MPCM-integrated gypsum plasterboard using experimental and analytical methods; aiming to characterize the enthalpy curve and hysteresis of the composite material and assess its energy storage potential for building applications. The gypsum board incorporating MPCM exhibited a maximum specific heat capacity of 8772 J·kg^−1^·K^−1^, equivalent to a volumetric heat capacity of 7.15 MJ·m^−3^·K^−1^. These results surpass those reported by Shukla et al. [167], who observed a value of approximately 6 MJ·m^−3^·K^−1^ fora gypsum board containing 25 wt% paraffin-based PCM. Furthermore, the heat capacity values recorded during the melting and solidification phases differed, revealing the presence of thermal hysteresis within the material. In this regard, Gao et al. [160] focused on the use of MPCM in gypsum boards to enhance their performance in building applications. The study demonstrates that incorporating stabilized MPCM into gypsum boards improves their thermal performance, extends indoor thermal comfort, and has potential application in energy-efficient buildings as can be seen in Figure 6c. Also, the positioning of the MPCM layer plays a critical role, with the interior side placement offering the best performance.

Building upon these foundations, MPCM-gypsum studies have elevated this approach toward multifunctional, durable systems. Bake et al. [168] characterized gypsum plasterboard integrated with MPCM (Micronal**^®^** DS5040X PCM powder) for energy-efficient buildings through thermal/physical property measurement. The result shows that the MPCM-gypsum plasterboard also lasts longer than gypsum plasterboard and has a higher operating temperature of around 1.5 °C, especially during the discharging phase. Because MPCM was added, the MPCM-plaster had 0.4 W·min^−1^ more stored energy than gypsum plasterboard. Similar findings were reported by Errebai et al. [169], disconfirming the idea that by increasing the mass percentage of MPCM in gypsum, it will always lead to an increase in heat absorption. Based only on volumetric heat capacity (steady state), the results demonstrate that an increase in the MPCM mass percentage in gypsum improves heat storage. Moreover, it is advised to consider only 20% of the mass percentage of MPCM because this produces an output that is extremely like 30% of the mass percentage of MPCM and produces an ideal value for thermal effusivity. Likewise, Bravo et al. [170] evaluated the thermal performance of gypsum boards enhanced with MPCM in a cubic test enclosure, considering the climatic conditions of Santiago de Chile during September-November period of 2017. They developed a numerical and experimental investigation which shows that the numerical behaviour is similar to those recorded experimentally. The numerical results were created by using EnergyPlus to carry out a thermal simulation. Although the numerical results indicate that the enclosure should not be manufactured by minimizing infiltrations, the amount of infiltration should be a parameter to be measured or controlled in future experiments.

Expanding the material’s functionality, Gencel et al. [171] fabricated a novel energy storage light-transmitting gypsum composite for thermal and lighting energy efficiencies in structural/architectural applications. Because microcapsules in general have low density, adding 10 and 15 wt% MPCM decreased compressive strength by 17% and 40%. Compliance with ASTM C472 and C348 confirms the structural adequacy of the MPCM gypsum, with compressive strengths of 5.4–10.3 MPa exceeding standard requirements. Glass fibres and stable microcapsules maintain ductility and prevent PCM leakage, preserving and thermal performance. According to the DSC study, MPCM-LTGC15 improves thermal storage by melting at 17.76 °C with a LH of 19.2 J·g^−1^. The suggested light-transmitting gypsum composite, which contained 15 wt% MPCM, maintained the room warmer when the temperature fell below the melting-freezing point, while providing a cooler temperature during peak temperatures.

In colder climates, Zhou et al. [161] demonstrated that MPCM-gypsum panels maintain improved temperature regulation even under subarctic conditions, highlighting the adaptability of these materials. The findings demonstrate that the incorporation of MPCM into gypsum leads to notable modifications in its thermal behaviour. The behaviour of surface temperature on both the PCM board in test box and gypsum board is shown in Figure 6d.

Collectively, these studies delineate a clear research trajectory from PCM-gypsum systems focused primarily on thermal control to MCPM composites offering multifunctional and climate-responsive performances. While thermal efficiency improvements are well-established, future advancements must address mechanical stability, long-term durability, hysteresis behaviour, and economic feasibility. The integration of advanced encapsulation technologies, adaptive simulation tools, and life cycle analysis will enable the next generation of intelligent, high-performance gypsum-based composites tailored for sustainable and low-carbon building. Notably, it provides a first-of-its-kind analysis of the internal working mechanics of MPCM-integrated gypsum, addressing a significant gap in the current literature compared to extensively studied cementitious composites. Although MCPM-enhanced concrete has been widely studied, the internal mechanisms and structural response of gypsum incorporating MPCMs remain largely unexplored. These outcomes comprehensively examine the fundamental mechanics of gypsum-MPCM systems, providing new insight into their coupled structural and thermal behaviour. The application of gypsum board in buildings using different encapsulated materials and MPCM/PCM are depicted in Table 13.

### 5.10. Insulating Materials and MPCM

The need for more energy-efficient buildings has driven research into cutting-edge insulation materials that can reduce energy use and improve thermal efficiency. The capacity of MPCM to store and release LH during phase transitions has made it a potential option among these. By incorporating these materials into building envelopes, it becomes possible to achieve superior thermal regulation and reduce dependency on mechanical heating and cooling systems, ultimately leading to better indoor comfort and energy savings.

**Figure 7:**
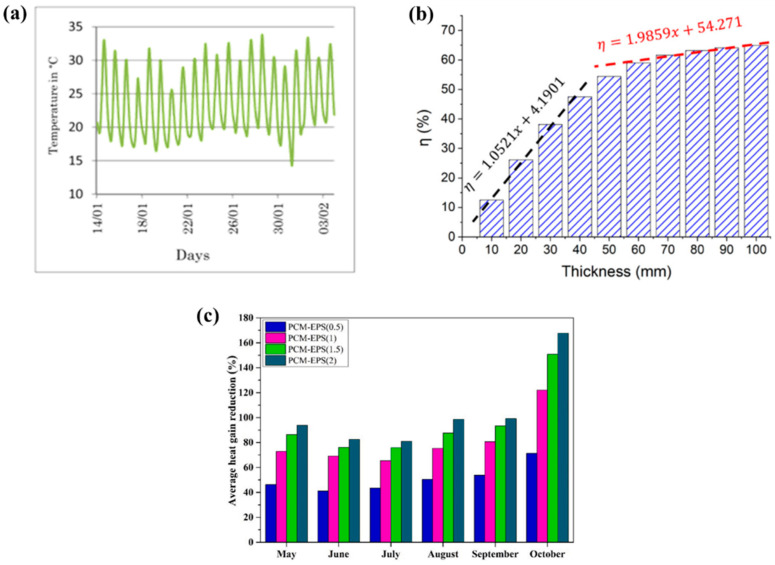
(**a**) Simulation profile of indoor temperature [172]; (**b**) optimum thickness of PCM in envelope of building [173]; (**c**) average heat gain reduction in rooms [174]. Reproduced with permissions.

Hamooleh et al. [172] analyzed and investigated how to improve thermal insulation and bio-PCM in buildings to increase occupant comfort and energy efficiency. For this reason, the study employed the response surface method and EnergyPlus simulations. The investigation evaluates various bio-PCM and insulation combinations in four different climates in Iranian cities. The simulation profile of indoor temperature can be seen in Figure 7a. PUR insulation and BioPCMDSCM27Q21 were found to be the most effective materials because they improve energy efficiency and thermal comfort. The optimal insulation and PCM thicknesses range from 6.9 cm to 9.8 cm and 5 cm, respectively, for all cities except Tehran. Through optimization, the thermal comfort improved by 25–60%, heating electricity decreased by 43–99%, and cooling electricity consumption was reduced by 38–52%. Also, the optimization of thermostat setting and material selection reduce energy consumption while maintaining comfort.

Similarly, Arumugam et al. [175] aimed to explore the effects of insulation, BioPCM and natural ventilation on indoor temperatures and energy consumption by using DesignBuilder software under Chennai’s climatic conditions. They tested four building models with different BioPCM and insulation configurations to determine the most effective approach for maintaining comfortable indoor temperature. The PCM used are the following BioPCM**^®^**Q27 and BioPCM**^®^**Q29 and the insulation material was extruded polystyrene–HFC blowing. The results show that BioPCM combined with insulation enhances thermal comfort in hot and humid climates; therefore, natural ventilation alone is insufficient, and instead, Bio-PCM and insulation optimize indoor temperature regulation. During the day, the suggested model demonstrated a 61% electricity reduction when compared to the conventional building with air conditioning. On the experimental side, Abbas et al. [176] explored PCM capsule use as an insulation material within the hollow bricks that form a wall. The study focus on how the PCM capsules can enhance energy efficiency, improve indoor thermal comfort and reduce heat transfer, especially in hot climates like Iraq. The PCM composition was paraffin wax encapsulated in sealed aluminum tubes. They developed a 3D mathematical model using FORTRAN programming language to simulate the thermal behaviour of walls and the natural convection inside the rooms. Also, the research designs and fabricates a novel “Plug and Play Wall” system to test the thermal performance of walls with and without PCM capsules under natural outdoor conditions. The findings indicate that adding PCM to the treated wall will lower the room and inner surface wall temperature by approximately 4.7 °C, prolong the time lag by two hours, reduce temperature fluctuations by 23.84% and have a decrement factor of 0.7.

Extending this evolution of MPCM research into emerging materials, Zhilyaev et al. [177] explores the development and performance evaluation of NRG-Foam, where MPCM is used in an emerging cementitious foam insulation material. Research focused on modelling its economic, thermal, and environmental properties, locating hotspots in the production process and weighing performance characteristic trade-offs. The material consists of a cementitious foam incorporating MPCM. The NRG-foam is a promising insulation material, combining thermal insulation and energy storage through MPCM. The environmental impacts were evaluated by preparing an LCA of the NRG-Foam with software OpenLCA 2.6.0; it was demonstrated that the production of MPCM accounts for up to 95% of the total impacts, depending on the foam configuration and impact category. While switching to renewable energy can minimize its impact, increasing MPCM content must achieve a balance between sustainability and thermal performance. Using low-embodied carbon materials and optimizing encapsulation techniques can improve environmental efficiency. The insulating materials are summarized in Table 14 using different encapsulation materials, and MPCM in buildings. These studies describe a progression from simulation-based optimization to experimental validation and sustainable material innovation, signifying an integrated path toward low-energy, thermally stable, and environmentally responsible building design.

### 5.11. Building Envelope Retrofits

The building envelope consists of walls, floor, windows, and the roof; it is a crucial aspect of the design since it can affect the building’s energy performance. To create a comfortable home all through the year, the following features are necessary: low thermal bridging, high insulation levels, ventilation, and summer shading attributes [178]. Hence, the integration of PCM into building envelopes aims to enhance energy efficiency. This can be achieved through different methods, including direct integration, where PCMs are applied as separate layers within building materials, or mixed-component integration, where they are blended into building materials to increase thermal mass and improve heat storage capacity [179].

Several investigations have focused on the applicability of PCM under different climatic conditions. Berrocal et al. [180] evaluated five PCM types (paraffin wax P56-58, RT21, PCM1, Dupont Energain, and BioPCM) under the climate of Panama City using DesignBuilder 7.3 software. Results show that Dupont Energain performed the best, with energy savings of 22.93% in Panama’s climate; nevertheless, the PCM placement and thickness should be optimized for different climates. Likewise, Al-Yasiri et al. [173] investigated the integration of traditional EPS with thermal insulation in building envelopes to improve thermal efficiency and indoor comfort in extreme summer conditions. They determined how different EPS insulation thicknesses affect the thermal performance of the building by using EnergyPlus simulations. By combining PCM and EPS in the insulation, the energy consumption of the building decreased and thermal performance improved. According to the study, an EPS thickness of 1 cm demonstrated a better achievement considering the average temperature fluctuation reduction and average operative temperature reduction during the whole thermal cycle; furthermore, the location of PCM within the building envelope has a big influence on efficiency, as shown in Figure 7b. The results show that PCM-EPS systems are a viable way to improve sustainability and energy efficiency in nearly zero-energy buildings. Likewise, Benachir et al. [181] explored the energy performance of building envelopes by integrating solar ventilation and PCM. In which they focused on the improvement of thermal efficiency, energy savings, and occupant comfort using dynamic thermal simulation and dynamic energy simulation. They also evaluated the impact of PCM and solar ventilation on thermal behaviour and energy demand using TRNSYS and EnergyPlus. The integration of PCM and solar ventilation reduces demand and optimizes energy efficiency. However, passive cooling strategies, including PCM-enhanced envelopes, provide a sustainable solution for buildings. Meanwhile, Khan et al. [174] examined the potential energy savings that can be achieved by incorporating PCM into residential building envelopes in five main cities with different climates in Pakistan. The impact of PCM type, thickness, and placement in single-story and two-story residential buildings is addressed in the study using EnergyPlus models. Static and dynamic payback period estimates are used to assess the economic viability of PCM integration. In a single-story building, it achieved an average monthly energy saving of 49.6% in Quetta, 35% in Peshawar, 32% in Lahore, 35% in Karachi, and 44.9% in Islamabad. Depending on the city, savings for two-story buildings ranged from 12% to 21.4%, and the graph of average heat gain reduction is shown in Figure 7c. It was determined that the best simulation PCM thickness is 40 mm. Then, using PCM to improve the energy efficiency of residential buildings is economically viable in Lahore, Karachi, and Peshawar, but unsuited in Islamabad and Quetta.

Further contributions from Mousazadeh et al. [182] and Beiranvand et al. [183] reinforced the versatility of PCM integration in diverse contexts. Mousazadeh et al. [182] reported how PCM integration in building envelopes can enhance energy efficiency and reduce cooling and heating loads. The use of DesignBuilder simulation was done to focus on Iran’s climate as a case study. In contrast to nanopaint, which lowered energy use by 1.8%, PCM integration in the building envelope decreased energy consumption by 6% and Energy Use Intensity by 5.6%. Additionally, PCM reduced temperature swings by up to 8 °C, improving thermal comfort. In areas with low energy costs, however, economic viability is a concern because of the lengthy payback period of 94 years and the high starting cost of $467,918. Nevertheless, PCM turned out to be the most effective and sustainable thermal management option, meeting international energy efficiency standards.

Beiranvand et al. [183] examined the integration of PCM into building envelopes to enhance energy efficiency and thermal comfort. Using DesignBuilder software, which runs on the EnergyPlus simulation engine, they examined the impacts of several PCM configurations on a commercial office building in Torbat Heydarieh, Iran. To identify the best PCM type and layer arrangement for reducing energy consumption, the study evaluates PCM performance during the heating and cooling seasons. The incorporation of PCM in commercial buildings reduced energy consumption by up to 3.8% with PCM23, whose melting point is at 23 °C, being the most effective at stabilizing indoor temperatures. Heat transfer and thermal storage regulation are improved by using multiple PCM layers. While PCM is a promising passive energy-saving strategy, its placement and material selection are key to maximizing efficiency. However, Hagenau et al. [184] performed a performance evaluation of building envelope enhanced with PCM under Danish conditions. The research focuses on Danish climate conditions and analyzes PCM performance through dynamic energy modelling using EnergyPlus simulations. To determine the most effective PCM, different PCMs were tested in a Danish office building that served as the case study. To determine the ideal PCM thickness and location within the building envelope, the study also performed a parametric analysis. EnergyPlus was used as a simulation engine to predict the impact of incorporating PCM within building components. It was discovered that modelling the effects of incorporating the PCM into the building envelope under the weather profiles of different Danish cities produced comparable energy savings in the range of 32% to 36%. The optimal performance was achieved with a 40 mm PCM layer applied to the interior side of walls and roofs. Additionally, finding the best-performing PCM for Danish structures is demonstrated to be significantly influenced by the building insulation level, room cooling and heating set-points, and ambient weather conditions.

Kulumkanov et al. [185] explored the impact of PCM on building energy efficiency and environmental sustainability, especially in the context of the future climate scenarios (2095) across 13 different climate zones worldwide. Regarding the research, it focuses on how PCM can reduce energy demand for cooling, heating, and overall annual energy consumption in buildings, while also evaluating the potential to reduce carbon emissions. Also, a machine learning-based model was developed to predict future CO_2_ emissions and assess the environmental benefits of PCM integration in buildings. However, energy simulations were conducted using EnergyPlus software to assess the energy performance of PCM-integrated buildings under future climate conditions. According to the environmental analysis, PCM integration can reduce carbon emissions by up to 204 CO_2_-eq kg/(m^2^·year). High accuracy (R2: 0.7–0.99) was demonstrated by the prediction-based CO_2_ prediction model, guaranteeing accurate future energy and environmental projections. The integration of PCM and MPCM into building envelopes enhances energy efficiency and thermal comfort. Future progress depends on optimizing materials and configurations while leveraging advanced encapsulations and AI-based control for adaptive, low-carbon buildings. Moreover, the building envelope retrofitting has been summarized in Table 15 for different PCMs/MPCMs.

The direct quantitative comparisons of the thermal performance of MPCM are fundamentally constrained by the significant heterogeneity among the reported studies which include differences in PCM chemistry, encapsulation shell materials, PCM loading levels, specimen scale, and experimental or numerical boundary conditions. Therefore, the reported outcome indicators, such as peak temperature reduction or energy savings, should be interpreted as system-specific results than the universal benchmarks. Despite this variability, the strong consistency of qualitative trends observed across independent experimental and numerical studies confirms the robustness of MPCM as an effective strategy for increasing the thermal energy storage and passive thermal regulation in non-structural building components.

The tables in this review provide a comprehensive overview of the applications of MPCM in the various construction materials, including cement, roofs, bricks, walls, windows, paints, gypsum boards, building envelopes, and insulating materials. The findings highlight significant improvements in thermal performance and energy efficiency. Incorporating MPCM into cement and bricks reduces thermal conductivity, enhancing heat storage and improving indoor temperature regulation. In roofs and envelopes, MPCM helps stabilize indoor temperatures, decreasing the need for heating and cooling systems. Paints infused with MPCM mitigate surface temperature peaks, while PCM-integrated windows enhance insulation without compromising natural light transmission. Additionally, gypsum boards with MCPM improve thermal inertia, further contributing to energy savings. These results underscore the potential of MPCM-enhanced materials for sustainable and energy-efficient buildings, although it is necessary to refine their durability, compatibility, and structural performance.

## 6. Product of PCM/MPCM Depending on the Region

Table 16 provides a comprehensive review of experimental and simulation studies integrating PCM and MPCM into diverse building parts such as cement, mortar, concrete, walls, roofs, windows, etc. Each data set describes the connection between PCM (e.g., paraffins, synthetic waxes, bio-based encapsulated materials, or stabilized microspheres), characteristics of Tm, LHS capacities, and the corresponding climatic conditions under which they were evaluated. PCM functions as dynamic thermal insulation by absorbing excess heat during peak periods and releasing it during cooler intervals. This thermal modulation improves indoor comfort while using much less energy for heating and cooling. The incorporation of PCM into construction systems strongly constitutes a critical step toward sustainable, low-carbon, and energy-efficient building design that is suited for a wide range of regional climate conditions. The type of PCM selected depends on the prevailing climatic conditions. In hot and semi-arid regions such as Tunisia, Iran, Iraq, Morocco, and northern Chile, PCM with medium to high melting points (25–32 °C) are commonly used. These are mainly paraffin-based PCM (RT25, RT26, RT28) or microencapsulated composites such as CaCO3-MPCM and BioPCM, designed to promote passive cooling and heat mitigation in roofs, walls, and reflective coatings.

In temperate climates, such as those found in Spain, Turkey, and Bulgaria, PCM with lower Tm (18–23 °C) like Micronal^®^ DS 5038X or Nextek^®^ MPCM are integrated into cement-based or translucent gypsum panels to stabilize indoor temperatures during seasonal transitions. On the other hand, in continental climates, including northern and central China, Denmark, and northern Europe, PCM with high LH values (180–250 kJ·kg^−1^) are preferred, as they are incorporated into insulation layers, ventilated roofs, and double-glazed window, helping to retain heat during the winter. Finally, in tropical or humid zones, such as Panama and southern China, PCM are combined with aerogels or reflective layers to balance the thermal loads throughout the year and significantly reduce annual energy consumption.

Integrating MPCM into building construction offers adaptable, effective, and climate-responsive solutions for enhancing energy efficiency. The reviewed research shows that matching the MPCM type and melting range to local thermal conditions can cut heating and cooling energy use by up to 50%, while improving thermal comfort and extending component durability.

Climate-specific MPCM design is key to creating energy-efficient, sustainable, and resilient buildings. This approach directly aligns with global goals for carbon reduction, energy storage innovation, and next-generation net-zero architecture.

## 7. Conclusions

The potential of MPCM has been critically evaluated when mixed with building materials, focusing on the energy consumption, thermal, and environmental properties of different materials on walls, bricks, roofs, envelopes, floors, windows, paint, gypsum boards, insulating materials, and cement. Herein, this study explores the general overview of the MPCM types and their uses, microencapsulation techniques, pros and cons of different encapsulated PCM, simulation software, and their implementation in different building materials.

The integration of MPCM into building materials can effectively introduce a significant time lag, which reduces the reliance on heating and cooling systems, leading to energy savings. MPCM can boost the indoor thermal comfort in buildings when it is integrated into building materials in appropriate quantities and optimized locations. In addition to the appropriate quantity and location of MPCM in a matrix for building applications, energy savings are highly dependent on environmental conditions. For instance, an optimal ambient temperature during the day and night allows for complete heating and cooling of the PCM, ensuring its effective utilization. Nevertheless, energy-saving potential varies depending on location and specific environmental conditions.

Furthermore, the effectiveness of MPCM-based thermal energy storage is strongly influenced by the climatic conditions. The most promising research studies shows suitable performance in Mediterranean, semi-arid, and degraded oceanic climates, where moderate diurnal temperature variations enable actual charging and discharging of MPCM. Meanwhile, in hot dry regions, a reduced effectiveness is often observed. While the underlying MPCM thermal mechanisms are transferable across different climate zones, the magnitude of performance gains is highly climate-specific and depends on PCM melting temperature, diurnal temperature swing, and building operation. Therefore, the MPCM performance across the diverse thermal boundary conditions should be approached with caution, and the climate adaptive PCM selection and system design are essential for reliable energy savings. The analysis of PCM/MPCM products across different climates reveals that paraffin-based PCMs are well suited for hot and semi-arid regions due to their passive cooling capability; MPCM with melting points near 18–23 °C perform optimally in Mediterranean and temperate zones; and high LH PCMs (180–250 kJ·kg^−1^) are most effective in cold climates, supporting passive heat retention. Such evidence confirms that climate-adapted PCM design is essential for maximizing energy efficiency and thermal performance.

Therefore, it can be concluded that MPCM is compatible with multiple construction materials like walls, envelopes, paint, gypsum boards, floors, windows, bricks, roofs, insulating materials, and cement for enhancing the thermal performance in buildings. In addition, by incorporating MPCM into gypsum wallboard, the thermal conductivity of the material is reduced. This occurs due to the low heat transfer rate of both PCM and MPCM polymeric shells. Moreover, the formation of air voids caused by the inclusion of MPCM further contributes to reduced thermal conductivity. A hybrid PCM system offers an effective approach for regulating indoor temperatures in buildings located in regions with large diurnal temperature variations; such systems can also effectively adapt to seasonal temperature changes.

Some simulation software are valuable tools for validating experimental results, but their accuracy depends on selecting climatic conditions that closely match the environmental conditions. Then, the development of an environmental assessment by employing an LCA for the incorporation of MPCM in buildings will help address the lack of environmental data, particularly for PCMs. LCAs provide accurate and objective conclusions and recommendations for practice.

Although PCM/MPCM-based retrofitting solutions show considerable promise, long-term in-service data concerning durability, thermal cycling stability, maintenance, and performance degradation are still limited. Therefore, the sustainability and carbon reduction potentials identified in this review should be considered indicative trends rather than definitive outcomes. Future work should focus on long-term testing and monitored pilots to strengthen life cycle and carbon evaluation.

Within the context of the review, polymers can be seen as the key link between the intrinsic potential of PCM/MPCM and their practical use in real buildings. By acting as shape-stabilizing matrices and encapsulation shells, they make it possible to embed latent heat storage in walls, roofs, gypsum boards, and other components without compromising structural integrity or causing leakages. Across the reviewed studies, polymer properties especially thermal stability, mechanical strength, permeability, and compatibility with cementitious, gypsum-based, and polymeric matrices are shown to critically govern the energy performance, durability, and operational reliability of PCM-enhanced building components.

At the same time, the growing interest in recycled, bio-based, and hybrid polymer systems reflects a clear shift toward improving not only performance but also the environmental profile of PCM/MPCM technologies. When combined with climate-specific design, building energy simulation, and LC, the rational selection and engineering of polymers emerge as a decisive step for advancing MPCM-based solutions from promising laboratory concepts to robust, low-carbon technologies for climate-responsive buildings. In conclusion, the polymeric shell plays a decisive role in governing the performance, durability, and compatibility of MPCM within building envelopes. Its chemical stability, mechanical integrity, and interfacial behaviour directly influence both thermal functionality and composite reliability.

## Figures and Tables

**Table 1:** Conditions of the PCM for use in building applications.

Property Category	Criteria	Description
Thermodynamic Properties	Phase change temperature.	Must be suitable for the intended application.
	Latent heat and specific heat.	High LH per unit volume and high specific heat enhances ES.
	Thermal conductivity.	Higher thermal conductivity improves heat transfer efficiency.
	Volume and vapour pressure.	Small volume changes and low vapour pressure prevent structural issues.
Kinetic Properties	Nucleation rate.	A high nucleation rate minimizes supercooling.
	Crystal growth rate.	Fast crystal growth supports efficient heat recovery.
Chemical Properties	Toxicity and safety.	Should be non-toxic, non-flammable, and non-explosive.
	Material compatibility.	Must not corrode or degrade construction materials.
	Chemical stability.	Needs to maintain stability over long periods and reversible phase changes.
Economic Properties	Cost and availability.	It should be cost-effective and commercially available.
Environmental and Safety Properties	Sustainability.	It should have a low environmental impact and be recyclable.
	Fire safety.	Must comply with fire safety regulations.
	Encapsulation compatibility.	Must work well with its shell material to prevent leakage.

**Table 2:** Pros and cons of organic, inorganic, and eutectic PCM mixtures.

PCM	Pros	Cons
Organic (OPCM)	Available in a large temperature range.	Highly flammable.
	Chemical stability and recyclability.	Low thermal conductivity.
	Small volume change and low vapour pressure.	Leakage during phase transition.
	Long melt–freeze cycle.	Instability at high temperature.
Inorganic (IPCM)	High LH of fusion and thermal conductivity.	Incongruent melting.
	Inexpensive and low toxicity.	Subcooling and phase segregation.
	Low volume change.	Leakage during phase transition.
Eutectic (EPCM)	Chemical and thermal stability. No or little subcooling. Environmentally friendly.	Lack of thermophysical property test data currently available. Leakage during phase transition.

**Table 3:** Pros/cons and applications depending on the encapsulation type.

Encapsulation Type	Pros	Cons	Applications
ePCM	Simple and cost-effective. Easy to handle and integrate into applications. Large PCM storage capacity per unit. Suitable for large-scale TES.	Low heat transfer efficiency due to limited surface area. Higher risk of leakage if the shell is damaged. Bulky size limits are used in compact applications.	Building materials (e.g., wall panels, ceiling tiles). TES in solar heating systems. Large-scale waste heat recovery.
MPCM	Improved heat transfers due to higher surface area. Prevents leakage from PCM. Enhances thermal stability and durability. Can be integrated into textiles, coatings, and construction materials.	Complex manufacturing processes increase costs. Limited PCM loading capacity due to shell thickness. Potential risk of shell degradation over time.	Textile fibres for thermal comfort. Smart coatings for energy-efficient buildings. Thermal-regulating packaging materials.
NEPCM	Extremely high surface area for superior heat transfer. Enhanced thermal conductivity and phase change efficiency. Increased PCM stability at the nanoscale. Improvement in the mechanical and chemical stability of PCM.	Complex and expensive production. Stability issues like agglomeration of nanoparticles. Potential health and environmental concerns due to nanoparticle exposure. Difficulties in large-scale implementation.	Advanced thermal nanofluids for cooling systems. High-performance electronics thermal management. Medical applications (drug delivery, temperature-sensitive treatments). Nanocoatings for heat regulation in smart fabrics.

**Table 4:** LCA software.

Software	Key Advantages	Key Disadvantages
SimaPro 10.3	- Used in academia. - Exhaustive and detailed. - Multiple impact assessment methods. - Ecoinvent and European Life Cycle Database access.	- Expensive. - High learning curve. - Slightly complex interface.
Sphera® LCA for Experts 10.7/GaBi	- Excellent for industrial processes. - Comprehensive database. - Clear visualization of the result.	- Expensive and complex set-up. - Requires specialized training.
OpenLCA 2.6.0	- Open-source and free. - Compatible with multiple databases and flexible. - Ideal for academic research.	- Requires manual data set-up. - Limited technical support. - Slow calculations and low level of automation.
One Click LCA (https://oneclicklca.com/, accessed 5 February 2026)	- BIM and compatible with construction projects. - Regulatory compliance and automates data capture.	- SimaPro/GaBi has a deeper methodological approach. - Platform dependent and subscription cost.

**Table 6:** Application of mortars using MPCM.

Authors	MPCM	Conditions	Thermal Performance Study	Observations	Ref.
Gbekou et al. (2022)	CrodaTherm™ ME29D	MPCM slurry containing 50 wt% solids, with a PCM-to-acrylic polymer shell mass ratio of 92:8.	Evaluation of the thermal and mechanical properties of MPCM-integrated composite cement mortar.	Thermal conductivity decreases by 29%, improving insulation. The optimal balance is found at 8 wt% MPCM, maintaining structural integrity while maximizing thermal benefits. There is potential in the MPCM-enhanced mortars for energy-efficient, non-load-bearing applications.	[122]
Salihi et al. (2022)	Rubitherm RT-HC paraffin-based PCM, including RT-18 HC (17–19 °C) RT-21 HC (20–23 °C) RT-25 HC (22–26 °C) RT-28 HC (27–29 °C) (Optimal choice) RT-35 HC (34–36 °C)	MPCM within aluminum plates.	MPCM integration reduced peak indoor temperature by 1.64 °C during the day and increased nighttime temperatures by 1.37 °C, enhancing thermal comfort. RT-28 HC showed the best performance with an annual average temperature fluctuation reduction of 1.91 °C.	MPCM performance depends heavily on T_m_ and seasonal variations. RT-28 HC is the most efficient PCM for the semi-arid climate of Benguerir. Multi-layer PCM configuration (triple-layer) outperforms single-layer systems, providing more consistent thermal comfort year-round.	[120]
Gbekou et al. (2024)	CrodaTherm™ ME29D, a bio-based MPCM derived from plant-based feedstocks	MPCM within a formaldehyde-free acrylic polymer shell.	The MPCM-integrated mortar (M15D) shows a temperature reduction of up to 1 °C compared to the reference wall during heating. Achieved 33% energy savings for heating and 31% for cooling, especially in Mediterranean and degraded oceanic climates.	MPCM performed best in climates with daily temperature variations, like Mediterranean regions, but was less effective in hot dry climates due to continuous high temperatures preventing solidification. The study validated EnergyPlus and COMSOL Multiphysics models, showing less than 0.5 °C error compared to experimental results.	[123]

**Table 7:** Concrete applications in buildings using different MPCM.

Authors	MPCM	Conditions	Thermal Performance Study	Observations	Ref.
Kumar et al. (2023)	Capric Acid (CA).	FSPCM	Two mortar panels (20 × 20 × 2 cm^3^) were prepared, one with FSPCM (12 wt%) and one as reference mortar. Tested under simulated summer conditions (15–35 °C).	FSPCM offers the highest thermal inertia (1.32 °C) and heat storage (690 kJ·m^−3^) but has the lowest compressive strength (3.66 MPa). FSPCM provides a balanced performance with moderate compressive strength, thermal inertia, and heat storage.	[121]
Lajimi et al. (2023)	Paraffin RT25.	MPCM	The aim of the research is to study the heat and mass transfer through ceiling wall containing MPCM under realistic climatic conditions in Tunisia and compare the thermal performance of a wall with PCM (PCC) versus a wall with glass wool insulation.	The use of MPCM in the ceiling reduced energy consumption, improved thermal performance, and enhanced comfort by effectively managing temperature and humidity, especially during summer. Economic analysis demonstrated that PCC is a cost-efficient solution for energy savings in buildings.	[126]
Cabeza et al. (2020)	Micronal DS 5001.	MPCM	During daytime heat storage and nighttime heat release, the MPCM successfully decreased indoor temperature swings. In contrast to the traditional concrete cubicle, the MPCM cubicle’s internal ambient temperature was lowered, with daily variations never rising over 4 °C. Compared to traditional cubicles, the MPCM cubicle’s peak temperatures were 2 °C lower.	The temperature differential between the MPCM cubicle and the conventional cubicle grew to 5–7 °C in 2016 from 1 to 3 °C in 2005, indicating that the MPCM was more successful in extreme weather situations (higher external temperatures). By lowering the demand for active cooling systems, the MPCM cubicle showed increased thermal comfort and energy efficiency.	[124]

**Table 8:** Application of walls in buildings using different MPCM/PCM.

Authors	MPCM	Conditions	Thermal Performance Study	Observations	Ref.
Tabares-Velasco et al. (2012)	PCM distributed in drywall (30% by weight). PCM distributed in fibrous insulation (20% by weight). Concentrated PCM layer (100% PCM, 0.5 cm thick).	Polymer-based MPCM.	The MPCM layers successfully postpone peak heat flux by four hours and lower peak interior temperatures by 0.5 °C. According to EnergyPlus calculations, attic applications might save up to 19–57% on energy cost, and summertime peak load reductions could be possible.	For simulating MPCM-enhanced wall assemblies, the EnergyPlus MPCM model is dependable. Performance is affected by the MPCM distribution strategy; concentrated MPCM layers exhibit slower response times but greater heat storage. Accuracy in simulations is greatly impacted by node spacing. Model dependability is enhanced with shorter time steps (≤3 min).	[127]
Alam et al. (2014)	Micronal DS 5008X PCM, a MPCM paraffin-based produced by BASF company.	MPCM within a polymeric shell.	MPCM integration in building walls resulted in energy savings ranging from 5% to 25%, depending on the climate, with higher efficiency in cooling dominant regions.	In a variety of conditions, MPCM integration improves building energy efficiency. In warmer cities, cooling energy demand decreases were greater than heating demand reductions.	[129]
Zhuang et al. (2010)	Used substances like lipids, polymers, or inorganic materials, providing a protective barrier and enhancing integration into building materials.	MPCM	By dampening indoor/outdoor temperature swings, the MPCM integration offers up to 30% energy savings over conventional methods. It reduces energy loss during phase transitions and enhances thermal storage.	The study emphasizes the thermal efficiency and energy-saving advantages of MPCM, highlighting their potential in building envelopes. The study also emphasizes how crucial appropriate MPCM implantation and integration methods are peak performance.	[130]
Kuznik et al. (2008)	MPCM of 60% paraffin embedded in an ethylene-based copolymer (40%).	MPCM is produced by DuPont de Nemours.	When compared to walls without MPCM, the MPCM walls decreased variations in the air temperature by as much as 4.7 °C. The MPCM-containing room had a temperature range of 19.8 °C to 32.8 °C, while the PCM-free room had a temperature of 18.9 °C to 36.6 °C.	Furthermore, the MPCM walls improved the room’s thermal stratification. The temperature differential between the lower and top portions of the room was 1 °C in the MPCM-equipped room, while it was reduced in the MPCM-equipped room.	[131]
Chan et al. (2011)	MPCM used was Energain**^®^**, produced by DuPont de Nemours.	60% MPCM with paraffin within an ethylene-based copolymer 40%.	The internal surface temperature was dropped by the MPCM integrated walls; during peak hours, the west-facing wall showed the greatest decrease, lowering by up to 4.14%. The east-facing wall did well, reducing by 4.09%. A/C energy consumption was reduced by 2.9% annually due to the west-facing MPCM wall.	The performance of the MPCM integrated walls was greatly impacted by their orientation. The high initial cost of the MPCM wallboard (USD 70/m^2^) made the MPCM integrated facade economically unfeasible, with an anticipated 91-year simple payback period.	[132]
Campbell et al. (2011)	BioPCM™ from phase change energy with different PCM T_M_: 23 °C, 25 °C, 27 °C and 29 °C.	ePCM and derived from refined soy and palm kernel oil.	Portland, Oregon: With a 93% drop in zone-hours and a 98% drop in zone-degree-hours beyond the thermal comfort zone, ePCM with a melt temperature of 25 °C produced the biggest reduction in thermal discomfort. Los Angeles: When applied to the second-floor common area, PCM decreased heat discomfort by 44% zone-hours and 55% zone-degree-hours. Denver: Using PCM at a melt temperature of 25 °C, 79% zone-hours and 89% zone-degree-hours reductions were obtained. Phoenix: ePCM had little effect because prolonged high temperatures prevented adequate ePCM discharge.	Across all cities, PCM installation in second-floor common rooms produced the most gains in thermal comfort. For PCM recharge and efficient thermal performance, cool evening temperatures were essential. In Phoenix, annual energy savings were 0.5%, while in Denver, it was 3.1%. When compared to MPCM, ePCM was 2.5 times more affordable per kJ stored.	[133]
Dardouri et al. (2023)	Infinite R™ PCM, The PCM have different T_m_. PCM18, PCM21, PCM23, PCM25, and PCM29.	No shell.	Energy use for heating and cooling decreased when PCM is included into walls and roofs. The performance of the PCM layer depends on whether it is located near the exterior or the interior of the wall. Higher energy savings were achieved by positioning the PCM close to the interior surface, particularly for heating.	The environment determines the ideal T_M_ for PCM. T_M_ < 21 °C work better for heating, while T_M_ > 29 °C work better for cooling. Environmental factors have a considerable impact on PCM performance. PCM21 was better at heating in colder climates, whereas PCM29 was better at cooling in warmer climates.	[134]
Xue et al. (2024)	Nextek 18D (Paraffin-based).	MPCM in plaster composite.	A modular cooling wall prototype with MPCM-plaster composite and capillary pipe mat was tested in a climate-controlled room. Higher MPCM content increased thermal inertia, slowing down temperature recovery during discharge.	Lower feed water temperatures (5 °C) significantly enhance cooling power (53–70 W·m^−2^), while higher MPCM content (30%) increases thermal storage capacity but reduces cooling power by ~24%. Optimal setups vary residential applications prioritize rapid thermal response, whereas industrial settings require feed water temperatures matching waste heat levels.	[125]
Babaharra et al. (2021)	n-octadecane paraffin.	Encapsulation material: PMMA with a thin layer (0.1 μm to 0.2 μm).	To evaluate the thermal performance of a multilayer wall with MPCM in a hot climate zone (Khouribga, Morocco).	The integration of MPCM in the hollow brick offers the best thermal performance, reducing heat flux by 30% and providing a 3 h time lag. A 20% mass fraction of MPCM is optimal for both thermal and mechanical performance. MPCM helps stabilize indoor temperatures, particularly during peak summer days, enhancing thermal comfort.	[135]

**Table 9:** Application of roof in buildings using different MPCM/PCM.

**Authors**	**PCM**	**Conditions**	**Thermal Performance Study**	**Observations**	**Ref.**
Dardouri et al. (2023)	Infinite R™ PCM (commercial; composition not specified) with diverse T_M_ (18, 21, 23, 25 and 29 °C)	No shell	Evaluates the impact of integrating Infinite R™ PCM into walls and roofs to improve energy efficiency and indoor temperature regulation by using EnergyPlus Simulation.	Energy consumption during heating and cooling is substantially reduced by PCM integration; the optimal savings take place at phase transition temperatures based on seasonal requirements. PCM layers improve thermal comfort and lessen temperature swings, indicating their potential for usage in energy-efficient construction applications.	[134]
Chen et al. (2023)	SSPCM	No shell	Employing numerical simulation to develop a roof using PCM composite and CP in conjunction.	Although PCM + CP works better in areas with high cooling demand, PCM alone works better in areas with high heating energy usage. Energy demand is continuously decreasing by increasing PCM thickness, although the effect eventually stabilizes. The research offers guidance for choosing the ideal PCM thickness and phase transition temperature, enhancing building applications’ cost effectiveness and energy efficiency	[136]
Yu et al. (2023)	Paraffin (85%) + HDPE (15%)	SSPCM	Evaluates a ventilated roof system incorporating SSPCM. It reduces cooling energy demand and indoor temperature fluctuations.	Through night ventilation, the roof design optimizes heat dissipation and raises thermal efficiency overall. The SSPCM layer effectively improves energy savings and thermal comfort in hot areas by delaying peak interior temperatures by at least three hours and lowering ceiling temperatures by up to 2.38 °C, based on simulation and experimental studies.	[137]
Luo et al. (2023)	Paraffin wax	Porous bricks	Porous bricks filled with paraffin waxes are investigated numerically for significant temperature variations throughout the day.	The responsiveness of the phase transition temperature to external variables is crucial for the use of PCM in roofing, as it proves PCM’s climate-adaptability.	[139]
Jia et al. (2021)	Cetane used as PCM	-	It was examined how well prefabricated structures using PCM function thermally and energy-efficiently across five distinct climate zones.	It is more efficient to place PCM inside the building envelope rather than outside to save energy. Climate affects the ideal PCM thickness; nevertheless, the most effective range is between 10 and 30 mm. Furthermore, because of their exposure to sunlight, the east and west-facing walls offer the greatest potential for energy savings.	[140]

**Table 10:** Application of windows in buildings using different PCM.

Authors	MPCM	Conditions	Thermal Performance Study	Observations	Ref.
Uribe et al. (2021)	Paraffin RT25 Rubitherm	The PCM is integrated into the double-glazed window of the office room, replacing the air gap in the reference room.	The experiment was one-year real scale conducted in Santiago, Chile, which has a semi-arid climate, characterized by high solar radiation and significant temperature variations between day and night.	PCM glazing decreased summer peak heat loads, increased thermal comfort, and used less energy for HVAC systems.	[138]
Heim et al. (2021)	Paraffin RT21HC	Unit with triple-glazing and one PCM-filled hollow.	The experiments were conducted in a laboratory setting using an artificial sun as a radiation source. The artificial sun consists of eight halogen lamps with a total power of 8 kW, providing controlled and stable irradiation.	PCM melted in a complicated and dynamic process that went through the following states: solid, mushy, semi-mushy, semi-liquid, and liquid.	[141]
Ma et al. (2022)	Solid–solid (S-S) PCM	A new kind of glazing window containing silica aerogel and S-S PCM.	A numerical assessment of revolutionary glass windows’ energy-saving potential, thermal efficiency, and design in China’s severe winter climate.	The building with the innovative glazing window achieved a maximum energy saving of 18.22% compared to a 4 mm single glazing window (SLGW).	[142]
Gao et al. (2021)	Solid–solid (S-S) translucent PCM	The integration of translucent PCM into double-pane windows for commercial buildings. The goal is to improve energy efficiency by managing TES and solar radiation.	Simulations employing EnergyPlus 9.4 software to comprehend the S-S transparent PCM’s thermal performance.	Using 3 mm thick S-S PCM in windows for different climates can save HVAC energy. In mixed, warm, and cold climates: 14%, 17.2, and 5.8%, respectively.	[143]
Wijesana et al. (2020)	PEG + CNF SSPCM	SSPCM composite inside a smart window.	Switchable transparency window based on PCM/polymer.	The optimally constructed window transmittance increased from 3.5%, which was below PCM’s melting point, to 88%, that was above it.	[144]
Chen-Pan et al. (2023)	Two PCM paraffin wax MG-29, Octadecane	Window covered with PCM equipped shutter system.	Numerical modelling of the performance of window shutter PCM systems in two Mexican cities during warm (Merida) and cold (Toluca) conditions.	On both days, the shutter with PCM MG-29 performed at its best in both cities. It was advised to utilize this PCM window shutter system in warm climates.	[145]
Roy et al. (2022)	In_2_O_3_/ZnO polymethylmethacrylate paraffin composite	Developing a multifold smart composite for smart glazing applications in buildings.	The composite-coated glass was tested in a prototype double-glazed window to evaluate its thermal performance. The temperature difference between the indoor and outdoor surfaces was measured over time.	The In_2_O_3_/ZnO (5 wt%) composite film showed the best balance between visible light transmittance and near-infrared (NIR) shielding. The transparency reached ~86% at 60 °C, while at 22 °C, it was ~64%.	[146]

**Table 11:** Application of bricks in buildings using different MPCM/PCM.

Author	PCM	Shell	Numerical Study	Observations	Ref.
Mahdaoui et al. (2021)	N-nonadecane	Hollow clay brick.	A numerical study is carried out using Ansys Fluent to assess the thermal behaviour of clay hollow brick impregnated with MPCM.	For modelling the phase change process, an enthalpy-porosity-based method was adopted.	[147]
Dabiri et al. (2018)	RT-35	Steel with 0.6 mm thickness.	Thermal analysis of a brick incorporating PCM and ten air cavities to reduce heat transfer between outdoor and indoor spaces. Computational fluid dynamics was used to simulate the behaviour of the PCM under time-dependent external conditions.	Incorporating PCM into brick reduced the amplitude of indoor temperature fluctuations by 48.5% in summer and 44% in winter.	[148]
Al-Yasiri et al. (2021)	Paraffin wax	Aluminum bars and feature a square cross-section of 1 mm thickness.	Thermal performance of bricks incorporating PCM under hot climate conditions in Iraq. (1) Reference (brick without PCM); (2) bricks which contain one bulky PCM capsule (4 × 4 × 10 cm^3^)—bricks which contain two PCM capsules (4 × 4 × 5 cm^3^); (3) brick which contains 5 PCM capsules (4 × 4 × 2 cm^3^).	The brick which contains 5 PCM capsules is the most effective option for reducing indoor temperatures and improving energy efficiency in buildings.	[149]
Aakash et al. (2021)	Paraffin-based PCM (RT from Rubitherm)	No shell	Numerical analysis of the thermal performance of several wall designs with PCM and insulation added.	The combination of PCM with night ventilation did not work. The PCM’s LH consumption is dependent on their melting point and wall placement.	[150]
Mukram et al. (2024)	MPCM (MEP29)	Polyethylene or PUR.	CFD analysis using ANSYS Fluent, validated with psychrometric chamber experiments.	PCM in middle of brick (mPCM75) provides the best performance, suitable for hot climates	[151]
Fraine et al. (2019)	MPCM/diatomite	Sintered hollow bricks	Numerical analysis of PCHCM’s potential to replace EPS in Algerian buildings.	A total of 66% of the PCHCM should be filled inside the hollow bricks for best results. Comparing novel PCHCM to EPS, energy savings of 50% were possible.	[152]

**Table 12:** Application of paints in buildings using different MPCM/PCM.

Author	PCM	Shell	MPCM/ Composite	Paint	Observations	Ref.
Ghayedhosseini et al. (2024)	OPCM: Paraffin C16-C18 (basic PCM), ATP20 (hysteresis PCM) and ATP23 (hysteresis PCM)	No shell.	ePCM in wallboards.	Types of CP: Black, White, and Grey.	CP are highly effective in reducing cooling loads during hot seasons but increase heating demand in colder months, while PCM provides year-round benefits by reducing both cooling and heating loads, especially when placed in the inner wall layer. The combined use of White CP and ATP23 PCM offers the best results, significantly lowering energy demand and peak loads.	[156]
Qin et al. (2022)	Octadecane	Silica (SiO_2_).	MPCM by interfacial hydrolysis and poly-condensation.	Acrylic resin-based paint (70 wt% MPCM).	On high and low sunny days, M-paint decreased the average peak temperature by 2 °C and 1 °C, respectively, in comparison to S-paint.	[157]
Jeong et al. (2016)	RT31 (Rubitherm)	Melamine.	MPCM.	Two types of paints (from JB Paints, South Korea) were tested: 1. Hydrophilic Paint: Acrylic emulsion resin; 2. Hydrophobic Paint: Urethane waterproof agent.	Hydrophilic paints showed better compatibility with the MPCM compared to hydrophobic paints. PCM paint reduced peak temperature by ~1.5 °C	[158]
Naikwadi et al. (2022)	n-Nonadecane	PMMA-co-BA-co-MAA.	MPCM.	Acrylic-based outdoor building paint made by blending PMMA-co-BA-co-MAA acrylic binder with different MPCM emulsion concentrations (50 wt% and 60 wt%).	It took three, four, and five hours, respectively, for the paint without microcapsules, A1 paint, and A2 paint to achieve equilibrium temperature.	[162]
Han et al. (2017)	OPCM	PMMA.	core: shell 7:3.	Paint coating (2–3 mm thick).	The room with the MPCM coating showed a reduction in temperature amplitude by 5–6 °C. The MPCM coating reduced the start-up time of the air conditioner and led to a 26% reduction in energy consumption during the peak temperature period.	[159]

**Table 13:** Application of gypsum board in buildings using different MPCM.

Authors	Building Simulation/ Experimental	Numerical Method/ Experimental	PCM	Encapsulation	Study	Observations	Ref.
Xu et al. (2024)	EnergyPlus version 24.1.0, TRNSYS	An R code was developed to facilitate numerical simulations and data analysis, incorporating libraries for computational fluid dynamics and heat transfer analysis.	Comfortboard23, a commercial gypsum board that incorporates MPCM (paraffin wax).	Encapsulated in microscopic PMMA shells before being mixed with gypsum.	Evaluates the thermal performance and energy-saving potential of Comfortboard23, a commercial gypsum board infused with MPCM.	The investigation demonstrated that Comfortboard23, a MPCM-integrated gypsum board, has significant potential to improve TES, regulate indoor temperatures, and reduce energy consumption in buildings.	[163]
Wadee et al. (2023)	EnergyPlus	Experimental data used in Design Builder simulations.	RT18HC, RT22HC, RT25HC; paraffin-based PCM	Vacuum impregnation into aerated granules; coated with sodium silicate.	The study developed gypsum plaster specimens by mixing gypsum with PCM-loaded granules at 10% to 50% by volume, maintaining a water-to-plaster ratio of 3:5. The specimens were cured for 28 days at 20 °C and 40% RH before testing.	The thermal conductivity of PCM-gypsum plaster increased with higher PCM content. The RT25HC plaster with 50% PCM had a thermal conductivity higher (0.45 W·m^−1^·K^−1^) than control (0.35 W·m^−1^·K^−1^).	[164]
Kumar et al. (2023)	Real outdoor testing in cubicles	Experimental data collection using thermocouples and data loggers.	Lauric acid, a natural PCM; T_m_ of 43.5 °C; LH 164 J·g^−1^	SSPCM; PCM absorbed into zeolite; graphite added for thermal conductivity.	G-SSPCM-5 reduced indoor peak temp by 3.0 °C (roof) and 4.54 °C (south wall); time delay up to 200 min.	The G-SSPCM-5 board (5 wt% graphite) effectively reduced indoor peak temperatures and delayed heat transfer, while excessive graphite (10 wt%) caused faster PCM charging and higher temperatures, emphasizing the need for optimal graphite content.	[165]
Nowak et al. (2023)	Experimental testing of PCM-integrated gypsum plasterboard	Use different experimental methods such as dynamic heat flow metre apparatus (DHFMA) and DSC.	Micronal**^®^** SmartBoard™ 23.	MPCM particle size 2–20 µm.	Estimate the thermal performance of MPCM-integrated gypsum plasterboard using dynamic thermal property measurements.	The MPCM-integrated gypsum board shows phase change between 16 and 26 °C with a peak heat capacity of 8772 J·kg^−1^·K^−1^ and exhibits hysteresis, with solidification at lower temperatures than melting. The DHFMA method effectively tests large PCM samples but is time intensive.	[166]
Gao et al. (2024)	Experimental testing of MPCM-integrated gypsum boards under controlled radiant conditions.	Experimental investigations.	Eutectic natural MPCM consisting of lauric acid (LA), myristic acid (MA), and stearic acid (SA).	The MPCM was stabilized using expanded graphite (EG) and diatomite (DE) to prevent leakage and enhance thermal conductivity.	To investigate the thermoregulation effect of MPCM-integrated gypsum boards with different structures under various radiant conditions.	The phase change gypsum board with the MPCM layer on the interior side exhibited the best thermal performance, with the longest duration of thermal comfort.	[160]
Bake et al. (2021)	Experimental characterization of PCM-integrated gypsum plasterboards	Experimental measurements and physical characterization techniques.	MPCM paraffin wax (MICRONAL**^®^** DS 5040X).	The MPCM used a highly cross-linked polymethylmethacrylate (PMMA) polymer shell.	Develop and characterize MPCM-gypsum plasterboards for energy-efficient buildings.	The 15% MPCM plasterboard showed the best thermal performance, with the lowest thermal conductivity and effective heat storage capabilities.	[168]
Errebai et al. (2021)	Experimental measurements and thermophysical properties of MPCM mixed with gypsum.	Measuring thermophysical properties: thermal conductivity, density, specific heat capacity, volumetric heat capacity, thermal diffusivity, and thermal effusivity.	Micronal DS 5001 Micronal DS 5040	MPCM with PMMA as shell.	To determine the optimum mass percentage of MPCM mixed with gypsum for improved LHS and to identify the temperature range where MPCM is most effective.	The study demonstrates that incorporating 20–30% MPCM in gypsum boards enhances thermal inertia, improving building comfort and reducing energy consumption by efficiently storing and releasing LH, thereby lowering greenhouse gas emissions.	[169]
Bravo et al. (2020)	EnergyPlus™	The simulation incorporates experimentally measured thermophysical properties (thermal conductivity, heat capacity, and enthalpy) of the MPCM-modified gypsum boards.	A paraffin-based MPCM was used, specifically MicroKaps 28.	MPCM with melamine-formaldehyde shell.	To evaluate the thermal behaviour of gypsum boards modified with MPCM in a test enclosure under the climatic conditions of Santiago de Chile.	The EnergyPlus™ simulation predicted a reduction in the maximum internal temperature of the enclosure by up to 1.5 °C when using MPCM-modified gypsum boards.	[170]
Gencel et al. (2023)	Experimental testing of the thermal and light-transmitting properties of gypsum composites.	Experimental investigations.	MPCM was used, specifically Nextek 18D.	MPCM with polymeric shell.	To develop a light-transmitting gypsum composite with integrated MPCM for improved TES and lighting efficiency in buildings.	15% MPCM provided the best TES performance, with a LH capacity of 19.2 J·g^−1^ and T_m_ 17.76 °C.	[171]
Zhou et al. (2024)	To validate the experimental data, it was used a numerical simulation that is Finite Element Method (FEM).	Finite Element Method (FEM).	MPCM, specifically NEXTEK 24.	MPCM	The study focused on the thermal behaviour of a gypsum board incorporated with MPCM in a subarctic climate, focusing on temperature regulation and energy storage.	In comparison to pure gypsum board, the MPCM-gypsum composite decreased indoor temperature swings by a maximum of 0.7 °C.	[161]

**Table 14:** Application of insulating materials in buildings using different MPCM.

Authors	Building Simulation/ Experimental	Numerical Method/ Experimental	PCM	Encapsulation	Study	Observations	Ref.
Hamooleh et al. (2024)	EnergyPlus, Residential building, 2 stories, 4 cities in Iran	Response Surface Methodology, 216 DOE points, R^2^ close to 1.	BioPCMDSCM27021, BioPCMDSCM51023, BioPCMDSCM91029.	PCM are integrated into the building envelope (walls and roof).	Reduction in energy used for heating and cooling at home. It was developed on four cities in Iran (Tehran, Bandar Abbas, Tabriz and Rasht) with different climate conditions. The optimization process was performed using the Response Surface Methodology and for the energy performance of the buildings is used EnergyPlus for simulations.	The best heating setpoint is ~20 °C, and cooling is ~28 °C (25 °C for hot climates), with PUR insulation and BioPCMDSCM27021. Hot regions (Bandar Abbas) had significant energy saving, and cold climates (Tabriz) experienced the greatest advances in comfort.	[172]
Arumugam et al. (2024)	DesignBuilder 7.3 (with EnergyPlus as the simulation engine).	Response Surface Methodology.	BioPCM**^®^**Q27 BioPCM**^®^**Q29.	BioPCM was integrated into walls and roof in layers with pouches for filling PCM.	Improve thermal comfort and reduce energy consumption in office buildings using BioPCM, insulation, and natural ventilation.	In comparison to conventional air-conditioned buildings, the optimized building model that combined BioPCM (30 mm) and insulation (50 mm) reduced energy consumption by 61% (from 122 kWh to 47 kWh monthly) while achieving tolerable thermal comfort with an average operating temperature of 26.6 °C.	[175]
Abbas et al. (2021)	FORTRAN, Two-room experimental model, Al-Diwaniyah, Iraq.	Finite Volume Method, Enthalpy Method, Navier–Stokes Equations, 0.5 s time step.	Paraffin wax, T_m_: 40 °C, Heat storage: 174 kJ·kg^−1^.	ePCM in aluminum tubes (29 mm × 70 mm × 0.4 mm).	ePCM capsules installed in hollow bricks walls in Iraq was evaluated as heat insulation in actual outdoor settings. To examine the effects of PCM-treated and non-PCM walls on heat transmission, thermal lag and indoor temperature stability, it presents a plug-and-play wall system. A 3D finite volume model using FORTRAN was created to validate experimental results.	By reducing temperature variations by 23.84%, delaying heat transfer by 2 h, and lowering indoor temperatures by 4.7 °C, ePCM integration improved thermal comfort. ePCM requires improved brick integration, according to the numerical model, which displayed a variation in less than 4%.	[176]
Zhilyaev et al. (2023)	OpenLCA: LCA and life cycle costing assessment. Matlab: Employed to investigate trade-offs between material qualities in multi-objective optimization (MOO). FEM: Applied electrical and thermal conductivity modelling.	Numerical method used: FEM. Lewis-Nielsen Model and MOO	MPCM	MPCM	To assess the new insulation material NRG-Foam, which is based on cementitious foam doped with MPCM, in terms of its economic, functional and environmental performance.	A large proportion of environmental impacts up to 95% of all impacts, are caused by the manufacture of MPCM.	[177]

**Table 15:** Application of building envelope retrofits in buildings using different MPCM/PCM.

Authors	Building Simulation/ Experimental	Numerical Method/ Experimental	PCM	Encapsulation	Study	Observations	Ref.
Berrocal et al. (2021)	DesignBuilder v6.1.6.011 (based on EnergyPlus).	Dynamic energy simulation using DesignBuilder.	Paraffin wax P56-58, RT21 (PCMC21), PCM1, Dupont Energain and BioPCM.	MPCM, ePCM, and form-stable PCM.	Analyses how effectively PCM work as a passive method to reduce energy consumption and increase thermal comfort in buildings, particularly in Panama City’s tropical climate.	The result shows that Dupont Energain provided the highest energy saving 22.93% in Panama’s tropical climate, while Cera Paraffin P56-58 was not so effective due to its high melting point. PCM significantly reduced indoor temperature fluctuations, and PCM1 shows the best thermal stability.	[180]
Al-Yasiri et al. (2023)	EnergyPlus (with DesignBuilder as the interface).	Conduction Finite Difference is utsed to simulate heat transport, including the PCM phase shift, the	Paraffin wax	PCM was incorporated straight into the building envelope (walls and roof).	Focus on the combination of PCM and EPS thermal insulation to improve the thermal performance of a building envelope in harsh summer conditions.	Adding 1 cm of EPS insulation to PCM greatly enhanced thermal performance, lowering indoor temperatures by up to 7.6 °C and postponing peak temperatures by 2.2 h. Additionally, heat gain was reduced by 80.9%.	[173]
Benachir et al. (2023)	EnergyPlus and TRNSYS.	Heat equation and convective flows.	PCM.	**-**	Using PCM and passive cooling methods assess the energy efficiency of buildings, especially considering Morocco’s environment. It evaluates passive methods on walls and roofs.	PCM integration in roof and walls leads to 10–20% reduction in air conditioning demands and improved thermal comfort with lower HVAC dependency.	[181]
Khan et al. (2022)	EnergyPlus	Conduction Finite Difference algorithm	CrodaTherm24—bio-based OPCM	PCM embedded in building envelopes (roofs and walls)	The research centres on numerical simulation using EnergyPlus to evaluate the thermal and economic benefits of using PCM for the reduction in cooling and heating energy consumption.	EnergyPlus simulations are used in this study to investigate PCM integration in residential buildings in Pakistan. The best PCM was CrodaTherm24 (24 °C, 183 kJ·kg^−1^), which decreased energy utilization by 49.6%.	[174]
Mousazadeh et al. (2024)	DesignBuilder	Finite Difference Method that is issued for transient heat transfer simulations. Also, the use of heat transfer equations.	RT18HC (Rubitherm)—OPCM that is based of paraffin	PCM was directly incorporated into the walls and had an additional integration compared with nano-paint coatings for energy efficiency.	To assess the effects of PCM (RT18HC), nanopaints, and traditional insulation on energy efficiency, economic. viability and sustainability, the investigation focuses on a multi-story hotel in Mashhad, Iran.	PCM reduced energy consumption by 6% and EUI by 5.6%, outperforming nano-paints (1.8% EUI reduction). It was most effective in lowering cooling loads in summer and heating demand in winter.	[182]
Beiranvand et al. (2021)	DesignBuilder (based on EnergyPlus dynamic simulation engine).	Heat equation and thermal load calculation	BioPCM M182.	PCM is directly incorporated into building envelopes (south wall) in layers	This study investigates how PCM-enhanced building envelopes affect energy consumption decrease in commercial buildings.	The greatest energy savings are achieved when PCM layers with varying T_m_ are combined (3.8% reduction compared to the non-PCM scenario).	[183]
Hagenau et al. (2020)	SketchUp Pro, Open Studio and EnergyPlus	Heat equation and thermal load calculation	17 different PCM were evaluated, with T_M_ ranging from 18 °C to 26 °C	PCM is directly embedded into the building envelope (walls and roofs).	To investigate the effect of PCM-enhanced building envelopes on energy consumption reduction and indoor thermal comfort in Danish buildings.	Multiple PCM layers with different T_m_ provides the best energy savings (up to 15% reduction in energy consumption). PCM reduces energy consumption in climates with large T variations.	[184]
Kulumkanov et al. (2024)	EnergyPlus	Conduction Finite Difference algorithm in EnergyPlus.	BioPCM (PCM18-PCM30)	The thickness of the PCM is 12.5 mm layer on exterior walls and roof. Direct PCM incorporation into the walls and roof layers.	Under climate change scenarios: potential energy efficiency and environmental sustainability of PCM integration in building envelopes (year 2095).	Building energy demand is successfully decreased using PCM, with annual savings of up to 12.9%. One advantage for the environment is lower CO_2_ emissions, which promotes sustainable building practices.	[185]
Laasri et al. (2024)	EnergyPlus	Conduction Finite Difference algorithm in EnergyPlus.	RT28HC (Rubitherm)	Macro-encapsulated in aluminum panels	The study examines the performance of ePCM (RT28HC) in building envelope for semi-arid climates, particularly in Morocco.	The incorporation of PCM improves temperature stability and reduces energy demand; yet, subcooling reduces efficiency. The 25.3% of energy savings, with higher impact in heating 37.2% than a cooling 20.06%.	[186]

**Table 16:** MPCM studies: products, properties, and regions.

	Study	PCM/MPCM Product/Cement Product	T_m_ (°C)/LH (kJ·kg^−1^)	Region/Climate	Application
CEMENT	Soudian et al. [113]	Organic MPCM Nextek**^®^**/Ordinary Portland cement type I, lime, and sand was used as the aggregate.	18, 24, and 28 °C/190, 170–178, 180–190 kJ·kg^−1^	Toronto/Hot urban climates.	Thermo-optical cement plaster with PCM and TC for UHI mitigation.
	Bre et al. [118]	Micronal**^®^** DS 5038 X (BASF—powder)—Paraffin wax/cement-based layer—the mixture with w/c = 0.4 and 20% of MPCM volume fraction.	-	Sofia (Bulgaria-EU)/Cool—Humid.	Optimized PCM cement panel (EnergyPlus—NSGA)—MPCM is added to the inside of the existing walls.
	Gencel et al. [119]	CEM II/B-L 42.5R-Portland Calcareous Cement (Cimsa eco white cement)/MPCM Nextek**^®^** 18D Microtek.	~18 °C	Turkey.	Light-transmitting MPCM cementitious composite.
MORTAR	Salihi et al. [120]	CaCO_3_—shelled MPCM with Paraffin RT-26/Mortar (45 Ordinary Portland cement (CimarPro 45**^®^**)).	~26 °C	Benguerir city, Morocco/Semiarid climate.	Mortar panels for thermal regulation and passive cooling (outdoor testing).
	Gbekou et al. [123]	M0 (cement mortar wall) and M15D (wall made from the reference mortar with the addition of MPCM bio-based) CrodaTherm™ ME29D/Mortar include sand (fine aggregate), OPC (EXTREMAT**^®^** CEM I 52.5 N), CSA, and SP.	-	Mediterranean and oceanic climate.	Wall systems for energy-efficient building envelopes.
CONCRETE	Lajimi et al. [126]	MPCM Paraffin RT25.	26 °C	Tunisia—Sousse region (North Africa)—Hot Mediterranean/semi-arid.	PCM-enhanced concrete ceiling layer (PCC) (Ceiling composed of PCC layer placed on the exterior side, a layer of brick and concrete).
	Cabeza et al. [124]	Addition of 5 wt% MPCM Micronal DS 5001 into concrete.	26 °C	Spain (Lleida)/Summer weather conditions.	Full-scale PCM concrete cubicles; long-term durability.
WALL	Dardouri et al. [134]	the Infinite R™ PCM: PCM18, PCM21, PCM23, PCM25, and PCM29.	18 °C, 21 °C, 23 °C, 25 °C, and 29 °C/200 kJ·kg^−1^	Tunisia: - Sousse (lower semiarid), - Bizerte (lower humid) - Tabarka (Upper humid) - Tozeur (Saharian).	PCM in Mediterranean External Walls (PCM inside and outside).
	Babaharra et al. [135]	n-octadecane paraffin microencapsulated with a thin layer (≤ 0.1 μm ≤ e ≤ 0.2 μm) of PMMA.	28 °C	Moroccan/hot climate zone.	Multilayer wall with MPCM.
ROOF	Chen et al. [136]	SSPCM (no shell).	180 kJ·kg^−1^	China (Guangzhou, Kunming, Shanghai, Beijing and Harbin) and Europe (Rome/Italy, London (Britain) and Berlin (Germany). 1. Kunming (LH + LC) = Mild climate, low heating and cooling demand. 2. Harbin, London, and Berlin (HH + LC) = Cold winters (high heating demand), mild summers (low cooling demand). 3. Guangzhou, Shanghi, Rome (LH + HC) = Hot summer (high cooling demand), mild winters (low heating demand) 4. Beijing (HH + HC) = Extreme climate: cold winters (high heating demand) and hot summer (high cooling demand).	PCM Roof + Cool Paint (added outside and inside of the concrete at the same time).
	Dardouri et al. [134]	Infinite R™ PCM.	18 °C, 21 °C, 23 °C, 25 °C, 29 °C/200 kJ·kg^−1^	Tunisia (Sousse, Bizerte, Tabarka and Tozeur)/Mediterranean.	South roofs (external/internal), optimizing a 40 mm double-layer system.
	Yu et al. [137]	SSPCM (85% paraffin + 15% HDPE).	34–36 °C/160.7 kJ·kg^−1^	Wuhan, China (hot climate).	Ventilation roof with night cooling.
	Luo et al. [139]	Paraffin wax.	28 °C	Hohhot, Harbin, Shanghai, Urumqi and Xining (China).	Paraffin-filled porous brick roofs, evaluated with multiple relaxation time lattice Boltzmann simulations.
	Jia et al. [140]	cetane, heptadecane, octadecane, salt hydrate, and gypsum board (PC—obtained by mixing phase change particles, water and dispersant into gypsum powder, pouring into mould, compacting and air drying).	231 kJ·kg^−1^, 200 kJ·kg^−1^, 241 kJ·kg^−1^, 281 kJ·kg^−1^ and 45 kJ·kg^−1^	Lanzhou, Kunming, Anda, Wuhan and Xiamen (China)	Prefabricated building roofs (external/internal).
WINDOW	Uribe et al. [138]	Paraffin RT25 (Liquid PCM).	25 °C	Santiago of Chile/semi-arid climate.	PCM-filled double-glazing window; 22% cooling and 45% heating savings.
	Ma et al. [142]	Solid–solid PCM + silica aerogel integration.	-	China/severe cold region.	Solid—solid PCM glazing ~18.2% annual heating-cooling energy savings.
	Chen-Pan et al. [145]	Paraffin MG29 and n-octadecane.	27–29 °C, 27.8 °C	Merida (hot) and Toluca (cold), Mexico	PCM-integrated in a window shutter as a passive system.
BRICK	Mahdaoui et al. [147]	n-nonadecane PCM (cylindrical capsules).	32 °C	Morocco (hot semiarid climate).	Cylindrical capsules incorporated into 12-hole hollow clay bricks; 16% PCM reduced heat-wave penetration by stabilizing the inner surface temperature at about 27.5 °C.
	Fraine et al. [152]	PCHCM (Phase Change Humidity Control Material) = MPCM + diatomite (filler).	28.1 °C	Algeria.	PCHCM filled into sintered hollow bricks; optimal location = 66% fill on interior side; up to 50% energy saving compared to EPS.
PAINT	Ghayedhosseini et al. [156]	Cool Paint − CP + TP20 PCM − TP23 PCM	220 kJ·kg^−1^, 230 kJ·kg^−1^	Shiraz city (Iran)—Hot and dry climate.	Combined and individual simulation using Cool Paint and PCM in high-rise office building. PCM in the outer and inner layer.
GYPSUM BOARD	Bravo et al. [170]	MPCM integrated gypsum board mPCM (MikroCaps28).	26.5 °C to 29.2 °C Numerical + experimental match; Seasonal test (September–November)	Santiago, Chile.	EnergyPlus-calibrated gypsum/MPCM board in test enclosure.
	Gencel et al. [171]	Nextek**^®^** MPCM (10–15 wt%) in light-transmitting gypsum.	~18 °C	Turkey (cloudy, sunny, and rainy weather condition).	Light-transmitting MPCM-gypsum composite.
INSULATING MATERIAL	Hamooleh et al. [172]	BioPCM DSCM27Q21/PUR as insulator.	-	Iran (Tehran, Bandar Abbas, Tabriz, and Rasht)/(4 climates).	PCM + insulation composite (PCM outside, middle, inside).
	Arumugam et al. [175]	BioPCM**^®^** Q27/Q29—Insulation material: XPS extruded polystyrene–HFC blowing.	210–250 J·g^−1^, 210–250 J·g^−1^	Chennai, India.	PCM + XPS insulation.
	Abbas et al. [176]	Paraffin was-based on PCM.	38–43 °C	Iraq (hot).	PCM-filled cavity insulation.
ENVELOPE	Berrocal et al. [180]	Paraffin Wax P56-58, RT21, PCM1, Dupont Energain, BioPCM.	Melting ranges depend on product (e.g., RT21 ≈ 21 °C; BioPCM varies). Energain showed best performance (22.93% energy saving).	Ciudad de Panamá.	PCM integrated in Buildings (passive strategy).
	Al-Yasiri et al. [173]	Paraffin wax PCM embedded in EPS insulation.	-	Hot-arid climate (Iraq).	Combined use of PCM and thermal insulation to improve the building thermal performance.
	Khan et al. [174]	15 different PCM (SP21EK,SP24E,SP25E2,A22H,A25H,RT21HC,RT22HC,RT25HC, PureTemp18, PureTemp20, PureTemp23, CrodaTherm19, CrodaTherm21, CrodaTherm24W, CrodaTherm24).	22, 24, 25, 22, 25, 21, 22, 25, 18, 20, 23, 19, 21, 23 and 24 °C/170, 180, 180, 216, 226, 190, 190, 230, 192, 171, 201, 175, 190, 184, and 183 kJ·kg^−1^	Pakistan (Islamabad, Karachi, Lahore, Pesh awar, Quetta).	PCM in walls/roof; thickness optimization study for building envelope.
	Mousazadeh et al. [182]	RT18HC PCM from paraffin (Rubitherm Company).	-	Iran (Mashhad).	PCM layer in wall/roof envelope components.
	Beiranvand et al. [183]	PCM21, PCM23, PCM25 (melting point 23 °C).	21, 23, 25 °C	Iran (Torbat Heydarieh)/dry climate with hot summer and cold winter.	Case study of an energy analysis and simulation of PCM-enhanced in building envelopes.
	Hagenau et al. [184]	17 different PCM products evaluated (RT26, RT25HC, PureTemp25, SP25E2, CrodaTherm24, RT24, SP24E, PureTemp23, RT22HC, SP21EK, CrodaTherm21, RT21HC, RT21, PureTemp20, CrodaTherm19, RT18HC and PureTemp18).	-	Denmark (Copenhagen, Aalborg, and Esbjerg).	PCM implementation in envelope—four case study buildings.

## Data Availability

The original contributions presented in this study are included in the article. Further inquiries can be directed to the corresponding author.

## Abbreviations

The following abbreviations are used in this manuscript in order of appearance:

PCM	Phase change materials
MPCM	Microencapsulated phase change materials
LCA	Life cycle assessment
EU	European Union
GHG	Greenhouse gases
IEA	International Energy Agency
HVAC	Heating, ventilation, and air conditioning
ES	Energy storage
RES	Renewable energy sources
ESS	Energy storage systems
TES	Thermal energy storage
SHS	Sensible heat storage
LHS	Latent heat storage
TCES	Thermochemical energy storage
LH	Latent heat
OPCM	Organic PCM
PEG	Polyethylene glycol
Tm	Melting temperature
IPCM	Inorganic PCM
EPCM	Eutectic PCM
NEPCM	Nanoencapsulation of PCM
ePCM	Macroencapsulation of PCM
PVC	Polyvinyl chloride
HDPE	High-density polyethylene
PE	Polyethylene
PP	Polypropylene
PMMA	Polymethylmethacrylate
SSPCM	Shape-stabilized PCM
PUR	Polyurethane
MF	Melamine-formaldehyde
UF	Urea-formaldehyde
PLA	Poly(lactic acid)
S-L	Solid–liquid
CFD	Computational fluid dynamics
UHI	Urban Heat Island
TC	Thermochromic
CNT	Carbon nanotubes
rGO	Reduced graphene oxide
FSPCM	Form-stable PCM
HEP	Hydrophobic expanded perlite
CA	Capric acid
PCC	Phase change concrete
CP	Cool Paint
S-S	Solid–solid
SPGW	Silica aerogel integration
NIR	Near-infrared
PTR	Peak temperature reduction
PCHCM	Phase change humidity control material
SEM	Scanning electron microscopy
DSC	Differential scanning calorimetry
MA	Myristic acid
SA	Stearic acid
FEM	Finite element method
MOO	Multi-objective optimization
DHFMA	Dynamic heat flow metre apparatus

## References

[1] Weart S.R. (2010). The idea of anthropogenic global climate change in the 20th century. WIREs Clim. Change.

[2] Bilgen S. (2014). Structure and environmental impact of global energy consumption. Renew. Sustain. Energy Rev..

[3] Amin A., Liu X.-H., Abbas Q., Hanif I., Vo X.V. (2021). Globalization, sustainable development, and variation in cost of power plant technologies: A perspective of developing economies. Environ. Sci. Pollut. Res..

[4] Shahzad S., Faheem M., Muqeet H.A., Waseem M. (2024). Charting the UK’s path to net zero emissions by 2050: Challenges, strategies, and future directions. IET Smart Grid.

[5] Apostu S.A., Panait M., Vasile V. (2022). The energy transition in Europe—A solution for net zero carbon?. Environ. Sci. Pollut. Res..

[6] Aste N., Del Pero C., Leonforte F. (2022). Toward Building Sector Energy Transition. Handbook of Energy Transitions.

[7] Abbasi K.R., Shahbaz M., Zhang J., Irfan M., Alvarado R. (2022). Analyze the environmental sustainability factors of China: The role of fossil fuel energy and renewable energy. Renew. Energy.

[8] Dincer I., Aydin M.I. (2023). New paradigms in sustainable energy systems with hydrogen. Energy Convers. Manag..

[9] Müller-Casseres E., Edelenbosch O.Y., Szklo A., Schaeffer R., van Vuuren D.P. (2021). Global futures of trade impacting the challenge to decarbonize the international shipping sector. Energy.

[10] Zastempowski M. (2023). Analysis and modeling of innovation factors to replace fossil fuels with renewable energy sources—Evidence from European Union enterprises. Renew. Sustain. Energy Rev..

[11] Lee J., Yoon Y.T., Lee G.-J. (2024). Renewable Energy Sources: From Non-Dispatchable to Dispatchable, and Their Application for Power System Carbon Neutrality Considering System Reliability. J. Electr. Eng. Technol..

[12] Xie Y., Wu X., Hou Z., Li Z., Luo J., Lüddeke C.T., Huang L., Wu L., Liao J. (2023). Gleaning insights from German energy transition and large-scale underground energy storage for China’s carbon neutrality. Int. J. Min. Sci. Technol..

[13] De Carne G., Maroufi S.M., Beiranvand H., De Angelis V., D’Arco S., Gevorgian V., Waczowicz S., Mather B., Liserre M., Hagenmeyer V. (2024). The role of energy storage systems for a secure energy supply: A comprehensive review of system needs and technology solutions. Electr. Power Syst. Res..

[14] Rana M.M., Uddin M., Sarkar M.R., Meraj S.T., Shafiullah G.M., Muyeen S.M., Islam M.A., Jamal T. (2023). Applications of energy storage systems in power grids with and without renewable energy integration—A comprehensive review. J. Energy Storage.

[15] Zhang S., Ocłoń P., Klemeš J.J., Michorczyk P., Pielichowska K., Pielichowski K. (2022). Renewable energy systems for building heating, cooling and electricity production with thermal energy storage. Renew. Sustain. Energy Rev..

[16] Manar G., Shalaby M., Bakar M.S., Parveez B., Najeeb M.I., Hassan M.K., Al-Sowayan S., Alawad M.A. (2025). Bio-Based Composites with Encapsulated Phase Change Materials for Sustainable Thermal Energy Storage: A Review. Polymers.

[17] Alva G., Lin Y., Fang G. (2018). An overview of thermal energy storage systems. Energy.

[18] Heier J., Bales C., Martin V. (2015). Combining thermal energy storage with buildings–a review. Renew. Sustain. Energy Rev..

[19] Morovat N., Athienitis A.K., Candanedo J.A., Dermardiros V. (2019). Simulation and performance analysis of an active PCM-heat exchanger intended for building operation optimization. Energy Build..

[20] Patel J.H., Qureshi M.N., Darji P.H. (2018). Experimental analysis of thermal energy storage by phase change material system for cooling and heating applications. Mater. Today Proc..

[21] Lane G., Lee M., Collins J., Aubrecht D., Sperling R., Solomon L., Ha J., Yi G., Weitz D., Manoharan V. (1986). Synchronized reinjection and coalescence of droplets in microfluidics. Lab A Chip.

[22] Elias C.N., Stathopoulos V.N. (2019). A comprehensive review of recent advances in materials aspects of phase change materials in thermal energy storage. Energy Procedia.

[23] Jouhara H., Żabnieńska-Góra A., Khordehgah N., Ahmad D., Lipinski T. (2020). Latent thermal energy storage technologies and applications: A review. Int. J. Thermofluids.

[24] Prasad D., Senthilkumar R., Lakshmanarao G., Krishnan S., Prasad B. (2019). A critical review on thermal energy storage materials and systems for solar applications. Aims Energy.

[25] Nistor C.L., Gifu I.C., Anghel E.M., Ianchis R., Cirstea C.-D., Nicolae C.A., Gabor A.R., Atkinson I., Petcu C. (2023). Novel PEG6000–Silica-MWCNTs Shape-Stabilized Composite Phase-Change Materials (ssCPCMs) for Thermal-Energy Storage. Polymers.

[26] Bianchi M., Valentini F., Fredi G., Dorigato A., Pegoretti A. (2022). Thermo-Mechanical Behavior of Novel EPDM Foams Containing a Phase Change Material for Thermal Energy Storage Applications. Polymers.

[27] Hekimoğlu G., Sarı A. (2022). A review on phase change materials (PCMs) for thermal energy storage implementations. Mater. Today Proc..

[28] Akeiber H., Nejat P., Majid M.Z.A., Wahid M.A., Jomehzadeh F., Famileh I.Z., Calautit J.K., Hughes B.R., Zaki S.A. (2016). A review on phase change material (PCM) for sustainable passive cooling in building envelopes. Renew. Sustain. Energy Rev..

[29] Magendran S.S., Khan F.S.A., Mubarak N., Vaka M., Walvekar R., Khalid M., Abdullah E., Nizamuddin S., Karri R.R. (2019). Synthesis of organic phase change materials (PCM) for energy storage applications: A review. Nano-Struct. Nano-Objects.

[30] Pandey A., Hossain M., Tyagi V., Abd Rahim N., Jeyraj A., Selvaraj L., Sari A. (2018). Novel approaches and recent developments on potential applications of phase change materials in solar energy. Renew. Sustain. Energy Rev..

[31] Rathore P.K.S., Sikarwar B.S. (2024). Thermal energy storage using phase change material for solar thermal technologies: A sustainable and efficient approach. Sol. Energy Mater. Sol. Cells.

[32] Chandel S., Agarwal T. (2017). Review of current state of research on energy storage, toxicity, health hazards and commercialization of phase changing materials. Renew. Sustain. Energy Rev..

[33] Faraj K., Khaled M., Faraj J., Hachem F., Castelain C. (2020). Phase change material thermal energy storage systems for cooling applications in buildings: A review. Renew. Sustain. Energy Rev..

[34] Hossain M.T., Shahid M.A., Ali M.Y., Saha S., Jamal M.S.I., Habib A. (2023). Fabrications, classifications, and environmental impact of PCM-incorporated textiles: Current state and future outlook. ACS Omega.

[35] Romdhane S.B., Amamou A., Khalifa R.B., Said N.M., Younsi Z., Jemni A. (2020). A review on thermal energy storage using phase change materials in passive building applications. J. Build. Eng..

[36] Galvagnini F., Dorigato A., Fambri L., Fredi G., Pegoretti A. (2021). Thermophysical Properties of Multifunctional Syntactic Foams Containing Phase Change Microcapsules for Thermal Energy Storage. Polymers.

[37] Peng H., Wang J., Zhang X., Ma J., Shen T., Li S., Dong B. (2021). A review on synthesis, characterization and application of nanoencapsulated phase change materials for thermal energy storage systems. Appl. Therm. Eng..

[38] Giro-Paloma J., Martínez M., Cabeza L.F., Fernández A.I. (2016). Types, methods, techniques, and applications for microencapsulated phase change materials (MPCM): A review. Renew. Sustain. Energy Rev..

[39] Giro-Paloma J., Alkan C., Chimenos J.M., Fernández A.I. (2017). Comparison of microencapsulated phase change materials prepared at laboratory containing the same core and different shell material. Appl. Sci..

[40] Jamróz E., Kulawik P., Kopel P. (2019). The Effect of Nanofillers on the Functional Properties of Biopolymer-Based Films: A Review. Polymers.

[41] Nazir H., Batool M., Osorio F.J.B., Isaza-Ruiz M., Xu X., Vignarooban K., Phelan P., Kannan A.M. (2019). Recent developments in phase change materials for energy storage applications: A review. Int. J. Heat Mass Transf..

[42] Ghalambaz S. (2024). A Scientometric Study of Nano Encapsulated Phase Change Material (NEPCM): Trends and Categories. J. Nanofluids.

[43] Shao W., Nayak M., Ali R., Nazari S., Chamkha A.J. (2024). Simultaneous numerical examination of thermal and entropy characteristics of Al_2_O_3_–H_2_O nanofluid within a porous diamond-shaped container with a⊥-shaped obstacle. Case Stud. Therm. Eng..

[44] Liu C., Rao Z., Zhao J., Huo Y., Li Y. (2015). Review on nanoencapsulated phase change materials: Preparation, characterization and heat transfer enhancement. Nano Energy.

[45] Nikpourian H., Bahramian A.R., Abdollahi M. (2020). On the thermal performance of a novel PCM nanocapsule: The effect of core/shell. Renew. Energy.

[46] Zhang N., Yuan Y. (2020). Synthesis and thermal properties of nanoencapsulation of paraffin as phase change material for latent heat thermal energy storage. Energy Built Environ..

[47] Zhang Z., Wang Y., Xu S., Yu Y., Hussain A., Shen Y., Guo S. (2017). Photothermal gold nanocages filled with temperature sensitive tetradecanol and encapsulated with glutathione responsive polycurcumin for controlled DOX delivery to maximize anti-MDR tumor effects. J. Mater. Chem. B.

[48] Pielichowska K., Pielichowski K. (2014). Phase change materials for thermal energy storage. Prog. Mater Sci..

[49] Aftab W., Huang X., Wu W., Liang Z., Mahmood A., Zou R. (2018). Nanoconfined phase change materials for thermal energy applications. Energy Environ. Sci..

[50] Cabeza L.F., Castell A., Barreneche C.d., De Gracia A., Fernández A. (2011). Materials used as PCM in thermal energy storage in buildings: A review. Renew. Sustain. Energy Rev..

[51] Milián Y.E., Gutiérrez A., Grageda M., Ushak S. (2017). A review on encapsulation techniques for inorganic phase change materials and the influence on their thermophysical properties. Renew. Sustain. Energy Rev..

[52] Ferrer G., Solé A., Barreneche C., Martorell I., Cabeza L.F. (2015). Review on the methodology used in thermal stability characterization of phase change materials. Renew. Sustain. Energy Rev..

[53] Panchabikesan K., Vincent A.A.R., Ding Y., Ramalingam V. (2018). Enhancement in free cooling potential through PCM based storage system integrated with direct evaporative cooling (DEC) unit. Energy.

[54] Mohaine S., Feliu J., Grondin F., Karkri M., Loukili A. (2016). Multiscale modelling for the thermal creep analysis of PCM concrete. Energy Build..

[55] Zhu L., Yang Y., Chen S., Sun Y. (2018). Numerical study on the thermal performance of lightweight temporary building integrated with phase change materials. Appl. Therm. Eng..

[56] Lee K.O., Medina M.A., Sun X. (2015). On the use of plug-and-play walls (PPW) for evaluating thermal enhancement technologies for building enclosures: Evaluation of a thin phase change material (PCM) layer. Energy Build..

[57] Peng G., Dou G., Hu Y., Sun Y., Chen Z. (2020). Phase change material (PCM) microcapsules for thermal energy storage. Adv. Polym. Tech..

[58] Pielichowska K., Nowicka-Dunal K., Pielichowski K. (2024). Bio-Based Polymers for Environmentally Friendly Phase Change Materials. Polymers.

[59] Maldonado-Alameda A., Lacasta A., Giro-Paloma J., Chimenos J., Haurie L., Formosa J. (2017). Magnesium phosphate cements formulated with low grade magnesium oxide incorporating phase change materials for thermal energy storage. Constr. Build. Mater..

[60] Jyothi S.S., Seethadevi A., Prabha K.S., Muthuprasanna P., Pavitra P. (2012). Microencapsulation: A review. Int. J. Pharm. Biol. Sci..

[61] Giro-Paloma J., Oncins G., Barreneche C., Martínez M., Fernández A.I., Cabeza L.F. (2013). Physico-chemical and mechanical properties of microencapsulated phase change material. Appl. Energy.

[62] Giro-Paloma J., Martínez M., Fernández A.I., Cabeza L.F. (2019). Microencapsulation of Phase Change Materials. Applications of Encapsulation and Controlled Release.

[63] Fleischer A.S. (2015). Thermal Energy Storage Using Phase Change Materials: Fundamentals and Applications.

[64] Yang D., Tu S., Chen J., Zhang H., Chen W., Hu D., Lin J. (2023). Phase Change Composite Microcapsules with Low-Dimensional Thermally Conductive Nanofillers: Preparation, Performance, and Applications. Polymers.

[65] Ferreira M.P.S., Gonçalves A.S., Antunes J.C., Bessa J., Cunha F., Fangueiro R. (2024). Fibrous Structures: An Overview of Their Responsiveness to External Stimuli towards Intended Application. Polymers.

[66] Bidiyasar R., Kumar R., Jakhar N. (2024). A critical review of polymer support-based shape-stabilized phase change materials for thermal energy storage applications. Energy Storage.

[67] Jiao K., Lu L., Zhao L., Wang G. (2024). Towards Passive Building Thermal Regulation: A State-of-the-Art Review on Recent Progress of PCM-Integrated Building Envelopes. Sustainability.

[68] Graziano A., Jaffer S., Sain M. (2018). Review on modification strategies of polyethylene/polypropylene immiscible thermoplastic polymer blends for enhancing their mechanical behavior. J. Elastomers Plast..

[69] Zafar M.S. (2020). Prosthodontic Applications of Polymethyl Methacrylate (PMMA): An Update. Polymers.

[70] Santana J.S., Cardoso E.S., Triboni E.R., Politi M.J. (2021). Polyureas Versatile Polymers for New Academic and Technological Applications. Polymers.

[71] Abubakar A.M., Yan K., Huang J., Demirelli K. (2025). A review on shell materials based on synthetic polymers for Micro-Encapsulated phase change materials(MEPCMs). J. Polym. Res..

[72] Nasr Y., El Zakhem H., Hamami A.E., El Bachawati M., Belarbi R. (2023). Comprehensive Review of Innovative Materials for Sustainable Buildings’ Energy Performance. Energies.

[73] Patil J.R., Mahanwar P.A., Sundaramoorthy E., Mundhe G.S. (2023). A review of the thermal storage of phase change material, morphology, synthesis methods, characterization, and applications of microencapsulated phase change material. J. Polym. Eng..

[74] Radouane N. (2022). A Comprehensive Review of Composite Phase Change Materials (cPCMs) for Thermal Management Applications, Including Manufacturing Processes, Performance, and Applications. Energies.

[75] Zhou D., Zhao C.Y., Tian Y. (2012). Review on thermal energy storage with phase change materials (PCMs) in building applications. Appl. Energy.

[76] Nandi A., Biswas N., Datta A., Manna N.K., Mandal D.K., Biswas S. (2025). A comprehensive review on enhanced phase change materials (PCMs) for high-performance thermal energy storage: Progress, challenges, and future perspectives. J. Therm. Anal. Calorim..

[77] Musa A.A., Bello A., Adams S.M., Onwualu A.P., Anye V.C., Bello K.A., Obianyo I.I. (2025). Nano-Enhanced Polymer Composite Materials: A Review of Current Advancements and Challenges. Polymers.

[78] Li L., Sun W., Gómez-Zamorano L.Y., Liu Z., Zhang W., Ma H. (2025). From Research Trend to Performance Prediction: Metaheuristic-Driven Machine Learning Optimization for Cement Pastes Containing Bio-Based Phase Change Materials. Polymers.

[79] Vera-Rivera D., Neira-Viñas M., Freixa-Arumí N., Formosa J., Giro-Paloma J. (2026). Microcapsules with thermal, mechanical, and chemical stability from polystyrene waste to contain phase change materials. J. Energy Storage.

[80] Yee M.S.-L., Hii L.-W., Looi C.K., Lim W.-M., Wong S.-F., Kok Y.-Y., Tan B.-K., Wong C.-Y., Leong C.-O. (2021). Impact of microplastics and nanoplastics on human health. Nanomaterials.

[81] Curran M.A. (2013). Life cycle assessment: A review of the methodology and its application to sustainability. Curr. Opin. Chem. Eng..

[82] Beltrami A. (2016). Trnsys Integrated Modeling Support Tool for a Fast Building-Plant System Design. Ph.D. Thesis.

[83] Meiers J., Frey G. (2025). Interfacing TRNSYS with MATLAB for Building Energy System Optimization. J. Build. Pathol. Rehabil..

[84] Moler C., Little J. (2020). A history of MATLAB. Proc. ACM Program. Lang..

[85] Matsson J.E. (2022). An Introduction to ANSYS Fluent 2022.

[86] Ibanez M., Lázaro A., Zalba B., Cabeza L.F. (2005). An approach to the simulation of PCMs in building applications using TRNSYS. Appl. Therm. Eng..

[87] Anter A.G., Sultan A.A., Hegazi A., El Bouz M. (2023). Thermal performance and energy saving using phase change materials (PCM) integrated in building walls. J. Energy Storage.

[88] Anupam B., Sahoo U.C., Rath P., Bhattacharya A. (2023). Thermal performance assessment of PCM incorporated cool concrete pavements using numerical analysis. Int. J. Pavement Eng..

[89] Soodmand A.M., Azimi B., Nejatbakhsh S., Pourpasha H., Farshchi M.E., Aghdasinia H., Mohammadpourfard M., Zeinali Heris S. (2023). A comprehensive review of computational fluid dynamics simulation studies in phase change materials: Applications, materials, and geometries. J. Therm. Anal. Calorim..

[90] Memon S.A., Cui H., Lo T.Y., Li Q. (2015). Development of structural–functional integrated concrete with macro-encapsulated PCM for thermal energy storage. Appl. Energy.

[91] Rashid K., Haq E.U., Kamran M.S., Munir N., Shahid A., Hanif I. (2019). Experimental and finite element analysis on thermal conductivity of burnt clay bricks reinforced with fibers. Constr. Build. Mater..

[92] Kheirabadi A.C., Groulx D. (2015). Simulating phase change heat transfer using comsol and fluent: Effect of the mushy-zone constant. Comput. Therm. Sci. Int. J..

[93] Jegan J., Anitha P., Logaraja R. (2025). Comprehensive study on thermal properties and application of phase change materials in construction. J. Build. Pathol. Rehabil..

[94] Kyriaki E., Konstantinidou C., Giama E., Papadopoulos A.M. (2018). Life cycle analysis (LCA) and life cycle cost analysis (LCCA) of phase change materials (PCM) for thermal applications: A review. Int. J. Energy Res..

[95] Nienborg B., Gschwander S., Munz G., Fröhlich D., Helling T., Horn R., Weinläder H., Klinker F., Schossig P. (2018). Life Cycle Assessment of thermal energy storage materials and components. Energy Procedia.

[96] Liang R., Ma H., Wang P., Zhao L. (2024). The applications of building information modeling in the life-cycle of green buildings: A comprehensive review. Sci. Technol. Built Environ..

[97] Arasteh H., Maref W., Saber H.H. (2023). Energy and thermal performance analysis of PCM-Incorporated glazing units combined with passive and active techniques: A review study. Energies.

[98] Abdellatef Y., Kavgic M. (2020). Thermal, microstructural and numerical analysis of hempcrete-microencapsulated phase change material composites. Appl. Therm. Eng..

[99] Dora S., Kuznik F., Mini K. (2025). A novel PCM-based foam concrete for heat transfer in buildings-Experimental developments and simulation modelling. J. Energy Storage.

[100] Zhang Y., Long G., Yang K., Lv P., An J., Zhu H., Liao Z., Mei W. (2025). A comprehensive investigation on the properties of phosphogypsum-based insulation mortar containing phase change material. Constr. Build. Mater..

[101] Torlaklı H., Ustaoğlu A., Yesilata B., Gencel O., Hekimoğlu G., Sarı A., Erdoğmuş E. (2025). A comparative study on PCM-impregnated shape-stable expanded vermiculite used in bricks for stabilizing indoor temperature and enhancing energy efficiency of buildings. Energy Build..

[102] Prajapati R.B., Rathod M.K., Banerjee J. (2025). Evaluating Thermal Performance of PCM-Integrated Roofs: A Numerical Study on Temperature Regulation Across Different Roof Types. Energy Storage.

[103] Achaku R., Li L., Chen Y. (2025). An experimental investigation of phase change material (PCM)-enhanced cavity walls with integrated windows in office buildings: Optimising energy savings. Sustain. Energy Technol. Assess..

[104] El Majd A., Sair S., Ait Ousaleh H., Moulakhnif K., Krida S., Younsi Z., Belouaggadia N., El Bouari A. (2025). Phosphogypsum valorization for sustainable building applications: Leveraging shape-stabilized phase change materials towards advanced thermal energy storage in paints. Constr. Build. Mater..

[105] Zhang J., Liu L., Huang W., Xu Y., Liu S., Ma J. (2025). Development and properties of microcapsule phase-change energy storage composite gypsum board. Constr. Build. Mater..

[106] Tan Q., Siroux M. (2025). Evaluation and optimization of PCM-integrated walls: Energy, exergy, environmental, and economic perspectives. Renew. Sustain. Energy Rev..

[107] Kheradmand M., Azenha M., de Aguiar J.L., Krakowiak K.J. (2014). Thermal behavior of cement based plastering mortar containing hybrid microencapsulated phase change materials. Energy Build..

[108] Giro-Paloma J., del Valle-Zermeño R., Fernández A., Chimenos J., Formosa J. (2016). Thermogravimetric study of a Phase Change Slurry: Effect of variable conditions. Appl. Therm. Eng..

[109] Zhou Y., Zheng S., Liu Z., Wen T., Ding Z., Yan J., Zhang G. (2020). Passive and active phase change materials integrated building energy systems with advanced machine-learning based climate-adaptive designs, intelligent operations, uncertainty-based analysis and optimisations: A state-of-the-art review. Renew. Sustain. Energy Rev..

[110] Pomianowski M., Heiselberg P., Zhang Y. (2013). Review of thermal energy storage technologies based on PCM application in buildings. Energy Build..

[111] Hunger M., Entrop A., Mandilaras I., Brouwers H., Founti M. (2009). The behavior of self-compacting concrete containing micro-encapsulated phase change materials. Cem. Concr. Compos..

[112] Wang Y., Li Q., Miao W., Su Y., He X., Strnadel B. (2022). The thermal performances of cement-based materials with different types of microencapsulated phase change materials. Constr. Build. Mater..

[113] Soudian S., Berardi U., Laschuk N. (2020). Development and thermal-optical characterization of a cementitious plaster with phase change materials and thermochromic paint. Sol. Energy.

[114] Xie S., Ma C., Ji Z., Wu Z., Si T., Wang Y., Wang J. (2023). Electromagnetic wave absorption and heat storage dual-functional cement composites incorporated with carbon nanotubes and phase change microcapsule. J. Build. Eng..

[115] Erkizia E., Strunz C., Dauvergne J.-L., Goracci G., Peralta I., Serrano A., Ortega A., Alonso B., Zanoni F., Düngfelder M. (2024). Study of paraffinic and biobased microencapsulated PCMs with reduced graphene oxide as thermal energy storage elements in cement-based materials for building applications. J. Energy Storage.

[116] Gu Y., Li Y., Ju G., Zheng T., Liang R., Sun G. (2023). PCM microcapsules applicable foam to improve the properties of thermal insulation and energy storage for cement-based material. Constr. Build. Mater..

[117] Zhao K., Wang J., Xie H., Guo Z. (2023). Microencapsulated phase change n-Octadecane with high heat storage for application in building energy conservation. Appl. Energy.

[118] Bre F., Caggiano A., Koenders E.A. (2022). Multiobjective Optimization of Cement-Based Panels Enhanced with Microencapsulated Phase Change Materials for Building Energy Applications. Energies.

[119] Gencel O., Sarı A., Subasi S., Bayram M., Danish A., Marasli M., Hekimoğlu G., Ustaoglu A., Ozbakkaloglu T. (2022). Light transmitting glass fiber reinforced cementitious composite containing microencapsulated phase change material for thermal energy saving. Constr. Build. Mater..

[120] Salihi M., El Mastouri M., El Fiti M., Harmen Y., Chebak A., Jama C., Chhiti Y. (2025). Calcium carbonate-shelled microencapsulated phase change materials in cement mortar: A pathway to enhancing energy efficiency in building envelopes. J. Energy Storage.

[121] Kumar D., Alam M., Sanjayan J., Haris M. (2023). Comparative analysis of form-stable phase change material integrated concrete panels for building envelopes. Case Stud. Constr. Mater..

[122] Gbekou F.K., Benzarti K., Boudenne A., Eddhahak A., Duc M. (2022). Mechanical and thermophysical properties of cement mortars including bio-based microencapsulated phase change materials. Constr. Build. Mater..

[123] Gbekou F.K., Belloum R., Chennouf N., Agoudjil B., Boudenne A., Benzarti K. (2024). Thermal performance of a building envelope including microencapsulated phase change materials (PCMs): A multiscale experimental and numerical investigation. Build. Environ..

[124] Cabeza L.F., Navarro L., Pisello A.L., Olivieri L., Bartolomé C., Sánchez J., Álvarez S., Tenorio J.A. (2020). Behaviour of a concrete wall containing micro-encapsulated PCM after a decade of its construction. Sol. Energy.

[125] Xue Y., da Silva C., Bishara N. (2024). Experimental and numerical performance analysis of an active cooling wall module equipped with micro-encapsulated phase change material. Energy Build..

[126] Lajimi N., Ben Taher N., Boukadida N. (2023). Numerical simulation of heat and mass transfer of a wall containing micro-encapsulated phase change concrete (PCC). Front. Environ. Sci..

[127] Tabares-Velasco P.C., Christensen C., Bianchi M. (2012). Verification and validation of EnergyPlus phase change material model for opaque wall assemblies. Build. Environ..

[128] Salihi M., El Fiti M., Harmen Y., Chhiti Y., Chebak A., M’Hamdi Alaoui F.E., Achak M., Bentiss F., Jama C. (2022). Evaluation of global energy performance of building walls integrating PCM: Numerical study in semi-arid climate in Morocco. Case Stud. Constr. Mater..

[129] Alam M., Jamil H., Sanjayan J., Wilson J. (2014). Energy saving potential of phase change materials in major Australian cities. Energy Build..

[130] Zhuang C.l., Deng A.Z., Chen Y., Li S.B., Zhang H.Y., Fan G.Z. Validation of veracity on simulating the indoor temperature in PCM light weight building by EnergyPlus. Proceedings of the International Conference on Intelligent Computing for Sustainable Energy and Environment.

[131] Kuznik F., Virgone J., Roux J.J. (2008). Energetic efficiency of room wall containing PCM wallboard: A full-scale experimental investigation. Energy Build..

[132] Chan A. (2011). Energy and environmental performance of building façades integrated with phase change material in subtropical Hong Kong. Energy Build..

[133] Campbell K.R., Sailor D.J. Phase change materials as thermal storage for high performance homes. Proceedings of the ASME International Mechanical Engineering Congress and Exposition.

[134] Dardouri S., Tunçbilek E., Khaldi O., Arıcı M., Sghaier J. (2023). Optimizing PCM integrated wall and roof for energy saving in building under various climatic conditions of mediterranean region. Buildings.

[135] Babaharra O., Choukairy K., Khallaki K., Hayani Mounir S. (2022). Numerical study of phase change material microencapsulated in a typical multilayer wall for a hot climatic zone. Heat Transf..

[136] Chen X.N., Xu B., Fei Y., Gan W.t., Pei G. (2023). Parameter optimization of phase change material and the combination of phase change material and cool paint according to corresponding energy consumption characteristics under various climates. Energy.

[137] Yu J., Qian C., Yang Q., Xu T., Zhao J., Xu X. (2023). The energy saving potential of a new ventilation roof with stabilized phase change material in hot summer region. Renew. Energy.

[138] Uribe D., Vera S. (2021). Impact of Phase Change Material (PCM) glazing on the energy consumption and solar radiation transmission in an office room located in a semi-arid climate: Analysis of a real-scale experiment. J. Phys. Conf. Ser..

[139] Luo Z., Liu X., Yang Q., Qu Z., Xu H., Xu D. (2023). Numerical study on performance of porous brick roof using phase change material with night ventilation. Energy Build..

[140] Jia J., Liu B., Ma L., Wang H., Li D., Wang Y. (2021). Energy saving performance optimization and regional adaptability of prefabricated buildings with PCM in different climates. Case Stud. Therm. Eng..

[141] Heim D., Krempski-Smejda M., Dellicompagni P.R., Knera D., Wieprzkowicz A., Franco J. (2021). Dynamics of melting process in phase change material windows determined based on direct light transmission. Energies.

[142] Ma Y., Li D., Yang R., Zhang S., Arıcı M., Liu C., Zhang C. (2022). Energy and daylighting performance of a building containing an innovative glazing window with solid-solid phase change material and silica aerogel integration. Energy Convers. Manag..

[143] Gao Y., Zheng Q., Jonsson J.C., Lubner S., Curcija C., Fernandes L., Kaur S., Kohler C. (2021). Parametric study of solid-solid translucent phase change materials in building windows. Appl. Energy.

[144] Wijesena R.N., Tissera N.D., Rathnayaka V., Rajapakse H., de Silva R.M., de Silva K.N. (2020). Shape-stabilization of polyethylene glycol phase change materials with chitin nanofibers for applications in “smart” windows. Carbohydr. Polym..

[145] Che-Pan M., Simá E., Ávila-Hernández A., Uriarte-Flores J., Vargas-López R. (2023). Thermal performance of a window shutter with a phase change material as a passive system for buildings in warm and cold climates of México. Energy Build..

[146] Roy A., Ullah H., Alzahrani M., Ghosh A., Mallick T.K., Tahir A.A. (2022). Synergistic effect of paraffin-incorporated In_2_O_3_/ZnO multifold smart glazing composite for the self-cleaning and energy-saving built environment. ACS Sustain. Chem. Eng..

[147] Mahdaoui M., Hamdaoui S., Msaad A.A., Kousksou T., El Rhafiki T., Jamil A., Ahachad M. (2021). Building bricks with phase change material (PCM): Thermal performances. Constr. Build. Mater..

[148] Dabiri S., Mehrpooya M., Nezhad E.G. (2018). Latent and sensible heat analysis of PCM incorporated in a brick for cold and hot climatic conditions, utilizing computational fluid dynamics. Energy.

[149] Al-Yasiri Q., Szabo M. (2021). Effect of encapsulation area on the thermal performance of PCM incorporated concrete bricks: A case study under Iraq summer conditions. Case Stud. Constr. Mater..

[150] Rai A.C. (2021). Energy performance of phase change materials integrated into brick masonry walls for cooling load management in residential buildings. Build. Environ..

[151] Mukram T.A., Daniel J. (2024). Performance evaluation of a novel cement brick filled with micro-PCM used as a thermal energy storage system in building walls. J. Energy Storage.

[152] Fraine Y., Seladji C., Aït-Mokhtar A. (2019). Effect of microencapsulation phase change material and diatomite composite filling on hygrothermal performance of sintered hollow bricks. Build. Environ..

[153] Buczkowska K. (2023). Hydrophobic protection for building materials. Superhydrophobic Coating-Recent Advances in Theory and Applications.

[154] Kasai M., Suzuki A., Egami Y., Nonomura T., Asai K. (2023). A platinum-based fast-response pressure-sensitive paint containing hydrophobic titanium dioxide. Sens. Actuators A Phys..

[155] Fernando T.L.D., Ray S., Perera J., Swift S., Simpson M.C. (2023). Photocatalytic and Protective Thin Films for Enhanced Self-cleaning Activity and Durability of Painted Steel Roofings. ChemistrySelect.

[156] Ghayedhosseini A., Baneshi M., Fathi A. (2024). Simulating the Individual and Combined Utilization of Cool Paints and Phase Change Materials for Enhancing Energy Efficiency in High-Rise Office Buildings. Int. J. Energy Res..

[157] Qin M., Xiong F., Aftab W., Shi J., Han H., Zou R. (2022). Phase-change materials reinforced intelligent paint for efficient daytime radiative cooling. Iscience.

[158] Jeong S.G., Chang S.J., Wi S., Kang Y., Kim S. (2016). Development and performance evaluation of heat storage paint with MPCM for applying roof materials as basic research. Energy Build..

[159] Han X., Li Y., Yuan L., Wang Q., Zhang H., Lian H., Zhang G., Xiao L. (2017). Experimental study on effect of microencapsulated phase change coating on indoor temperature response and energy consumption. Adv. Mech. Eng..

[160] Gao F., Xiao X., Shu Z., Zhong K., Wang Y., Li M. (2024). Investigation of Thermoregulation Effect of Stabilized Phase Change Gypsum Board with Different Structures in Buildings. Sustainability.

[161] Zhou H., Puttige A.R., Nair G., Olofsson T. (2024). Thermal behaviour of a gypsum board incorporated with phase change materials. J. Build. Eng..

[162] Naikwadi A.T., Samui A.B., Mahanwar P. (2021). Experimental investigation of nano/microencapsulated phase change material emulsion based building wall paint for solar thermal energy storage. J. Polym. Res..

[163] Xu C., Zhang Y., Qiu D. (2024). The Regulation of Temperature Fluctuations and Energy Consumption in Buildings Using Phase Change Material–Gypsum Boards in Summer. Buildings.

[164] Wadee A., Achanta P., Walker P., McCullen N., Ferrandiz-Mas V. (2023). Novel gypsum based plasters with phase change material impregnated lightweight aggregates for energy efficient retrofitting. Constr. Build. Mater..

[165] Kumar N., Rathore P.K.S., Pal A.K. (2023). Experimental investigation of composite gypsum board integrated with phase change material for improved thermal energy storage. Mater. Today Proc..

[166] Nowak K., Kisilewicz T., Berardi U., Zastawna-Rumin A. (2023). Thermal performance evaluation of a PCM-integrated gypsum plaster board. Mater. Proc..

[167] Shukla N., Kosny J. (2015). DHFMA method for dynamic thermal property measurement of PCM-integrated building materials. Curr. Sustain. Renew. Energy Rep..

[168] Bake M., Shukla A., Liu S. (2021). Development of gypsum plasterboard embodied with microencapsulated phase change material for energy efficient buildings. Mater. Sci. Energy Technol..

[169] Errebai F.B., Chikh S., Derradji L., Amara M., Younsi Z. (2021). Optimum mass percentage of microencapsulated PCM mixed with gypsum for improved latent heat storage. J. Energy Storage.

[170] Bravo J.P., Venegas T., Correa E., Álamos A., Sepúlveda F., Vasco D.A., Barreneche C. (2020). Experimental and computational study of the implementation of mPCM-modified gypsum boards in a test enclosure. Buildings.

[171] Gencel O., Bayram M., Subaşı S., Hekimoğlu G., Sarı A., Ustaoglu A., Marasli M., Ozbakkaloglu T. (2023). Microencapsulated phase change material incorporated light transmitting gypsum composite for thermal energy saving in buildings. J. Energy Storage.

[172] Hamooleh M.B., Torabi A., Baghoolizadeh M. (2024). Multi-objective optimization of energy and thermal comfort using insulation and phase change materials in residential buildings. Build. Environ..

[173] Al-Yasiri Q., Szabó M. (2023). Building envelope-combined phase change material and thermal insulation for energy-effective buildings during harsh summer: Simulation-based analysis. Energy Sustain. Dev..

[174] Khan M., Khan M.M., Irfan M., Ahmad N., Haq M.A., Khan I., Mousa M. (2022). Energy performance enhancement of residential buildings in Pakistan by integrating phase change materials in building envelopes. Energy Rep..

[175] Arumugam P., Ramalingam V. (2024). Thermal comfort enhancement of office buildings located under warm and humid climate through phase change material and insulation coupled with natural ventilation. Sustain. Energy Technol. Assess..

[176] Abbas H.M., Jalil J.M., Ahmed S.T. (2021). Experimental and numerical investigation of PCM capsules as insulation materials inserted into a hollow brick wall. Energy Build..

[177] Zhilyaev D., Fachinotti V.D., Zanoni F., Ortega A., Goracci G., Mankel C., Koenders E.A.B., Jonkers H.M. (2023). Early-stage analysis of a novel insulation material based on MPCM-doped cementitious foam: Modelling of properties, identification of production process hotspots and exploration of performance trade-offs. Dev. Built Environ..

[178] Nouhaila B., Mouhib T., Bendriaa F. (2022). Creation of energy-efficient building envelope based on phase-change materials (PCM). Eximia.

[179] Guermat Z., Kabar Y., Kuznik F., Boukelia T. (2024). Numerical investigation of the integration of new bio-based PCM in building envelopes during the summer in Algerian cities. J. Energy Storage.

[180] Berrocal D., Aranda R., Santamaría S., Vigil A., Austin M.C. (2022). Phase Change as a Passive Strategy: Evaluation of the Thermal-energy Performance in Buildings in Panama. Rev. I+D Tecnol..

[181] Benachir N., Bendriaa F., Mouhib T. Generality About Modeling the Building Envelope With the Solar Ventilation and PCM in TRNSYS and Energy Plus. https://papers.ssrn.com/sol3/papers.cfm?abstract_id=4545853.

[182] Aghoei M.M., Astanbous A., Khaksar R.Y., Moezzi R., Behzadian K., Annuk A., Gheibi M. (2024). Phase change materials (PCM) as a passive system in the opaque building envelope: A simulation-based analysis. J. Energy Storage.

[183] Beiranvand M., Mohaghegh M.R. (2021). Energy analysis and simulation of PCM-enhanced building envelopes in commercial buildings: A case study. Energy Storage.

[184] Hagenau M., Jradi M. (2020). Dynamic modeling and performance evaluation of building envelope enhanced with phase change material under Danish conditions. J. Energy Storage.

[185] Kulumkanov N., Memon S.A., Khawaja S.A. (2024). Evaluating future building energy efficiency and environmental sustainability with PCM integration in building envelope. J. Build. Eng..

[186] Laasri I.A., Charai M., Es-sakali N., Mghazli M.O., Outzourhit A. (2024). Evaluating passive PCM performance in building envelopes for semi-arid climate: Experimental and numerical insights on hysteresis, sub-cooling, and energy savings. J. Build. Eng..

